# Application of modified artificial hummingbird algorithm in optimal power flow and generation capacity in power networks considering renewable energy sources

**DOI:** 10.1038/s41598-023-48479-6

**Published:** 2023-12-05

**Authors:** Marwa M. Emam, Essam H. Houssein, Mohamed A. Tolba, Magdy M. Zaky, Mohammed Hamouda Ali

**Affiliations:** 1 Faculty of Computers and Information, Minia University, Minia, Egypt; 2 Reactors Department, Nuclear Research Center, Egyptian Atomic Energy Authority (EAEA), Cairo, 11787 Egypt; 3 Engineering Department, Nuclear Research Center, ETRR-2, Atomic Energy Authority (EAEA), Cairo, 11787 Egypt; 4 Department of Electrical Engineering, Faculty of Engineering, Al-Azhar University, Cairo, 11651 Egypt

**Keywords:** Energy science and technology, Engineering, Mathematics and computing

## Abstract

Today's electrical power system is a complicated network that is expanding rapidly. The power transmission lines are more heavily loaded than ever before, which causes a host of problems like increased power losses, unstable voltage, and line overloads. Real and reactive power can be optimized by placing energy resources at appropriate locations. Congested networks benefit from this to reduce losses and enhance voltage profiles. Hence, the optimal power flow problem (OPF) is crucial for power system planning. As a result, electricity system operators can meet electricity demands efficiently and ensure the reliability of the power systems. The classical OPF problem ignores network emissions when dealing with thermal generators with limited fuel. Renewable energy sources are becoming more popular due to their sustainability, abundance, and environmental benefits. This paper examines modified IEEE-30 bus and IEEE-118 bus systems as case studies. Integrating renewable energy sources into the grid can negatively affect its performance without adequate planning. In this study, control variables were optimized to minimize fuel cost, real power losses, emission cost, and voltage deviation. It also met operating constraints, with and without renewable energy. This solution can be further enhanced by the placement of distributed generators (DGs). A modified Artificial Hummingbird Algorithm (mAHA) is presented here as an innovative and improved optimizer. In mAHA, local escape operator (LEO) and opposition-based learning (OBL) are integrated into the basic Artificial Hummingbird Algorithm (AHA). An improved version of AHA, mAHA, seeks to improve search efficiency and overcome limitations. With the CEC'2020 test suite, the mAHA has been compared to several other meta-heuristics for addressing global optimization challenges. To test the algorithm's feasibility, standard and modified test systems were used to solve the OPF problem. To assess the effectiveness of mAHA, the results were compared to those of seven other global optimization algorithms. According to simulation results, the proposed algorithm minimized the cost function and provided convergent solutions.

## Introduction

The optimal power flow (OPF) minimizes generation costs, power losses, and voltage stability while adhering to system restrictions^1^. OPF is a large-scale, nonlinear, constrained, nonconvex optimization problem in power systems. This problem has been addressed with linear programming, nonlinear programming, quadratic programming, Newton, and interior point methods. These traditional methods, however, have certain limitations and require specific theoretical assumptions. Consequently, they are limited in their optimization abilities^2–4^. Despite this, solving the OPF problem remains a popular and challenging task.

Researchers have recently discovered that metaheuristic algorithms, which are all-purpose and straightforward to use, can tackle challenging real-world problems. Because metaheuristics are very accurate and straightforward, they have drawn much attention in various challenging optimization issues in engineering, communications, medical, and social sciences^5^. Moreover, metaheuristic algorithms are also used to improve solutions for a variety of problems, such as global optimization^6^, energy applications^7^, power flow systems^8^, image segmentation^9, 10^, deep learning-based classification^11^, economic emission dispatch (EED) problems^12^, and feature selection^13, 14^. In contrast to deterministic algorithms, metaheuristic algorithms employ specialized operators and randomly generated search agents to find optimal solutions. Natural phenomena, such as swarms and social behavior, evolutionary principles, and physical theories, inspire these operators. In general, metaheuristic algorithms fall into three categories: swarm methods, which simulate animals, birds, and humans' social behavior; evolutionary methods; and natural phenomena algorithms^15^.

Metaheuristic methods have gained popularity in solving complex OPF problems using population-based techniques. Researchers have studied these methods with only thermal power generators^16^. The traditional OPF issue was solved by Kumari^17^ using an upgraded genetic algorithm (GA), and Khunkitti^18^ utilized a hybrid dragonfly and PSO technique for minimizing fuel loss, emissions, and power loss. Based on FACTS devices, Basu^19^ proposed a DE method that considers generating costs, emissions, and power losses to overcome OPF issues. Singh^20^ overcomes IEEE-30 and IEEE-118 OPF problems using PSO and an aging leader and challenger. An adapted Sine–Cosine algorithm with Levy flights was used in Attia's^21^ solution to the OPF problem.

It is apparent from the literature that traditional OPF issues only consider thermal power sources. Since fuel prices have increased and environmental concerns have been heightened, a stochastic OPF has been necessary to optimize renewable energy sources^22, 23^. However, wind energy has been incorporated in a variety of ways, such as the use of genetic algorithms by Liu^24^, the use of a fuzzy selection mechanism by Hetzer^26^, and the use of hybrid flower pollination by Dubey^27^. In addition, other studies have considered the stochastic nature of wind power and the variable nature of its loads. As examples, Miguel examined the impact on operating costs^25^, Kusakana included solar photovoltaic, wind, diesel generators, and batteries^28^, and Partha used a historical parameter adaptation approach to combine wind and solar power^30^.

Furthermore, the Gray Wolf Optimizer method was applied to the IEEE-30 bus and IEEE-57 bus systems to combine thermal power, wind energy, and solar energy^31^. In addition, Arsalan used the Krill Herd algorithm to solve OPF problems relating to wind energy generation under uncertainty in both the IEEE-30 bus system and the IEEE-57 bus system^32^. In modified IEEE 30-bus and IEEE 57-bus systems^33^, Mohd applied the Barnacles Mating Optimizer method to the OPF problem with stochastic wind energy. Shuijia Li^34^ presented a penalty constraint handling strategy for solving OPF in an IEEE-30 bus system utilizing an enhanced adaptive DE. However, an overview of soft computing contributions to OPF literature can be found in Table 1.

**Table 1:** Literature contribution.

Year	References	Method	Description
2021	^28^	Multi-objective Quasi-Reflected Jellyfish Search Optimizer (MOQRJFS)	MOQRJFS was developed for solving multi-dimensional Optimal Power Flow (MDOPF) issues with diverse objectives that display the minimization of economic fuel cost, total emissions, and active power loss while satisfying operational constraints
2020	^27^	Adaptive grasshopper optimization (AGO) algorithm	As part of the economic dispatch issue, an AGO algorithm had been devised to the optimal power flow (OPF) problem with the optimal incorporation of a center-node unified power flow controller (C-UPFC)
2021	^35^	Modified crow search optimizer (MCSO)	A modified CSO applies in IEEE 30 bus, IEEE 118-bus and West Delta power grid (WDPG) systems to solve various OPF issues
2017	^30^	Incorporation of OPF with stochastic wind and solar power	The OPF issue was solved by considering a differential evolution algorithm in a small IEEE-30 bus system. A successful adaptation technique based on the algorithm's history was employed to incorporate intermittent solar and wind power generation
2019	^36^	Improved moth flame optimization (IMFO)	Based on the results of this study, an improved moth flame optimization (IMFO)approach was introduced as a strategy for determining the OPF on 15 case studies in terms of different single and multi‐objective functioninto in the IEEE 30-bus, 57 bus and 118 bus systems
2017	^37^	Biogeography-based optimization (BBO) and grey wolf optimization (GWO)	There were two algorithms presented, BBO and GWO, that were used to solve multi-constrained OPF problems in the power system. Different conditions were used to test the algorithms' performance on both the IEEE 30-bus and the 9-bus systems
2018	^38^	Differential evolution algorithm integrated with effective constraint-handling techniques (ECHT-DE)	ECHT-DE was utilized to address the OPF issue. As part of the validation process, the approach was applied to the OPF in IEEE 30 bus , IEEE 57 bus and IEEE 118 bus systems while considering objective functions based on operational and economic indicators for the power system
2018	^39^	Stud krill herd optimizer (SKH)	The SKH optimizer solved OPF issues in IEEE 14, 30, and 57-bus networks. Several objective functions were considered in the proposed algorithm, including minimizing total production cost with and without the effect of valve point loading, active power loss, L-index, and emission pollution
2018	^21^	Developed Grey Wolf Optimizer (DGWO)	DGWO was utilized to address the OPF issue. As part of the validation process, the approach was applied to the OPF in IEEE 30 bus systems while considering objective functions based on operational and economic indicators for the power system
2019	^40^	Hybrid Firefly and krill herd method (FKH)	To address the OPF issue, the researchers utilized a revised version of the FKH optimizer and considered different types of single-objective and multi-objective functions: reducing fuel costs, reducing emissions, reducing transmission power losses, and improving voltage profiles. The FKH has been applied to IEEE 30 bus systems
2020	^31^	GWO Optimizer	The OPF issue was solved using the GWO Optimizer, which integrated intermittent solar and wind power generation without utilizing actual wind speed data
2020	^32^	Krill Herd Algorithm (KHA)	The OPF issue with FACTS devices and stochastic wind power generation was solved considering the KHA optimizer for one scenario where wind generation costs were overestimated or underestimated
2020	^41^	Modified Artificial Bee Colony (MABC)	The OPF has been addressed using MABC. With this method, four distinct objective functions have been minimized within the IEEE 30-bus system. These functions include total fuel cost for thermal units, total transmission losses, total fossil fuel emissions, and total voltage deviation on load nodes
2021	^29^	Moth-Flame Optimizer (MFO)	Three objective functions were solved simultaneously deemed minimizing fuel cost, transmission loss, and voltage deviation minimization using a weighted factor
2021	^33^	Barnacles mating optimizer (BMO)	The OPF issue has been achieved by utilizing the BMO that incorporated FACTS devices and stochastic wind power generation in a one-scenario. This technique also considered the costs associated with overestimating and underestimating wind power generation
2021	^42^	Rao Algorithm	Using the Rao algorithm, OPF problems with both technical and economic objectives can be addressed within the standard IEEE 30-bus, 57-bus, and 118-bus networks
2021	^43^	Multi-Objective Backtracking Search Algorithm (MOBSA)	The OPF issue in power systems was addressed using MOBSA technique. Multi-objective functions, such as fuel cost, power loss, and voltage deviation, are considered in this technique. As part of the standard BSA methodology, a fuzzy membership technique was utilized to identify the most likely compromise results among the derived Pareto optimal solutions. Three IEEE power systems were employed to determine and verify the effectiveness of the MOBSA approach: the small network 30-bus, the medium network 57-bus, and the large network 118-bus test systems
2021	^44^	Firefly Algorithm (FA)	The OPF issue was addressed using the FA technique. Newton–Raphson was used to calculate the real power loss when performing the load flow analysis. To optimize the control variables, including the magnitudes of generator bus voltages, transformer tap settings, and generator output active power, the FA methodology was applied. As a result, real power losses were minimized in the transmission system. In the context of IEEE 14-bus and 30-bus systems, MATLAB software was used to evaluate the proposed approach
2021	^45^	Multi Objective Particle Swarm Optimizer (MOPSO)	To address the constrained multi-objective OPF issue in power systems with conflicting objectives, the MOPSO technique has been implemented. The best optimal solution from the Pareto optimal set was extracted using fuzzy set theory and presented to the operator. The effectiveness and applicability of the introduced methodology were evaluated considering the IEEE 30-bus network
2021	^46^	Jellyfish Search Optimizer (JSO)	On the modified IEEE 30-bus grid, the JSO technique has been proposed to overcome the OPF problems
2022	^47^	Jellyfish Optimizer (JFO)	The JFO optimizer was implemented to solve the OPF considering fuel costs, emissions and losses. A Quasi-Reflection (QR) is integrated with JFOA in solving the OPF problem
2022	^48^	gorilla troops optimization technique (GTOT)	In order to solve OPF problems that contain single and multi-objective objectives, GTOT methodology was developed. In order to evaluate the algorithm, the IEEE 30-bus system was used
2022	^49^	Archimedes optimization algorithm (AOA)	An AOA algorithm using non-dominated sorting and a constraint handling technique is designed to solve the OPF issue renewable energy sources (RES). The efficacy of this approach is demonstrated by using it to solve problems on the standard and modified IEEE 30 bus networks. These tests also confirm the approach's effectiveness in handling significant dimensional problems
2023	^50^	Improved Cross-Entropy Method (CGSCE)	An Improved Cross-Entropy (CE) approach integrated with a chaotic operator (CGSCE) was introduced to tackle the OPF issue. Different target functions were evaluated on the IEEE-30 bus and IEEE 57 bus test system

Although these algorithms were aimed at solving the same OPF issues, their optimization functions were different, which led to various optimized solutions resulting in different optimization performance that is assessed by the quality of the optimum solution and the convergence time. Even though many metaheuristic methodologies have shown satisfactory outcomes, optimization problems have become more challenging due to the increasing number of variables and constraints that can be optimized. However, metaheuristic optimization algorithms cannot always obtain the optimal global solution, regardless of their advantages. Further, no algorithm is suitable for solving all variants of the OPF problem due to the variability of objectives used to formulate it. It is, therefore, necessary to develop metaheuristic algorithms capable of handling various OPF formulations very effectively. In order to address current optimization challenges, combining two or more metaheuristics and modifying or improving existing algorithms is necessary. This procedure is known as hybridization^51^.

Nevertheless, selecting hybridization algorithms that will enhance optimization performance is essential. Thus, choosing an algorithm is an important step in the process, typically based on its performance. It is therefore recommended to study more recent algorithms and features to develop a more effective algorithm for solving OPF problems. Particularly, the artificial hummingbird algorithm (AHA) has attracted great interest. Despite the promising results achieved by the AHA method, this method is not entirely impervious to metaheuristic flaws. Several studies have pointed out the algorithm's slow convergence speed and tendency to get trapped in local optima. They also discuss the significant effect algorithm parameters have on algorithm performance and the inadequacy of exploration and exploitation. Hence, this paper suggests a modified artificial hummingbird algorithm (mAHA) that addresses these limitations by integrating the local escape operator (LEO) and opposition-based learning (OBL) into the basic AHA.

In this paper, we introduce a novel and enhanced approach to address the challenges in solving the Optimal Power Flow (OPF) problem. While various metaheuristic algorithms have shown promise in tackling OPF problems, they often face limitations, such as slow convergence speed and susceptibility to local optima. This paper presents a significant contribution in the form of the modified Artificial Hummingbird Algorithm (mAHA), which effectively addresses these limitations by integrating the local escape operator (LEO) and opposition-based learning (OBL) into the basic AHA. The key objective of this paper is to combine OPF with Renewable Energy Sources (RESs) to optimize scheduled power from RESs and generating power from thermal units, thereby minimizing the total operational cost. To validate the effectiveness of our proposed approach, we apply the mAHA algorithm to standard IEEE 30, and 118 bus systems for solving traditional OPF issues, as well as a modified IEEE-30 bus system that incorporates RES. Our contributions include developing and testing the mAHA algorithm on a range of benchmark functions, comparing it with established metaheuristic algorithms, and demonstrating its efficacy in integrating RES into the OPF problem. These contributions collectively provide a comprehensive and innovative solution to enhance the optimization of power systems. The main contributions of this work can be summarized in the following items:

- This paper proposed a modified mAHA algorithm and tested through unimodal, multimodal, and composite benchmark functions .
- The performance of mAHA compared to competitors is demonstrated using the CEC'2020 benchmark test problems.
- Present four different objective functions for formulating the real-world problem called OPF problem.
- mAHA converts the multi-objective function, which includes fuel costs, power losses, voltage deviations, and emissions, into a single-objective function based on price and weighting factors.
- Several benchmark problems from the metaheuristic literature are tested, including IEEE 30, and 118 bus grids, to assess the effectiveness and scalability of the proposed algorithm.
- A comparison is made between the performance of mAHA and various established meta-heuristic algorithms to verify its validity and effectiveness, including the Whale optimization algorithm (WOA), Sine cosine algorithm (SCA), Tunicate swarm algorithm (TSA), Slime mould algorithm (SMA), Harris hawks optimization (HHO), RUNge Kutta optimization algorithm (RUN), and the original Artificial Hummingbird Algorithm (AHA).
- Efficient Integration of renewable energy sources (RES) and external electric grid (EEG) has been suggested to overcome the OPF problem.
- The mAHA technique is applied to a modified version of the IEEE 30-bus grid that includes the optimum allocation of RES via the OPF issue. This test demonstrates the superiority of the suggested methodology over other state-of-the-art metaheuristic techniques.

After the introduction section, the presented paper is constructed in the following sections: Section "Preliminaries" provides the mathematical model for the basic AHA algorithm required to construct the proposed modified algorithm, the OBL strategy, and the Local Escaping Operator (LEO). Section "The proposed mAHA algorithm" provides the mathematical model of the proposed mAHA algorithm. Section "Application of mAHA: optimal power flow and generation capacity" introduces the OPF mathematical formulation model. Section "Evaluated results and discussion" discusses the design findings. The discussion contains the performance results of the proposed mAHA on CEC'2020 benchmark functions. It also contains the results of the proposed mAHA based on the OPF problem. Section “Conclusion” presents this paper's conclusion and future work.

## Preliminaries

This section will cover the fundamental methods needed to construct the proposed method. We will comprehensively explain the mathematical model of the Artificial Hummingbird Algorithm (AHA), the OBL approach, and the local escaping operator (LEO) technique.

### Artificial hummingbird algorithm (AHA)

Based on the behavior of hummingbirds, the AHA technique was developed to solve real-world problems^52^. The hummingbird is an incredible creature among the smallest birds in the world. By replicating the axial, diagonal, and omnidirectional flight techniques of hummingbirds, the AHA algorithm seeks to replicate the flight abilities and intelligent foraging strategies of these birds. Foraging strategies, memory capacity, and flight abilities of hummingbirds have been incorporated into the algorithm. Furthermore, the AHA algorithm incorporates guided foraging, territorial foraging, and migrating foraging techniques. Tracking food sources mimics hummingbird memory by using a visiting table. As a result of the AHA algorithm, the following three main elements are explained:

- **Food sources**: When selecting food sources, hummingbirds consider factors such as the quality and content of nectar in individual flowers, the rate at which nectar is refilled, and the last time they visit the flowers. In the AHA algorithm, each food source is assumed to have the same type and quantity of flowers, represented by a solution vector. Its fitness value indicates the nectar-refilling rate. A food source with a higher nectar-refilling rate will have higher fitness.
- **Hummingbirds**: Every hummingbird is given a unique food source to feed from, and the bird and the food source are positioned in a specific location. A hummingbird can remember the exact location of the food source and the frequency of nectar replenishment for that particular source. This information can be communicated to other hummingbirds in the population. Moreover, each hummingbird can recall its last visit to a particular food source.
- **Visit table**: A table is maintained to record the visit history of different hummingbirds to each food source, indicating the duration since a particular bird last fed from it. When a hummingbird decides to feed, it prioritizes a food source with a high visit level for that specific bird. If multiple food sources have the same highest visit level, the bird selects the one with the highest nectar-refilling rate to obtain more nectar. This visit table helps each hummingbird to locate its preferred food source. Typically, the visit table is updated after each feeding loop.

#### AHA mathematical model

The three mathematical representations simulating three foraging behaviors of hummingbirds: guided foraging, territorial foraging, and migrating foraging are presented as follows:

**Step 1: Initialization**

A population of N hummingbirds is established on N food sources, randomly initialized as Eq. (1)

1 $$ Xb_{i} = lb_{i} + rand \times (ub_{i} - lb_{i} );\:\:i = 1,2,...,N $$

where $Xb_{i}$ denotes the solution in a population set of N. $lb_{i}$ and $ub_{i}$ are the lower and upper boundaries, respectively.

The visit table of food sources is initialized in Eq. (2)

2 $$ V_{t} = \left\{ {\begin{array}{*{20}l} 0 \hfill & {i \ne j} \hfill \\ {null} \hfill & {i = j} \hfill \\ \end{array} } \right.\:\:\:\:i = \{ 1,2,...,N\} ;j = \{ 1,2,...,N\} $$

**Step 2: Guided foraging**

To exhibit guided foraging behavior, the hummingbird must identify food sources with the highest visit level and choose the one with the most rapid nectar replenishment as its target. Once identified, the bird can navigate toward the desired food source. The AHA algorithm incorporates three flight skills to direct the search space during foraging: omnidirectional, diagonal, and axial flights. The axial flight is described by Eq. (3).

3 $$ D_{i} = \left\{ {\begin{array}{*{20}l} 1 \hfill & {i = randi([1,d])} \hfill \\ 0 \hfill & {otherwise} \hfill \\ \end{array} } \right.\:\:\:\:i = \{ 1,2,...,d\} $$

The diagonal flight is calculated by Eq. (4)

4 $$ D_{i} = \left\{ {\begin{array}{*{20}l} 1 \hfill & {i = G(j)\:,j \in [1,k],G = randperm(l),\:l \in [2,[r1 \times (d - 2)] + 1]} \hfill \\ 0 \hfill & {otherwise} \hfill \\ \end{array} } \right. $$

The omnidirectional flight is calculated by Eq. (5)

5 $$ D_{i} = 1,\:\:i = \{ 1,2,...,d\} $$

where $randi([1,d])$ obtains an integer random from 1 to d, $randperm(l)$ generates a random permutation of integers from 1 to l, and $r1$ is a random number between [0, 1].

Using different flying patterns, Eq. (6) simulates directed foraging behavior by allowing each food source to update its location relative to the target food source. It also depicts the foraging activity of hummingbirds.

6 $$ \zeta (t + 1) = Xb_{i,targ} (t) + a \times D \times (Xb_{i} (t) - Xb_{i,targ} (t)) $$

7 $$ a\sim N(0,1) $$

Where $Xb_{i} (t)$ denotes the $i^{{{\text{th}}}}$ position, $Xb_{i,targ} (t)$ denotes the position of the target food source, and a denotes the guided vector.

The updating positions are applied using Eq. (8).

8 $$ Xb_{i} (t + 1) = \left\{ {\begin{array}{*{20}l} {Xb_{i} (t)} \hfill & {f(Xb_{i} (t)) \le f(\zeta (t + 1))} \hfill \\ {\zeta (t + 1)} \hfill & {f(Xb_{i} (t)) > f(\zeta (t + 1))} \hfill \\ \end{array} } \right. $$

where $f(.)$ denotes the objective function. Equation (8) illustrates that if the candidate food source's nectar-refilling rate is greater than the current one, the hummingbird discards the current food source and remains at the candidate food source calculated using Eq. (6) for feeding.

The visit table records the time elapsed since a specific hummingbird last visited each food source, and a more extended period between visits indicates a higher visit level. Each hummingbird seeks the food source(s) that receives the most visitors. If two or more sources have an equal number of visits, the bird chooses the one with the highest rate of nectar replenishment as its target food source. Each bird navigates to its intended food source using Eq. (6). When a hummingbird uses Eq. (6) to guide its foraging during each iteration, the visit levels of other food sources visited by that specific bird are increased by 1. In contrast, the visit level of the target food source visited is set to 0. A hummingbird can engage in guided foraging with a guide to reach its preferred food source, then remain at the new food source until a better nectar-refilling rate (solution) or food quality (deterioration) becomes available.

The following schema illustrates AHA's guided foraging method:9

**Step 3: Territorial foraging**

During this step, a hummingbird can migrate to a nearby location within its territory, where it may find a new food source that could be a better solution than the current one. The local search of hummingbirds in the territorial foraging strategy is modeled using Eq. (10), which helps to identify a candidate food source by:

10 $$ \zeta (t + 1) = Xb_{i} (t) + b \times D \times Xb_{i} (t) $$

11 $$ b\sim N(0,1) $$

Where b is a geographic variable, the visit table has to be updated following the territorial foraging approach. The following diagram illustrates AHA's territorial foraging strategy:12

**Step 4: Migration foraging**

The hummingbird at the food source with the lowest rate of nectar replenishment will randomly move to a new food source established in the whole search space once the number of iterations exceeds the predefined value of the migration coefficient. A hummingbird's foraging trip from the source with the lowest nectar replenishment rate can be modeled using Eq. (13).

13 $$ X_{wors} = lb + rand \times (ub - lb) $$

where $X_{wors}$ denotes the food source with the worst nectar-refilling rate. Equation (14) illustrates the migrating foraging strategy of AHA.14

A visiting table and a set of random solutions are created to summarize the AHA algorithm’s process. Each iteration has a 50% probability of carrying out territorial or guided foraging. Hummingbirds use guided foraging to travel to the food sources they prefer, which are determined by the frequency of their visits and the rate at which the nectar is replenished. However, due to territorial foraging, hummingbirds are forced to disturb their local populations. They are foraging while migration begins after 2n iterations. Three flight abilities—omnidirectional, diagonal, and axial—are used in the three foraging tasks. All operations are carried out interactively until the stopping criteria are met**.** The pseudo-code for the AHA procedure is provided in Algorithm 1.

### Opposition-based learning (OBL)

The OBL technique is an efficient method for avoiding stagnation in potential solutions. HR developed it. Tizhoosh^53^ to enhance the search mechanism's exploitation ability. When using meta-heuristic algorithms, convergence usually happens quickly when initial solutions are close to the optimal position, but slower convergence is expected otherwise. However, the OBL technique can discover more valuable solutions in opposite search regions that may be closer to the global optimum. To achieve this, the OBL searches in both directions of the search space. One of the initial solutions is used for both directions, while the opposite solution represents the other. The OBL then selects the most appropriate solutions from all solutions found.

**Opposition number:** The concept of opposite numbers represents opposition-based learning. An opposition-based number can be described as follows. Lets consider $Q_{0}$ it a real number on an interval: $Q_{0} \in [a,b]$ the opposite number $Q_{0}$ is defined by Eq. (15).

15 $$ \overline{Q}_{0} = a + b - Q_{0} $$

Equations (16) and (17) identify the opposite point in D-dimensional space.

16 $$ {\text{Q}} = {\text{q}}_{1} ,{\text{q}}_{2} ,{\text{q}}_{3} , \ldots ,{\text{q}}_{D} $$

17 $$ \overline{{\text{Q}}} = [\overline{{\text{Q}}}_{1} ,\overline{{\text{Q}}}_{2} ,\overline{{\text{Q}}}_{3} ,...,\overline{{\text{Q}}}_{D} ] $$

**Algorithm 1:**
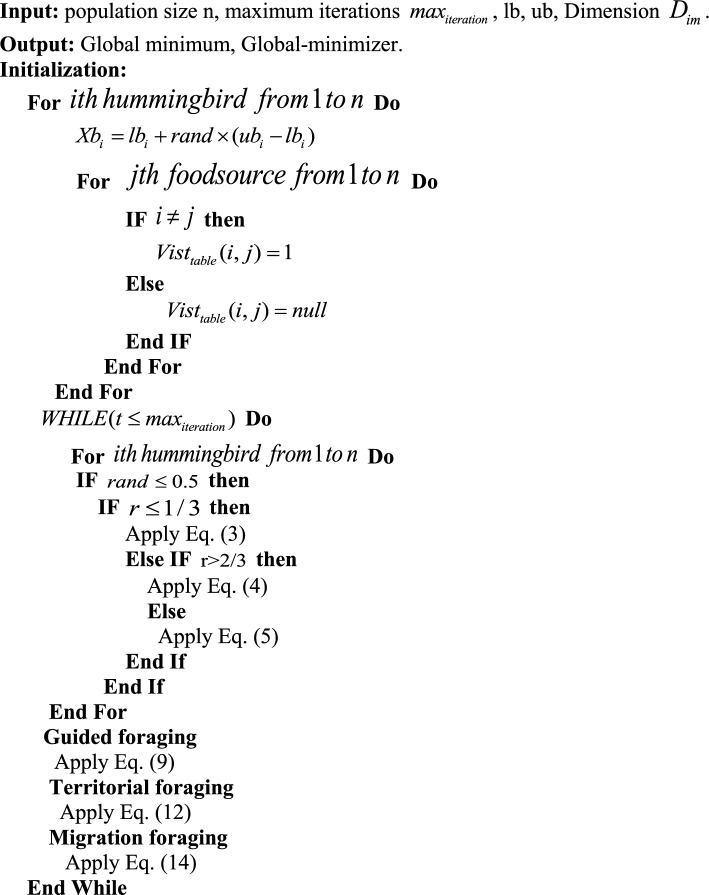
Pseudo-code of the AHA algorithm.

The items in $\overline{{\text{Q}}}$ are computed by Eq. (18)

18 $$ \overline{{\text{Q}}}_{k} = a_{k} + b_{k} - Q_{k} \,\,\,where\,\,k = 1,2,3,...,D $$

**Opposition-based optimization:** In the optimization strategy, the opposite value $\overline{Q}_{0}$ is replaced by the corresponding $Q_{0}$ based on the objective function. If $Q_{0}$ is more suitable $f(\overline{Q}_{0} )$, then $Q_{0}$ not changed; otherwise, the solutions of the population are updated based on the best value of $Q$ and $\overline{Q}_{0}$^54^.

### Local escaping operator (LEO)

The LEO is a technique proposed in^55^ that is utilized to enhance the effectiveness of the Gradient-based optimizer (GBO) algorithm in resolving complex real-world issues. Its purpose is to explore new areas necessary for finding solutions to challenging problems. By changing the position of solutions based on specific criteria, LEO improves the quality of the solutions and prevents the algorithm from being trapped in local optima. LEO selects new solutions ($X_{LEO}^{H}$) by utilizing various techniques, such as the best position* (*$Xb_{best}$), two randomly chosen solutions $X1_{r1}^{m}$ *and *$X2_{r2}^{m}$, two other randomly selected solutions ($Xb_{r1}^{m}$ *and *$Xb_{r2}^{m}$), and a newly generated random solution ($X_{k}^{m}$). Thus, the solution $X^{H}_{LEO}$ can be obtained using the following:

19 $$ \begin{gathered} {\mathbf{IF}} \hfill \\ X_{LEO}^{H} = \left\{ {\begin{array}{*{20}l} {x_{n}^{m} + f_{1} \left( {u_{1} Xb_{best} - u_{2} X_{k}^{m} } \right)} \hfill & {} \hfill  \\ {\quad \quad  +  f_{2} \rho_{1} \left( {u_{3} \left( {X2_{n}^{m} - X1_{n}^{m} } \right)} \right) + u_{2} \left( {X_{r1}^{m} - X_{r2}^{m} } \right)/2} \hfill & {randN < 0.5\quad \quad (19{\text{a}})} \hfill \\ {Xb_{best} + f_{1} \left( {u_{1} Xb_{best} - u_{2} X_{k}^{m} } \right) } \hfill & {} \hfill  \\ {\quad \quad + f_{2} \rho_{1} \left( {\$ {\text{u}}_{{3}} \$ \left( {{\text{X2}}_{{\text{n}}}^{{\text{m}}} {\text{ - X1}}_{{\text{n}}}^{{\text{m}}} } \right)} \right) + u_{2} \left( {X_{r1}^{m} - X_{r2}^{m} } \right)/2} \hfill & {otherwise\quad \quad \quad (19{\text{b}})} \hfill \\ \end{array} } \right. \hfill \\ {\mathbf{End}} \hfill \\ \end{gathered} $$

where, $f_{1}$ and $f_{2}$ are uniformly distributed random values in [-1, 1], $P_{r}$ denotes a probability number equal to 0.5. $u1$, $u2$, and $u3$ are random numbers obtained from the following equations:

20 $$ u1 = \left\{ {\begin{array}{*{20}l} {2*randN} \hfill & {\mu_{1} < 0.5} \hfill \\ 1 \hfill & {otherelse} \hfill \\ \end{array} } \right. $$

21 $$ u2 = \left\{ {\begin{array}{*{20}l} {randN} \hfill & {\mu_{1} < 0.5} \hfill \\ 1 \hfill & {otherelse} \hfill \\ \end{array} } \right. $$

22 $$ u3 = \left\{ {\begin{array}{*{20}l} {randN} \hfill & {\mu_{1} < 0.5} \hfill \\ 1 \hfill & {otherelse} \hfill \\ \end{array} } \right. $$

where $randN$ is a random value between zero and one. $\mu_{1}$ is between 0 and 1. We can simplify the equations of $u1$, $u2$, and $u3$ in the following mathematical representation:

23 $$ u_{1} = L_{1} \times 2 \times randN + (1 - L_{1} ) $$

24 $$ u_{2} = L_{1} \times randN + \left( {1 - L_{1} } \right) $$

25 $$ u_{3} = L_{1} \times randN + \left( {1 - L_{1} } \right) $$

where $L_{1}$ is a parameter with a value of 0 or 1. (L1 = 1 if $\mu_{1} < 0.5$, and 0 otherwise).

The following scheme is presented to obtain the solution in Eq. (19).

26 $$ X_{k}^{m} = \left\{ {\begin{array}{*{20}l} {x_{{\text{randN }}} } \hfill & {{\text{ if }}\mu_{2} < 0.5} \hfill \\ {x_{p}^{m} } \hfill & {\text{ otherwise }} \hfill \\ \end{array} } \right. $$

where $x_{{\text{randN }}}$ is a new solution that can be calculated as shown in Eq. (27), $x_{p}^{m}$ is a random solution selected from the population $(p \in [1,2, \ldots N]$), $\mu_{2}$ is a random number in the range of [0,1].

27 $$ x_{{\text{randN }}} = lb + {\text{randN}} (0,1) \times \left( {ub - lb} \right) $$

Moreover, $\rho_{1}$ is used to balance the exploration and exploitation phases. It is defined by:

28 $$ \rho_{1} = 2 \times {\text{rand}} \times \alpha - \alpha $$

29 $$ \alpha = \left| {\beta \times \sin \left( {\frac{3\pi }{2} + \sin \left( {\beta \times \frac{3\pi }{2}} \right)} \right)} \right| $$

30 $$ \beta = \beta_{\min } + \left( {\beta_{\max } - \beta_{\min } } \right) \times \left( {1 - \left( {\frac{t}{{t_{max} }}} \right)^{3} } \right)^{2} $$

where $\beta_{\min }$ and $\beta_{\max }$ are equal to 0.2 and 1.2, respectively, t is the current step and $t_{max}$ is the highest number of steps—changes according to the sine function to balance the exploration and exploitation phases $\alpha$.

Equation (26) can be simplified using Eq. (31):

31 $$ X_{k}^{m} = w_{2} \times x_{p}^{m} + \left( {1 - w_{2} } \right) \times x_{{\text{rand }}} $$

where $w_{2}$ is a parameter with a value of 0 or 1. If the parameter $\mu_{1}$ is less than 0.5, the value of L1 is 1; otherwise, it is 0.

## The proposed mAHA algorithm

In this section, we present a detailed explanation of the proposed mAHA optimization algorithm, which aims to improve the searchability of the AHA and eliminate its weaknesses in solving complex real-world problems. The mAHA algorithm consists of two effective schemes: the LEO and the OBL. To enhance the performance of the original AHA, the OBL strategy is utilized in the initialization phase. After that, the steps of the original AHA are carried out as usual, and the LEO is used to improve its performance further.

### Drawbacks of the basic AHA algorithm

The basic AHA algorithm is based on hummingbirds’ foraging behavior, including guided foraging, territorial foraging, and migrating foraging. The algorithm generates diverse solutions by randomly applying these foraging strategies. However, in some optimization issues, the AHA algorithm can get trapped in sub-regions, resulting in improper exploration–exploitation balance, particularly in complex and high-dimensional problems. Since each solution updates its position based on the previous one, the algorithm’s convergence rate is reduced, and it cannot effectively cover search space solutions, leading to premature convergence. Therefore, we have developed a new version of the AHA algorithm to address these limitations. The LEO prevents getting trapped in sub-regions, solving premature convergence by updating solutions using a robust strategy and randomly selecting a solution over the search space. Furthermore, we utilize the OBL to improve the algorithm’s search efficiency, considering the No Free Lunch (NFL) theory that no superior optimization algorithm works well for all optimization problems.

### Initialization of the proposed mAHA

The initialization process of the mAHA algorithm follows the AHA algorithm and starts by proposing an initial population of (N) search agents. Each search agent is limited by upper and lower boundaries (ub_a_ and lb_a_) in the search space, as described in Eq. (1). The mAHA algorithm aims to enhance the diversity of the search process, which is achieved through the utilization of the OBL strategy during the initialization phase. This helps to improve the search operation, as demonstrated in Eq. (32).

32 $$ Opp_{s} = lb_{a} + ub_{a} - y_{b} ,b \in 1,2,...,N_{n} $$

where $Opp_{s}$ is a vector produced by applying OBL. $lb_{a}$, and $ub_{a}$ are lower and upper bounds of the $a^{th}$ component of Y, respectively. After that, the visit table of food sources is initialized, as shown in Eq. (2).

### Fitness evaluation of the proposed mAHA

It is compulsory to assess the solutions in each iteration to estimate the proposed solutions and to improve the new proposed solutions in the next step. In each iteration, the population of hummingbird positions is evaluated to get the fitness value of each solution $f(x)$. The best solution is determined $Xb_{best}$ and is used in updating the position rule.

### Updating process of the proposed mAHA

The AHA update steps are divided into two processes, as described in Eq. (33). The first process is divided into three steps, as illustrated in subsection "AHA mathematical model"; guided foraging, territorial foraging, and migration foraging. There is a probability of 50% to perform either guided foraging or territorial foraging. In the guided foraging, each search agent is updated using equations presented in Eqs. (6)–(9). While in the territorial foraging phase. The search agents are updated using equations presented in Eqs. (10)–(12). The migration foraging is applied every 2n iteration as illustrated in Eqs. (13) and (14). The second process works on the received solutions from previous process and target to significantly change these solutions using the LEO operator (described in details in subsection "Local escaping operator (LEO)"). Depending on specific criteria ($randN < p_{r}$), the final process is applied. Where $randN$ is a random value between zero and one, and $P_{r}$ is a probability value for performing the second process.

33 $$ Xb(t + 1) = \left\{ {\begin{array}{*{20}l} {X_{LEO}^{H} \quad using\:LEO\:operator} \hfill & {IfrandN < p_{r} } \hfill \\ {Xb_{best} \quad using\:the\:AHA\:updating\:process} \hfill & {otherwise} \hfill \\ \end{array} } \right. $$

### Termination criteria of the proposed mAHA

**Figure 1:**
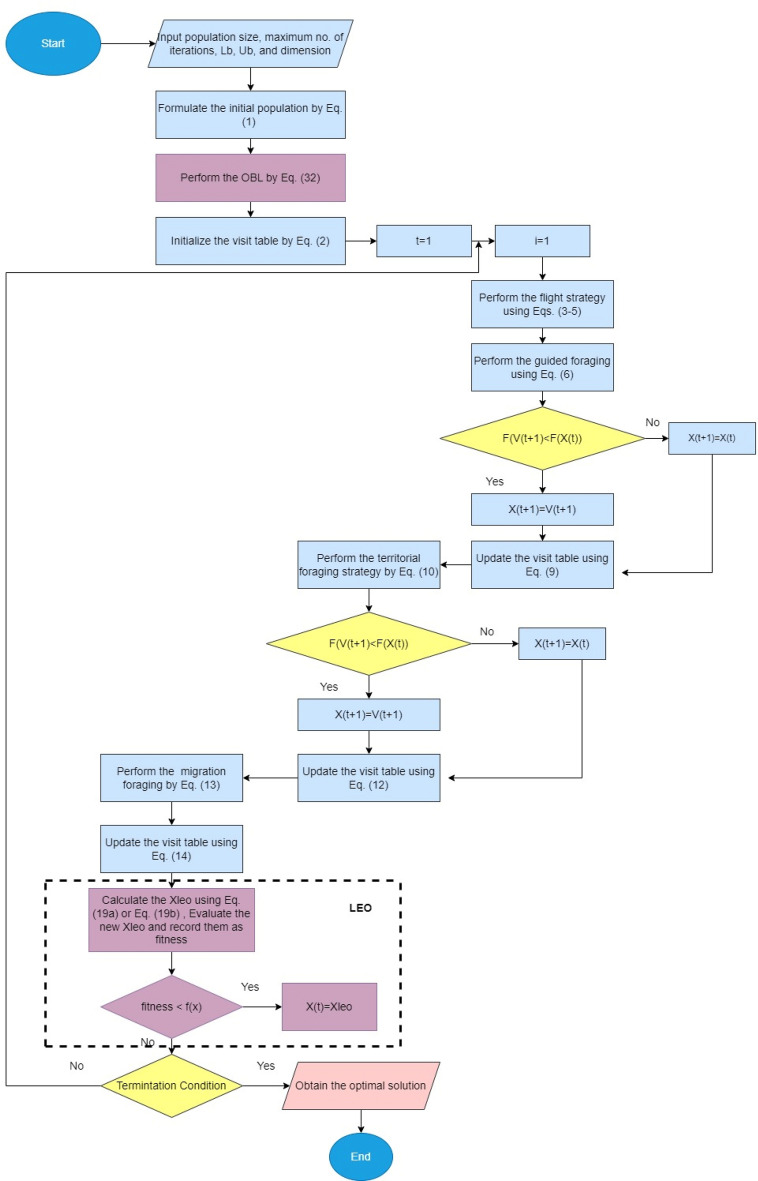
Flowchart of mAHA algorithm.

The proposed mAHA optimization process is repeated until the stopping criteria is met. The pseudo-code of the proposed mAHA algorithm is provided in Algorithm 2 and the flowchart is presented in Fig. 1.

**Algorithm 2:**
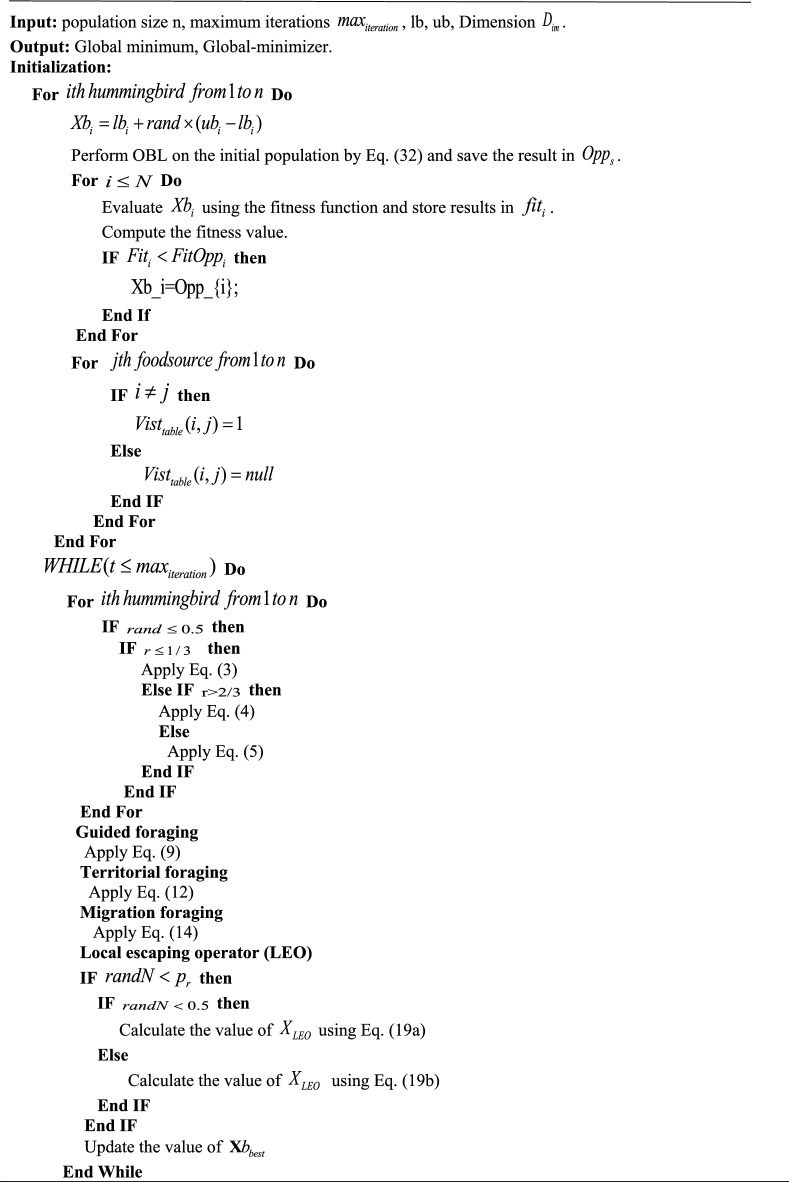
Pseudo-code of the proposed mAHA algorithm.

## Application of mAHA: optimal power flow and generation capacity

### Formulizing OPF mathematically

Optimizing the power system's control variables allows the objective function of the OPF issue can be maximized to meet specific objectives. To achieve this, different equality constraints and inequality constraints must be satisfied at the same time. This optimization problem can be put into mathematical terms by explaining it in the following way:

34 $$min F(x, u)$$

Conditional on:

$${g}_{j}\left(x,u\right)=0 j=\mathrm{1,2},\dots ,m$$

$${h}_{j}\left(x,u\right)\le 0 j=\mathrm{1,2},\dots ,p$$

where function F is the representation of the objective function. The vector $x$ contains the dependent variables (state variables), while the vector $u$ contains the independent variables (control variables). Additionally, ${g}_{j}$ and ${h}_{j}$ respectively represent the equality and inequality requirements. The variables $m$ and $p$ indicate the number of equality and inequality constraints.

The following are the state variables ($x$) in a power system:

35 $$x=\left[{P}_{G1 }{,V}_{L1 }{\dots {V}_{L,NPQ },Q}_{G,1}{\dots {Q}_{G,NG} ,S}_{TL,1 }{\dots S}_{TL,NTL}\right]$$

where the power of the slack bus is denoted by ${P}_{G1}$, and ${V}_{L}$ denotes the load bus voltage, the reactive output power for the generator is denoted by ${Q}_{G}$, the apparent power flow of the transmission line is denoted by ${S}_{TL}$, the number of load buses is denoted by $NPQ$, the number of generation buses is denoted by $NG$, and $NTL$ in the power system denotes the number of transmission lines.

In a power system, the control variables ($u$) are as follows:

36 $$u=\left[{P}_{G,2}\dots {P}_{G,NG}{,V}_{G,1 }{\dots {V}_{G,NG },Q}_{C,1}{\dots {Q}_{C,NC} ,T}_{1 }{\dots T}_{NT}\right]$$

where the generator output power is indicated by ${P}_{G}$, generation bus voltage is indicated by ${V}_{G}$, injected shunt compensator reactive power is indicated by ${Q}_{C}$, transformer tap settings are indicated by $T$, $NT$ indicates transformers and shunt compensator units are indicated by $NC$. It is important to note that these variables are relevant in this context.

### Objective functions

It is necessary to define an objective function to select the optimal solution. Several objectives are evaluated in the OPF, considering constraints within the system. In addition, the OPF determines the system’s optimal control variables and objectives. Techno-economic advantages are associated with the most efficient OPF solution. These are sometimes called OPF objectives. As a result of these objectives, fuel costs will be reduced, resulting in a reduction in annual operating costs as well as technological benefits, such as^3^: Minimization of active power losses, Minimization of reactive power losses, Improvement in system reliability and power quality; Deviation of voltage; and stabilization of voltage.

#### Single objective functions

The objective function described above is one of the most frequently used objective functions within the field of statistics, and it can be performed as follows^56^:

##### Basic fuel costs minimization objective

The primary goal of the OPF problem is to minimize the total fuel costs, which is achieved through an objective function. For each generator, the objective function can be expressed as a quadratic polynomial function, given by:

37 $${F}_{1}=\sum_{i=1}^{NG}{F}_{i}\left({P}_{Gi}\right)=\sum_{i=1}^{NPV}({a}_{i}+{b}_{i}{P}_{Gi}+{c}_{i}{{P}^{2}}_{Gi}) \frac{\mathrm{\$}}{h}$$

where, ${F}_{i}$ is the $i$ th generator fuel cost. ${a}_{i}$, ${b}_{i}$, and ${c}_{i}$ are the cost coefficients for $i$th generator.

##### Generation emission minimization objective

It is beneficial to decrease the quantity of gas released by thermal power plants to decrease pollution. The goal for regulating gas emissions can be described as follows:

38 $${F}_{2}=\sum_{i=1}^{NG}({\gamma }_{i}{{P}^{2}}_{Gi}+{\beta }_{i}{P}_{Gi}+{\alpha }_{i}{+{\upzeta }_{i} exp(\lambda }_{i}{P}_{Gi})$$

where, ${\gamma }_{i}$, ${\beta }_{i}$, ${\alpha }_{i}$,${\upzeta }_{i}$, and ${\lambda }_{i}$ are the $i$ th generator’s emission coefficients.

##### Active power losses minimization objective

The intended goal is to reduce the actual power loss, and this can be expressed in the following manner:

39 $${F}_{3}=\sum_{i=1}^{NTL}{G}_{ij}({{V}^{2}}_{i}+{{V}^{2}}_{j}-2 {V}_{i}{V}_{j}\mathrm{cos}{\delta }_{ij}) \mathrm{MW}$$

where, ${G}_{ij}$ is the transmission conductance, NTL is the transmission lines number, and ${\delta }_{ij}$ is the voltages phase difference.

##### Voltage deviation

Using this objective function, minimizing the deviation of voltages on the load nodes from a predetermined voltage is possible. The following formula can describe this:

40 $${F}_{4}=VD=\sum_{i=1}^{NPQ}\left|{V}_{i}-1\right|$$

#### Multi-objective functions

When dealing with a multi-objective issue, the main aim is to optimize various objectives that are independent of each other, and this is defined in the following equation:

41 $$Min F\left(x,u\right)=\left[{F}_{1}\left(x,u\right),{F}_{2}\left(x,u\right),\dots ,{F}_{i}\left(x,u\right)\right]$$

where $i$ is the number of the objective function, the optimization with the weighting factors as follows can be used to solve multi-objective functions:

42 $$Min {F}_{i}=\sum_{i=1}^{4}{F}_{i}\left(x,u\right)$$

43 $${F}_{i}\left(x,u\right)={F}_{1}+{w}_{1}{F}_{2}+{w}_{2}{F}_{3}+{w}_{3}{F}_{4}$$

44 $$ \begin{aligned}{F}_{i}\left(x,u\right)&=\sum_{i=1}^{NG}\left({a}_{i}+{b}_{i}{P}_{Gi}+{c}_{i}{{P}^{2}}_{Gi}\right)+{w}_{1}\sum_{i=1}^{NG}({\gamma }_{i}{{P}^{2}}_{Gi}+{\beta }_{i}{P}_{Gi}+{\alpha }_{i}{+{\upzeta }_{i} exp(\lambda }_{i}{P}_{Gi})\\ &\quad+{w}_{2}\sum_{i=1}^{NTL}{G}_{ij}({{V}^{2}}_{i}+{{V}^{2}}_{j}-2 {V}_{i}{V}_{j}\mathrm{cos}{\delta }_{ij})+{w}_{3}\sum_{i=1}^{NPQ}\left|{V}_{i}-1\right|\end{aligned} $$

where ${w}_{11}$, ${w}_{2}$ and ${w}_{3}$ are weight factors chosen based on the relative importance of one goal to another. Suitable weighting factors are selected by the user. In this paper, the values of the weight factors are chosen for each case as mentioned below:

**Table:** 

Case no.	Description	Objective function	Wight factors	Network	Control variable no
1	Minimization of fuel cost	${F}_{1}=\sum_{i=1}^{NPV}({a}_{i}+{b}_{i}{P}_{Gi}+{c}_{i}{{P}^{2}}_{Gi})$	-	Standard IEEE 30 & 118 bus	24/128
2	Minimization of active power losses	${F}_{3}=\sum_{i=1}^{NTL}{G}_{ij}({{V}^{2}}_{i}+{{V}^{2}}_{j}-2 {V}_{i}{V}_{j}\mathrm{cos}{\delta }_{ij})$	-	Standard IEEE 30 & 118 bus	24/128
3	Minimization of total voltage deviation	${F}_{4}=\sum_{i=1}^{NPQ}\left|{V}_{i}-1\right|$	-	Standard IEEE 30 & 118 bus	24/128
4	Minimization of fuel cost and power losses	${F}_{i}\left(x,u\right)={F}_{1}+{w}_{1}{F}_{3}$	${w}_{1}=20$	Standard IEEE 30	24
5	Minimization of fuel cost and total voltage deviation	${F}_{i}\left(x,u\right)={F}_{1}+{w}_{1}{F}_{4}$	${w}_{1}=200$	Standard IEEE 30	24
6	Minimization of fuel cost and power loss with emission	${F}_{i}\left(x,u\right)={F}_{1}+{w}_{1}{F}_{2}+{w}_{2}{F}_{3}$	${w}_{1}=0.0021, {w}_{2}=20$	Standard IEEE 30	24
7	Minimization of multi-objective function (voltage-level deviation, operational cost, and transmission power loss) without emission	${F}_{i}\left(x,u\right)={F}_{1}+{w}_{1}{F}_{3}+{w}_{2}{F}_{4}$	${w}_{1}=200, {w}_{2}=100$	Standard IEEE 30 & 118 bus	24/128
8	Minimization of multi-objective function (voltage-level deviation, operational cost, and transmission power loss) with emission	${F}_{i}\left(x,u\right)={F}_{1}+{w}_{1}{F}_{2}+{w}_{2}{F}_{3}+{w}_{3}{F}_{4}$	${w}_{1}=0.0065, {w}_{2}=200, {w}_{3}=100$	Standard IEEE 30	24
9	Optimal allocation for renewable energy sources for minimizing fuel cost	${F}_{1}=\sum_{i=1}^{NPV}({a}_{i}+{b}_{i}{P}_{Gi}+{c}_{i}{{P}^{2}}_{Gi})$	-	Standard IEEE 30	3
10	Minimization of the fuel cost with the penetration of RES	${F}_{1}=\sum_{i=1}^{NPV}({a}_{i}+{b}_{i}{P}_{Gi}+{c}_{i}{{P}^{2}}_{Gi})$	-	Modfied IEEE 30	24
11	Minimization of the fuel cost simultaneously with the penetration of RES	${F}_{1}=\sum_{i=1}^{NPV}({a}_{i}+{b}_{i}{P}_{Gi}+{c}_{i}{{P}^{2}}_{Gi})$	-	Standard IEEE 30	27

### System constraints

There are already many constraints in the system that can be classified as follows:

#### The equality constraints

The equality constraints for the balanced load flow equations are as follows:

45 $${P}_{Gi}-{P}_{Di}=\left|{V}_{i}\right|\sum_{j=1}^{NB}\left|{V}_{j}\right|({G}_{ij}\mathrm{cos}{\delta }_{ij}+{B}_{ij}sin{\delta }_{ij})$$

46 $${Q}_{Gi}-{Q}_{Di}=\left|{V}_{i}\right|\sum_{j=1}^{NB}\left|{V}_{j}\right|({G}_{ij}\mathrm{cos}{\delta }_{ij}+{B}_{ij}sin{\delta }_{ij})$$

where ${P}_{Gi}$ and ${Q}_{Gi}$ are the active power and reactive power generated respectively at bus $i$. The active and reactive demand of the load at bus $i$ are represented by ${P}_{Di}$ and ${Q}_{Di}$, respectively.${G}_{ij}$ and ${B}_{ij}$ represent conductance and susceptibility among buses $i$ and $j$, respectively.

#### Inequality constraints

The classification of inequality constraints is as follows:

47 $$\mathrm{Active \,\,output \,\,power\,\, of \,\,generators}: {{P}_{Gi}}^{min}\le {P}_{Gi}\le {{P}_{Gi}}^{max} i=\mathrm{1,2},\dots ,NG$$

48 $$\mathrm{Voltages \,\,at \,\,generators \,\,buses}: {{V}_{Gi}}^{min}\le {V}_{Gi}\le {{V}_{Gi}}^{max} i=\mathrm{1,2},\dots ,NG$$

49 $$\mathrm{Reactive \,\,output \,\,power \,\,of \,\,generators}: {{Q}_{Gi}}^{min}\le {Q}_{Gi}\le {{Q}_{Gi}}^{max} i=\mathrm{1,2},\dots ,NG$$

50 $$\mathrm{Tap\,\, settings \,\,of\,\, transformer}: {{T}_{i}}^{min}\le {T}_{i}\le {{T}_{i}}^{max} i=\mathrm{1,2},\dots ,NT$$

51 $$\mathrm{Shunt \,\,VAR \,\,compensator}: {{Q}_{Ci}}^{min}\le {Q}_{Ci}\le {{Q}_{Ci}}^{max} i=\mathrm{1,2},\dots ,NC$$

52 $$\mathrm{Apparent \,\,power \,\,flows\,\, in\,\,transmission\,\, lines}: {S}_{Li}\le {{S}_{Li}}^{min} i=\mathrm{1,2},\dots ,NTL$$

53 $$\mathrm{Magnitude \,\,of \,\,load \,\,buses\,\, voltage}: {{V}_{Li}}^{min}\le {V}_{Li}\le {{V}_{Li}}^{max} i=\mathrm{1,2},\dots ,NPQ$$

The incorporation of dependent control variables can be achieved seamlessly in an optimization solution by utilizing the quadratic penalty formulation of the objective function. In this paper, the optimization problem can be rewritten based on the penalty functions as follows:

54 $${F}_{g}\left(x,u\right)={F}_{i}\left(x,u\right)+{K}_{G}{(\Delta {P}_{G1})}^{2}+{K}_{Q}\sum_{i=1}^{NPV}{\left(\Delta {Q}_{Gi}\right)}^{2}+{K}_{V}\sum_{i=1}^{NPQ}{(\Delta {V}_{Li})}^{2}+{K}_{S}\sum_{i=1}^{NTL}{\left(\Delta {S}_{Li}\right)}^{2}$$

where ${K}_{G}$, ${K}_{Q}$, ${K}_{V}$, and ${K}_{S}$ are penalty factors with large positive values, also $\Delta {P}_{G1}$, $\Delta {Q}_{Gi}$, $\Delta {V}_{Li}$, and $\Delta {S}_{Li}$ are penalty conditions that can be stated as follows:

55 $$\Delta {P}_{G1}= \left\{\begin{array}{c}\left({P}_{G1}-{{P}_{G1}}^{max}\right) {P}_{G1}>{{P}_{G1}}^{max}\\ \left({P}_{G1}-{{P}_{G1}}^{min}\right){ P}_{G1}<{{P}_{G1}}^{min}\\ 0 {{P}_{G1}}^{min}<{P}_{G1}<{{P}_{G1}}^{max}\end{array}\right.$$

56 $$\Delta {Q}_{Gi}= \left\{\begin{array}{c}\left({Q}_{Gi}-{{Q}_{Gi}}^{max}\right) {Q}_{Gi}>{{Q}_{Gi}}^{max}\\ \left({Q}_{Gi}-{{Q}_{Gi}}^{min}\right){ Q}_{Gi}<{{Q}_{Gi}}^{min}\\ 0 {{Q}_{Gi}}^{min}<{Q}_{Gi}<{{Q}_{Gi}}^{max}\end{array}\right.$$

57 $$\Delta {V}_{Li}= \left\{\begin{array}{c}\left({V}_{Li}-{{V}_{Li}}^{max}\right) {V}_{Li}>{{V}_{Li}}^{max}\\ \left({V}_{Li}-{{V}_{Li}}^{min}\right){ V}_{Li}<{{V}_{Li}}^{min}\\ 0 {{V}_{Li}}^{min}<{V}_{Li}<{{V}_{Li}}^{max}\end{array}\right.$$

58 $$\Delta {S}_{Li}= \left\{\begin{array}{c}\left({S}_{Li}-{{S}_{Li}}^{max}\right) {S}_{Li}>{{S}_{Li}}^{max}\\ ({S}_{Li}-{{S}_{Li}}^{min}){ S}_{Li}<{{S}_{Li}}^{min}\\ 0 {{S}_{Li}}^{min}<{S}_{Li}<{{S}_{Li}}^{max}\end{array}\right.$$

## Evaluated results and discussion

This section describes two experiments to assess mAHA performance using different metrics. The first experiment used mAHA on 10 problems taken from the CEC2020 benchmark functions^57^, while the second experiment focused on testing mAHA’s effectiveness in solving the OPF problem. The OPF problem was tested on the IEEE 30-bus system.

### Experimental Series 1: global optimization with CEC’2020 test-suite

Several benchmark function challenges presented by the CEC’2020 illustrate how well the mAHA performs. Several well-known metaheuristic methodologies are compared with this mAHA technique to evaluate its effectiveness: the WOA^58^, the SCA^59^, the TSA^60^, the SMA^61^, the HHO, the RUN^63^, and the basic AHA algorithm^52^.

#### Definition of CEC’20 benchmark functions

**Figure 2:**
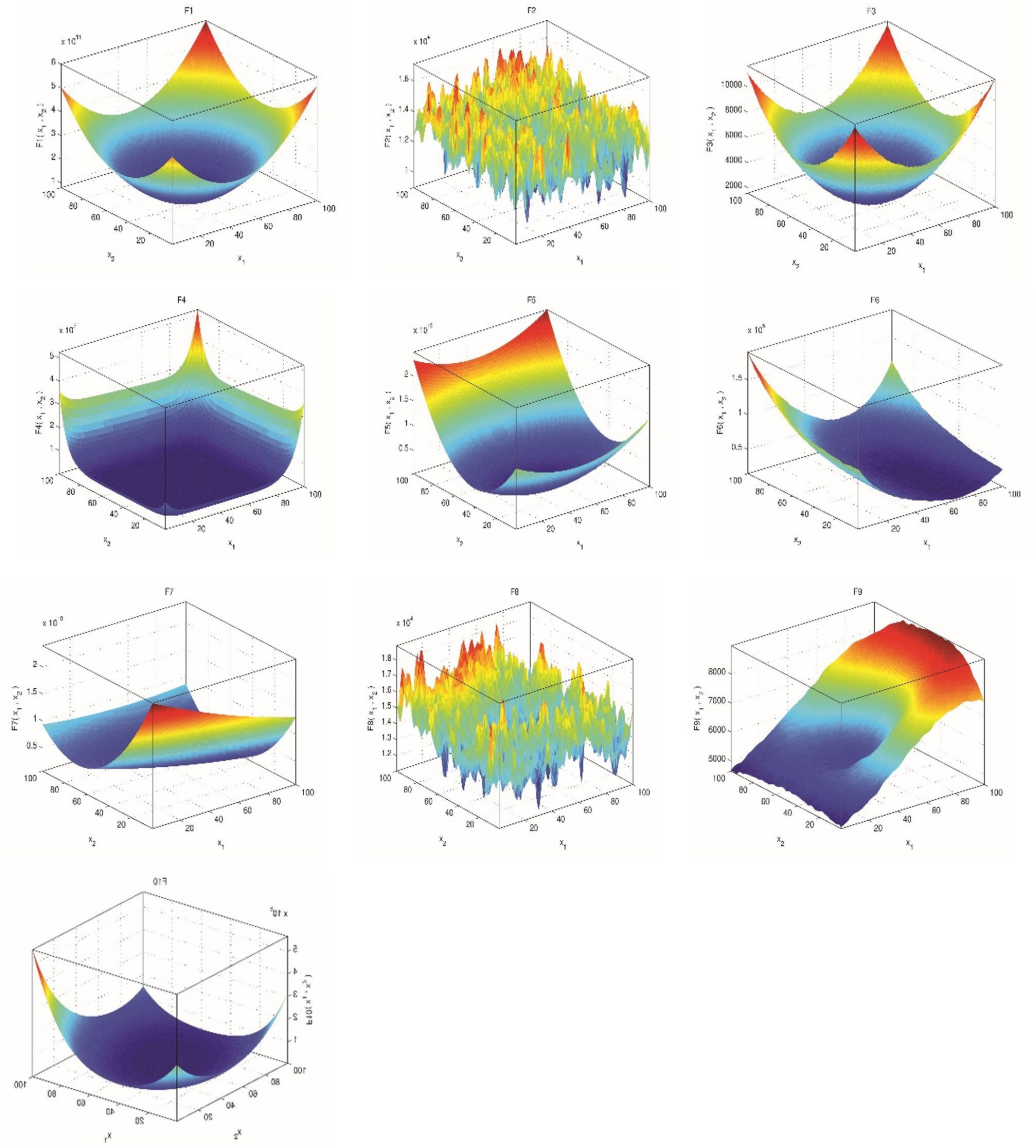
The 3D visualization of the CEC'2020 functions.

In order to evaluate the proposed method’s performance, IEEE CEC’2020 benchmarks^64^ were used as test problems to estimate its performance. As part of the benchmarking process, 10 different test functions have been included to cover uni-modal, multi-modal, hybrid, and composition test functions. Here are the benchmark test characteristics and mathematical equations, with ‘Fi*’ denoting the optimal global value. Figure 2, three-dimensional views of CEC’2020 functions (Table 2).

**Table 2:** Describing the CEC’2020 test-suite.

No	Function specification	Fi*
Uni-modal function		
F1	Shifted and rotated Bent Cigar function	100
Multi-modal shifted and rotated functions		
F2	Shifted and rotated schwefel's function	1100
F3	Shifted and Rotated Lunacek bi-Rastrigin function	700
F4	Expanded Rosenbrock’s plus Griewangk’s function	1900
Hybrid functions		
F5	N = 3	1700
F6	N = 4	1600
F7	N = 5	2100
Composition functions		
F8	N = 3	2200
F9	N = 4	2400
F10	N = 5	2500

#### Parameter settings

To compare the mAHA algorithm and other algorithms, 30 runs were conducted. All considered problems had a fixed number of function evaluations (Fes) set at 30,000. Table 3 displays the parameter settings for each algorithm, as reported in the original literature. Qualitative and quantitative metrics were utilized to evaluate the algorithms’ effectiveness.

**Table 3:** Setting of parameters for the compared algorithms.

Methodology	Settings
Common settings	Size of population: N = 30 Maximum function evaluation: M AX FEs = 30,000 Dimension of problem Dim = 10 Runs number 30
WOA	α reduces from 2 to 0 (Default)
SCA	A = 2 (Default)
TSA	Pmin = 1, Pmax = 4 (Default)
SMA	z = 0.03 (Default)
HHO	E0 = 1.67, E1 = 1, beta = 1.5
RUN	a = 20 and b = 12 (Default)
AHA	(Default Values)
mAHA	(Default Values)

#### Performance criteria

The proposed algorithm's efficiency in finding the best solutions is evaluated against comparison algorithms using a collection of performance metrics in this paper. The definitions for these metrics are outlined below:

**Statistical mean:** This metric determines the fitness value that is situated in the center, and it is computed using the following equation:

59 $$ Mean = \frac{1}{{R_{n} }}\sum\limits_{j = 1}^{{R_{n} }} F itt_{b}^{i} $$

**The worst value:** This metric is utilized to compute the highest fitness value that the algorithm can achieve, and it is defined as:

60 $$ WORST = \max_{{1 \le j \le R_{n} }} Fitt_{b}^{i} $$

**The best value:** This metric computes the minimum fitness value, and it can be defined as follows:

61 $$ BEST = \min_{{1 \le j \le R_{n} }} Fitt_{b}^{i} $$

**Standard deviation (STD):** The STD is calculated by the following equation:

62 $$ STD = \sqrt {\frac{1}{{R_{n} - 1}}\sum\limits_{j = 1}^{{R_{n} }} {(Fitt_{b}^{i} - Mean)^{2} } } $$

where $R_{n}$ represents the total number of runs.

#### Statistical investigation on CEC’2020 test-suite

The proposed mAHA algorithm is compared to WOA, SCA, TSA, SMA, HHO, RUN, and AHA on the CEC’2020 test suite, and statistical results are obtained. A measure of the algorithm’s performance is assessed by calculating the mean value and standard deviation of the best-so-far solutions obtained within each run. Based on the dimension ‘Dim = 10’ of the CEC’2020 test suite, Table 4 displays mean, standard deviation, best, and worst values. Boldfaced values highlight the most appropriate values.

**Table 4:** Fitness values generated by competitor algorithms over 30 experiments conducted for CEC'2020.

Function	Metric	WOA	SCA	TSA	SMA	HHO	RUN	AHA	mAHA
F1	Mean	6.473E+06	8.199E+08	2.565E+09	6.759E+03	5.652E+05	3.830E+03	1.829E+03	**1.000E+02**
	Std	8.747E+06	3.019E+08	2.068E+09	4.193E+03	5.827E+05	2.234E+03	1.629E+03	**1.828E−02**
	Best	6.949E+05	2.944E+08	1.078E+07	2.415E+02	9.659E+04	1.572E+02	1.159E+02	**1.000E+02**
	Worst	4.477E+07	1.481E+09	7.693E+09	1.271E+04	3.162E+06	9.526E+03	6.359E+03	**1.001E+02**
F2	Mean	2.175E+03	2.370E+03	2.077E+03	1.594E+03	2.080E+03	1.693E+03	1.471E+03	**1.336E+03**
	Std	2.943E+02	1.937E+02	3.455E+02	2.244E+02	2.491E+02	2.079E+02	1.985E+02	**1.548E+02**
	Best	1.615E+03	1.927E+03	1.414E+03	1.226E+03	1.605E+03	1.324E+03	1.115E+03	**1.100E+03**
	Worst	2.750E+03	2.899E+03	2.863E+03	2.051E+03	2.698E+03	2.078E+03	1.936E+03	**1.699E+03**
F3	Mean	7.777E+02	7.766E+02	7.936E+02	7.284E+02	7.819E+02	7.609E+02	7.365E+02	**7.255E+02**
	Std	2.592E+01	**1.088E+01**	3.091E+01	8.521E+00	1.784E+01	1.656E+01	1.138E+01	8.629E+00
	Best	7.261E+02	7.532E+02	7.470E+02	7.176E+02	7.418E+02	7.208E+02	7.216E+02	**7.135E+02**
	Worst	8.416E+02	7.971E+02	8.598E+02	7.558E+02	8.185E+02	8.084E+02	7.717E+02	**7.505E+02**
F4	Mean	1.908E+03	**1.928E+03**	1.634E+04	**1.901E+03**	**1.908E+03**	**1.902E+03**	**1.901E+03**	**1.901E+03**
	Std	9.512E+00	1.970E+01	2.714E+04	5.555E−01	2.992E+00	1.465E+00	7.627E−01	**7.439E−01**
	Best	1.903E+03	1.909E+03	1.903E+03	1.901E+03	1.903E+03	**1.900E+03**	**1.900E+03**	**1.900E+03**
	Worst	1.955E+03	2.007E+03	1.252E+05	**1.903E+03**	1.913E+03	1.906E+03	1.904E+03	**1.903E+03**
F5	Mean	3.308E+05	4.694E+04	4.427E+05	7.437E+03	5.209E+04	4.211E+03	6.619E+03	**2.719E+03**
	Std	5.992E+05	6.608E+04	3.475E+05	5.520E+03	6.303E+04	**1.377E+03**	4.882E+03	1.507E+03
	Best	9.792E+03	1.029E+04	2.724E+03	1.854E+03	2.861E+03	2.302E+03	1.744E+03	**1.719E+03**
	Worst	2.614E+06	3.812E+05	9.381E+05	1.939E+04	2.084E+05	7.488E+03	2.291E+04	**8.325E+03**
F6	Mean	1.612E+03	1.603E+03	1.630E+03	**1.601E+03**	1.620E+03	**1.601E+03**	1.602E+03	**1.601E+03**
	Std	1.299E+01	2.398E+00	2.318E+01	3.053E−01	8.626E+00	2.714E−01	3.043E+00	**3.210E−01**
	Best	**1.601E+03**	**1.601E+03**	**1.601E+03**	**1.601E+03**	**1.601E+03**	**1.601E+03**	**1.601E+03**	**1.601E+03**
	Worst	1.660E+03	1.615E+03	1.667E+03	**1.602E+03**	1.632E+03	**1.602E+03**	1.618E+03	**1.602E+03**
F7	Mean	1.756E+05	1.368E+04	4.182E+04	6.657E+03	1.341E+04	4.414E+03	3.288E+03	**2.125E+03**
	Std	2.557E+05	7.169E+03	7.349E+04	6.060E+03	2.811E+04	3.051E+03	2.278E+03	**3.179E+01**
	Best	1.131E+04	4.463E+03	2.654E+03	2.241E+03	2.451E+03	2.144E+03	2.102E+03	**2.100E+03**
	Worst	9.505E+05	3.215E+04	2.038E+05	2.112E+04	1.573E+05	1.391E+04	9.947E+03	**2.235E+03**
F8	Mean	2.348E+03	2.392E+03	2.620E+03	2.412E+03	2.410E+03	2.305E+03	2.300E+03	**2.299E+03**
	Std	1.612E+02	4.104E+01	4.015E+02	3.440E+02	3.016E+02	1.645E+01	**1.187E+01**	1.670E+01
	Best	2.261E+03	2.298E+03	2.234E+03	2.228E+03	2.264E+03	2.222E+03	2.237E+03	**2.211E+03**
	Worst	3.199E+03	2.481E+03	4.127E+03	3.540E+03	3.562E+03	2.324E+03	**2.305E+03**	2.308E+03
F9	Mean	2.770E+03	2.773E+03	2.793E+03	2.739E+03	2.829E+03	2.748E+03	2.654E+03	**2.647E+03**
	Std	5.640E+01	6.138E+01	**1.038E+02**	6.552E+01	5.099E+01	8.740E+00	1.218E+02	1.224E+02
	Best	2.561E+03	2.545E+03	2.554E+03	**2.500E+03**	2.739E+03	2.734E+03	2.500E+03	**2.500E+03**
	Worst	2.829E+03	2.813E+03	2.906E+03	2.776E+03	2.936E+03	2.766E+03	**2.768E+03**	**2.768E+03**
F10	Mean	2.948E+03	2.980E+03	3.039E+03	2.936E+03	2.932E+03	**2.921E+03**	2.932E+03	2.930E+03
	Std	3.155E+01	2.636E+01	1.671E+02	3.068E+01	3.452E+01	2.464E+01	**2.180E+01**	2.205E+01
	Best	2.902E+03	2.941E+03	2.899E+03	**2.898E+03**	2.899E+03	**2.898E+03**	**2.898E+03**	**2.898E+03**
	Worst	3.030E+03	3.064E+03	3.648E+03	3.024E+03	3.028E+03	2.956E+03	2.951E+03	**2.947E+03**
Friedman mean rank		5.19	5.80	6.15	4.60	4.85	4.22	3.23	2.02
rank		6	7	8	4	5	3	2	1

As shown in Table 4, the results show that the mAHA technique reaches the optimum value with respect to the single-modal benchmark function F1 for the unimodal model. There is no doubt that mAHA has an advantage over the algorithms which are compared for multi-modal functions F2, F3, and F4 in terms of performance. Nevertheless, regarding the F4 function, the most accurate values can be obtained using mAHA, AHA, RUN, and SMA. In addition, the proposed mAHA technique performs better than any of the other methodologies regarding the hybrid F5, F6, and F7 test functions. For the composite functions F8, F9, and F10, the mAHA algorithm outperforms the other algorithms. The mAHA and AHA algorithms provide optimal F8 values. For test function F9, optimal results are achieved by the mAHA and SMA algorithms. In contrast, for the F10 test function, the mAHA, AHA, RUN, and SMA techniques achieve optimal values.

In terms of resolving the CEC’2020 benchmark functions, the statistical results indicate that the mAHA methodology performs better than any of the other methods. A comparison of the mean, the standard deviation, the best value, and the worst value can be made to reveal this. It is also noteworthy that, in the Friedman mean rank-sum test, the proposed mAHA algorithm achieved the top ranking in the Friedman algorithm test.

#### Boxplot behavior analysis

Boxplots are a valuable and effective tool for analyzing data visually and representing its empirical distribution. They are created by dividing the data into quartiles, with the highest and lowest whiskers representing the maximum and minimum values in the dataset. The box represents the lower and upper quartiles, providing insight into the data's spread and level of agreement. When the box is narrow, it indicates a high degree of symmetry in the data.

**Figure 3:**
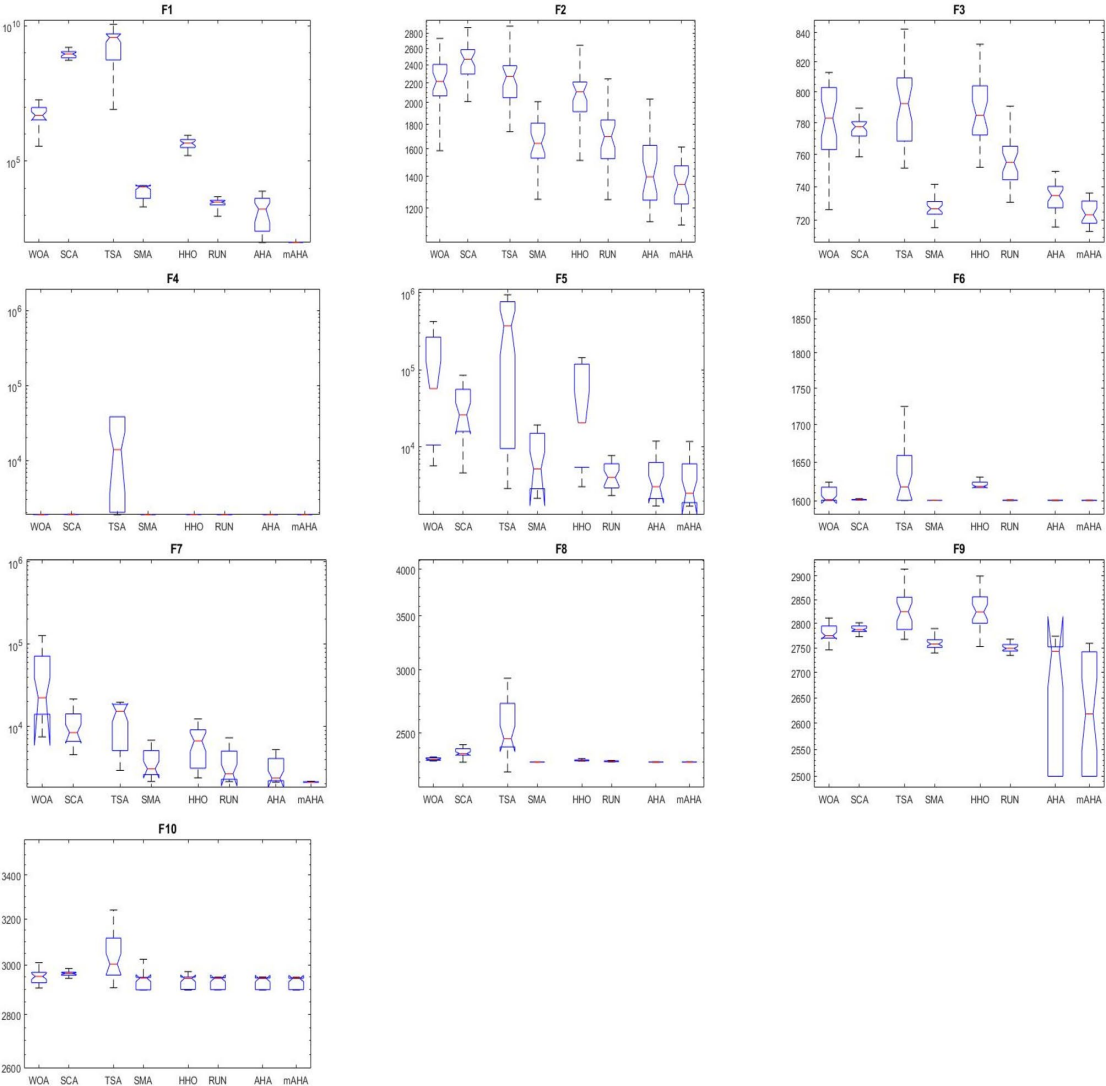
Boxplot curves of the proposed mAHA, as well as the other compared algorithms, were obtained over the CEC'2020 test suite with a Dim of 10.

Figure 3 shows the boxplot distribution for the CEC'20 test functions from F1 to F10 with a dimension of 10. The results of the introduced mAHA algorithm demonstrate narrower boxplots and minimum values compared to other algorithms for most test methods. These graphical results confirm the mAHA algorithm's consistency in finding optimal regions for the test problems.

#### Evaluation of convergence performance

**Figure 4:**
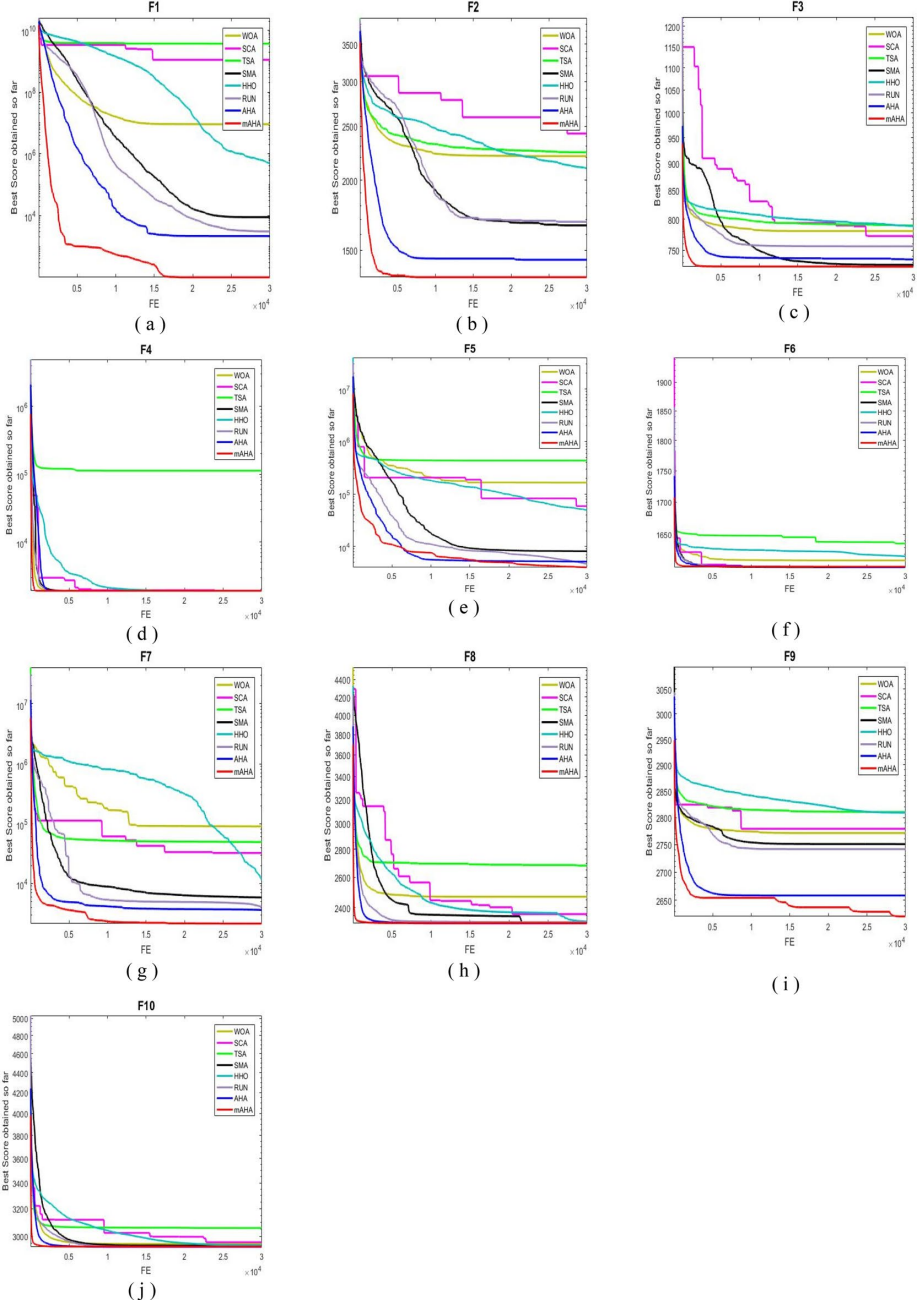
Convergence curves of mAHA and the other methodologies estimated on CEC’20 functions.

Algorithm convergence is discussed in this subsection. For CEC 2020 test problems for dimension 10, Fig. 3 compares WOA, SCA, TSA, SMA, HHO, RUN, and AHA to the developed mAHA. Figure 4a shows that the F1 function with a unimodal space exhibits convergence curves. It has been demonstrated that the proposed mAHA is superior to the original AHA and all other algorithms compared. It is evident in Fig. 3b–d that the developed mAHA algorithm displays a greater level of exploration than the standard OPA algorithm and the other algorithms that have been compared on the benchmark functions of F2–F4. Using the benchmark F5 function, the proposed mAHA and the original AHA have significant results, as illustrated in Fig. 3e–g. A significant performance improvement was also achieved by the mAHA for functions F6 and F7. Therefore, the mAHA is more effective at handling hybrid functions. It was demonstrated from the composition functions (F8, F9, and F10) in Figs. 3h–j that the proposed mAHA was able to solve problems involving complex spaces with comparable performance.

### Experimental series 2: applying mAHA for solving OPF problems

On the IEEE 30-bus test grid, the effectiveness of the mAHA methodology is evaluated to address the OPF issue. This section compares simulation results between those obtained by mAHA and those obtained by recent metaheuristic algorithms to solve OPF. An evaluation of mAHA's ability to minimize fuel costs, active power loss, total voltage deviation, and emissions is conducted for one-objective and multi-objective problems considering weight factors. Using the presented cases, it is possible to determine these weight factors.

mAHA's effectiveness is further demonstrated by comparing it to other algorithms. The test is conducted on a modified IEEE 30-bus grid to determine its effectiveness in optimizing RES allocation and minimizing fuel costs. Experimental tests are used to determine which parameters are appropriate for mAHA and other methods. Each algorithm is run 30 times on the test system with different parameters. A MATLAB 2021b platform is used to apply mAHA and other comparing techniques to solve the OPF issue. This is accomplished by using a PC with a 2.8GHz I7-8700 CPU and 16 GB of RAM.

#### IEEE 30-bus grid

**Figure 5:**
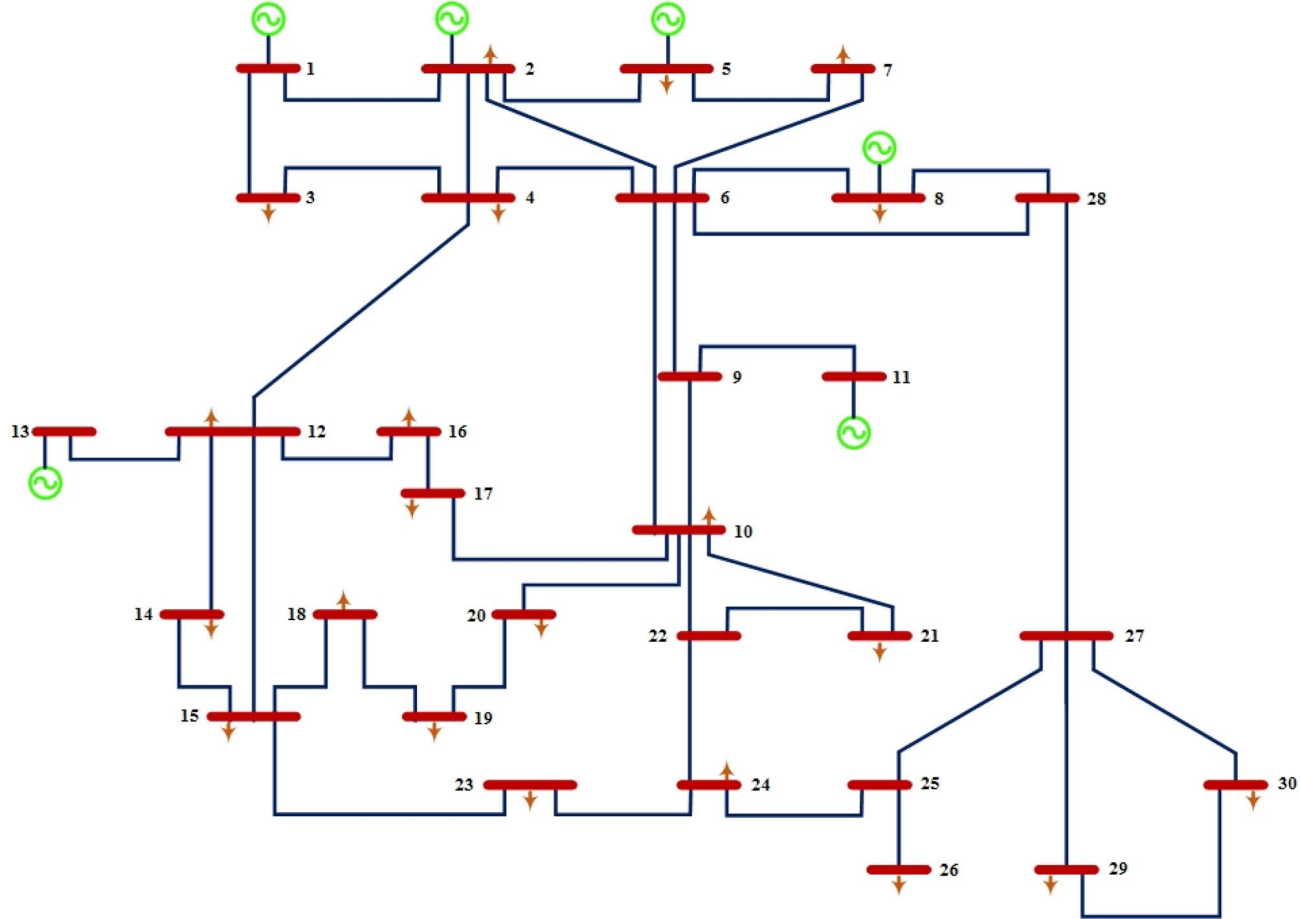
Standard IEEE-30 bus test system.

IEEE 30-bus grid has six generation power units, 41 lines, and 24 load buses^66^. Figure 5 shows node number 1 is a slack bus^66^. In terms of active power and reactive power, the total connected load has 2.834 pu of active and 1.262 pu of reactive power, respectively. A voltage magnitude of 0.95 Pu and 1.1 Pu is limited for the power-generating nodes, while a voltage magnitude of 0.95 Pu and 1.05 Pu is limited for the remaining load nodes. VAR compensator limits fluctuate between 0 and 0.05 pu, and tap-changing transformers can be adjusted between 0.9 and 1.1 pu.

##### Case 1: minimization of fuel cost

**Figure 6:**
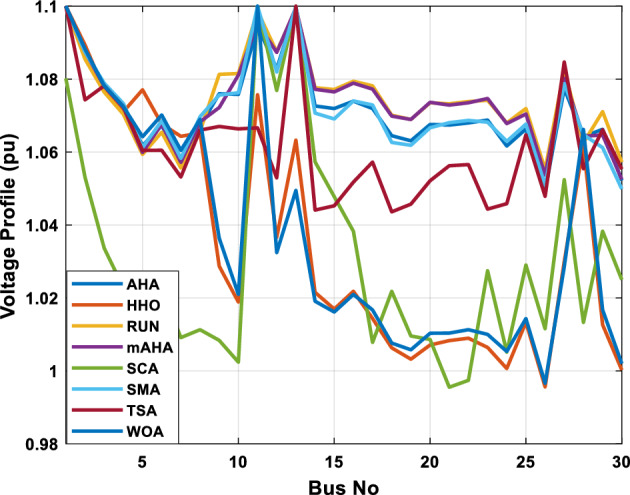
The voltage profile of the different techniques for case 1.

**Figure 7:**
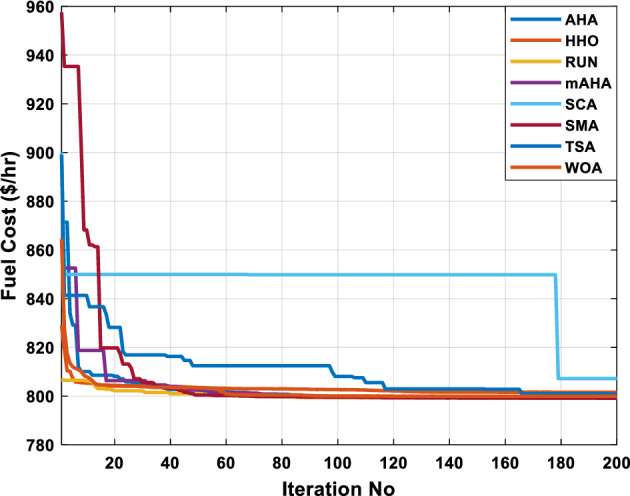
The convergence characteristics of compared methods for case 1.

A mAHA methodology is proposed for reducing fuel costs using only the IEEE 30-bus grid. According to Table 5, mAHA achieves optimal outcomes as opposed to other literature techniques, such as AHA, HHO, RUN, SCA, SMA, TSA, and WOA. The mAHA technique produces the lowest fuel cost of 799.135 $/h, outperforming other methodologies. The mAHA's voltage profile is also displayed in Fig. 6, ensuring that all nodes' voltages are within acceptable limits. As can be seen in Fig. 7, the convergence characteristics of the standard algorithm and other compared techniques are described in terms of minimizing fuel cost (over 200 iterations). According to this figure, the mAHA methodology exhibits a better convergence characteristic than other techniques, with the optimum value reached after 50 iterations; this means that the suggested technique exhibits faster convergence.

**Table 5:** Optimum control variables for IEEE 30-bus grid for minifying fuel cost.

Control variables	AHA	HHO	mAHA	RUN	SCA	SMA	TSA	WOA	CGSCE^50^
$${P}_{G1}$$ (MW)	176.906	178.999	177.1779	177.306	172.6857	176.7942	179.9009	175.5387	177.120
$${P}_{G2}$$ (MW)	48.051	48.558	48.7003	48.511	49.5774	48.3917	47.2028	47.5454	48.6931
$${P}_{G5}$$ (MW)	21.279	20.884	21.4732	21.220	25.9249	21.1671	21.0713	20.6348	21.3708
$${P}_{G8}$$ (MW)	21.719	13.578	21.0560	20.643	22.2514	21.2451	18.4862	21.4456	21.2720
$${P}_{G11}$$ (MW)	11.990	17.119	11.6398	11.817	10	12.4284	13.9516	14.6936	11.9708
$${P}_{G13}$$ (MW)	12.066	13.407	12	12.564	13.0287	12	12	12.1966	12.0011
$${V}_{1}$$ (pu)	1.0998	1.1	1.1	1.0997	1.08018	1.1	1.1	1.1	1.0848
$${V}_{2}$$ (pu)	1.0876	1.0893	1.08753	1.0854	1.05307	1.08743	1.07431	1.08822	1.0653
$${V}_{5}$$ (pu)	1.0617	1.0770	1.05989	1.0593	1.00887	1.06122	1.06045	1.06419	1.0338
$${V}_{8}$$ (pu)	1.0670	1.0652	1.06828	1.0656	1.01128	1.06968	1.06601	1.06895	1.0384
$${V}_{11}$$ (pu)	1.0945	1.0757	1.09523	1.0986	1.1	1.1	1.06663	1.1	1.0993
$${V}_{13}$$ (pu)	1.0994	1.0632	1.09950	1.0993	1.1	1.09995	1.1	1.04947	1.0462
$${T}_{11}$$ (6–9)	1.0073	1.0651	1.03251	1.0002	1.1	1.01595	0.99058	1.06601	1.0377
$${T}_{12}$$ (6–10)	0.9569	1.0104	0.91963	0.9523	1.1	0.94796	0.9	1.03469	0.9539
$${T}_{15}$$ (4–12)	1.0034	1.0272	0.99351	0.9916	0.9	1.00276	1.1	1.03139	0.9687
$${T}_{36}$$ (28–27)	0.9739	0.9991	0.96845	0.9681	0.92811	0.96587	0.93863	1.01569	0.9741
$${Q}_{10}$$ (MVAR)	4.70492	2.2025	4.99909	4.70924	0	2.817585	2.684852	0.448918	1.5896
$${Q}_{12}$$ (MVAR)	4.24528	0.16911	4.98579	4.42300	0	3.946360	1.910281	1.919497	1.1263
$${Q}_{15}$$ (MVAR)	3.79860	0.49680	2.70386	4.060068	3.142928	0	3.171857	0.678525	4.2301
$${Q}_{17}$$ (MVAR)	3.3856	0.0429	3.61731	4.408631	0.090808	4.52756	0.950972	2.036575	4.9719
$${Q}_{20}$$ (MVAR)	4.23117	1.49543	4.99426	4.50550	3.120945	4.041979	4.116303	2.995657	4.0218
$${Q}_{21}$$ (MVAR)	4.82997	2.37321	4.81975	4.73517	2.399678	4.949713	3.747557	1.134746	4.9972
$${Q}_{23}$$ (MVAR)	4.3502	0.11461	4.99194	4.11112	1.385095	4.843498	2.152461	1.896188	2.9141
$${Q}_{24}$$ (MVAR)	3.9253	1.30597	4.9990	4.72998	0.340715	4.954122	0.384783	4.131996	5
$${Q}_{29}$$ (MVAR)	2.69775	0.81080	1.11435	2.57926	1.766579	0.412344	0.010386	2.672235	2.4753
Fuel cost ($/h)	799.18	801.60	799.135	799.17	807.179	799.193	800.877	799.962	800.5106
Power losses (MW)	8.6151	9.1477	8.64736	8.6636	10.0683	8.62662	9.21300	8.65502	–
Voltage deviations (pu)	1.6377	0.6024	1.71252	1.7326	0.57608	1.63148	1.36734	0.63584	–
Iterations time (s)	50.8	418	52	91.2	85.097	52.1	53.8	55.2	–

Also, Table 6 illustrates comparative results for minimizing the fuel cost (Case 1) with several other algorithms which are developed GWO^21^, Adaptive GO^27^, MOQRJFS^28^, CSO^35^, NBA^68^, MCSO^35^, IMFO^36^ and ECHT-DE^37^. As shown, the proposed mAHA obtain the minimum cost of 799.135 $/h among other techniques.

**Table 6:** Comparison results for minimizing the fuel costs (Case 1).

Method	Fuel cost ($/h)	Method	Fuel cost ($/h)
MCSO^35^	799.3332	MOQRJFS^28^	799.1065
ECHT-DE^38^	800.4148	GWO^21^	800.433
IMFO^36^	800.3848	AGO^27^	800.0212
NBA^35^	799.7516	CSO^35^	799.8266

##### Case 2: minimization of active power losses

**Figure 8:**
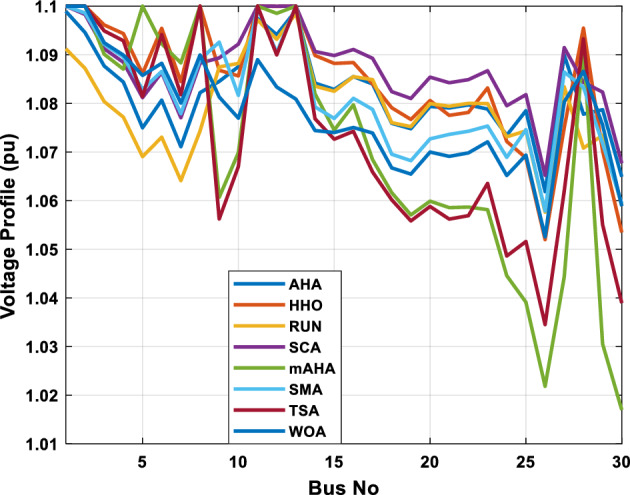
The voltage profile of the compared techniques for case 2.

**Figure 9:**
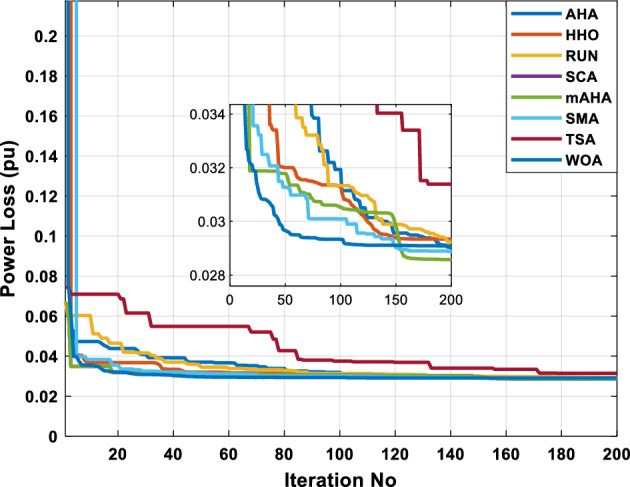
The convergence characteristics of all methods for case 2.

This scenario involves minimizing real power loss as a single objective function. A comparison of the optimum simulation results obtained by the mAHA technique with those obtained by other methods is presented in Table 7. A real power loss of 2.85767 MW was achieved using the mAHA methodology. Alternatively, the other techniques achieved values ranging from 2.90269 to 3.54983 MW. The voltage magnitudes on all buses are within their acceptable ranges as shown in Fig. 8. According to Fig. 9, the mAHA method and other techniques exhibit similar convergence characteristics in terms of minimizing real power loss. From this figure, it is evident that mAHA reaches its optimum solution faster than other methods.

**Table 7:** Optimum control variables for IEEE 30-bus grid for minifying real power loss.

Control variables	AHA	HHO	mAHA	RUN	SCA	SMA	TSA	WOA	CGSCE^50^
$${P}_{G1}$$ (MW)	52.3425	51.33418	51.25777	52.16676	59.8116	51.2878	53.889	51.308	51.5010
$${P}_{G2}$$ (MW)	79.7942	80	80	79.7063	78.5750	80	80	80	79.9997
$${P}_{G5}$$ (MW)	49.9318	50	50	49.9449	50	50	48.4452	50	50
$${P}_{G8}$$ (MW)	34.9041	35	34.9999	34.9542	35	35	35	35	34.9999
$${P}_{G11}$$ (MW)	29.6905	30	29.9999	29.9962	23.5632	30	30	30	30
$${P}_{G13}$$ (MW)	39.6394	40	40	39.5551	40	40	39.2044	40	40
$${V}_{1}$$ (pu)	1.09893	1.1	1.1	1.09122	1.1	1.1	1.1	1.1	1.0621
$${V}_{2}$$ (pu)	1.09457	1.1	1.09829	1.08720	1.1	1.0985	1.1	1.1	1.0579
$${V}_{5}$$ (pu)	1.07498	1.08611	1.08119	1.06905	1.1	1.0828	1.08142	1.08575	1.0385
$${V}_{8}$$ (pu)	1.08221	1.1	1.08827	1.07432	1.1	1.0890	1.1	1.08995	1.0448
$${V}_{11}$$ (pu)	1.09747	1.1	1.1	1.09716	1.1	1.0996	1.1	1.08898	1.0791
$${V}_{13}$$ (pu)	1.09909	1.1	1.1	1.09850	1.1	1.1	1.1	1.08088	1.0558
$${T}_{11}$$ (6–9)	1.01155	1.01589	1.00979	0.99453	1.08586	0.98000	1.1	1.00356	1.0824
$${T}_{12}$$ (6–10)	0.93759	0.99626	0.95378	0.93651	0.9	1.02196	0.91405	1.00289	0.9017
$${T}_{15}$$ (4–12)	0.99101	0.98397	0.98387	0.98839	0.94535	1.00061	0.98785	0.99901	0.9956
$${T}_{36}$$ (28–27)	0.97091	1.01589	0.98040	0.97522	1.03166	0.97447	1.01656	0.99591	0.9772
$${Q}_{10}$$ (MVAR)	3.89421	5	4.642197	3.39678	0	4.98964	0.76948	5	2.1245
$${Q}_{12}$$ (MVAR)	4.77147	5	4.852034	4.87548	2.73792	4.65688	3.43345	5	2.1490
$${Q}_{15}$$ (MVAR)	3.84970	5	4.773109	3.43138	0	0.33029	2.91064	5	4.2533
$${Q}_{17}$$ (MVAR)	4.12313	5	4.758789	4.90404	1.53436	4.58416	3.51662	5	4.9964
$${Q}_{20}$$ (MVAR)	4.46517	5	4.920185	4.66356	0.828348	3.93139	1.90675	5	3.9417
$${Q}_{21}$$ (MVAR)	4.41188	5	4.999950	4.02067	2.90876	5	1.01628	5	5
$${Q}_{23}$$ (MVAR)	3.26619	4.97466	4.482629	4.63745	0	4.99994	4.34705	5	2.9168
$${Q}_{24}$$ (MVAR)	4.33241	5	4.992978	4.64350	0	4.25083	0.21415	5	4.9992
$${Q}_{29}$$ (MVAR)	2.62251	5	3.373429	3.10008	1.91637	1.39732	4.25971	5	2.3996
Fuel cost ($/h)	964.688	967.266	967.084	965.11	953.377	967.156	958.301	967.205	967.663
Power losses (MW)	2.90269	2.93419	2.85767	2.9237	3.54983	2.88785	3.13870	2.90849	3.10060
Voltage deviations (pu)	1.91941	1.95581	2.04259	1.86249	1.53477	1.86299	1.60215	1.79152	0.89096
Iterations time (s)	47.74	337.6	50.8	58	32.37	33.61	31	34.54	–

##### Case 3: minimization of total voltage deviation

**Figure 10:**
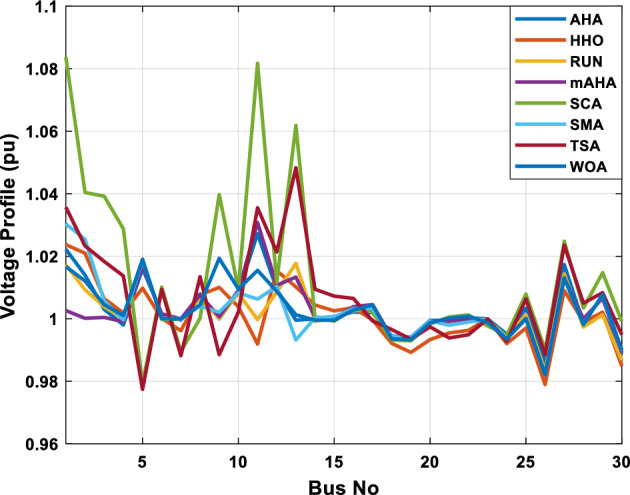
The voltage profile of the compared methods for case 3.

**Figure 11:**
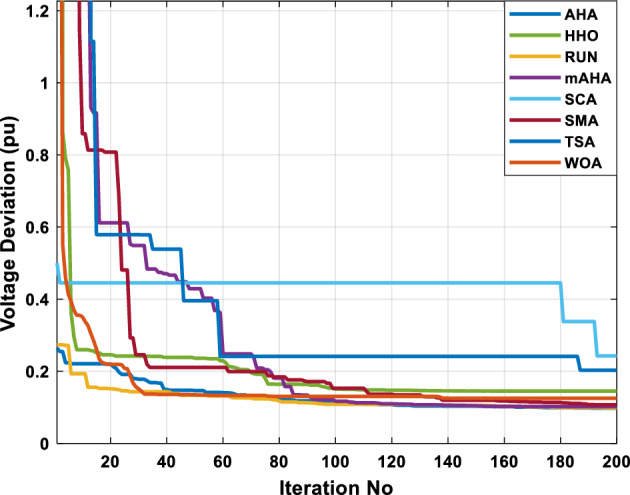
The convergence characteristics of the methods for case 3.

The mAHA technique is employed in this scenario to minimize the total voltage deviation, as discussed in section "Preliminaries". It is shown in Table 8 that the mAHA technique achieved optimal variables in comparison to the other algorithms. It is evident from the results that mAHA achieved the best and minimum voltage deviation values of 0.09783 pu, outperforming other algorithms such as AHA, HHO, RUN, SCA, SMA, TSA, and WOA, which resulted in values of 0.09841 pu, 0.14498 pu, 0.10214 pu, 0.24245 pu, 0.10708 pu, 0.20299 pu, and 0.12508 pu, respectively. Figure 10 illustrates that mAHA provides the most accurate voltage profile compared to other algorithms. Furthermore, Fig. 11 demonstrates that mAHA's convergence characteristic outperforms the other compared algorithms.

**Table 8:** Optimal control variables for IEEE 30-bus test system for minimizing voltage deviation.

Control variables	AHA	HHO	mAHA	RUN	SCA	SMA	TSA	WOA	AHA^49^
$${P}_{G1}$$ (MW)	122.681	88.5591	147.117	75.2296	184.529	134.813	161.712	72.1794	–
$${P}_{G2}$$ (MW)	62.3952	76.2285	57.4035	72.5051	28.1335	60.5820	40.6097	73.5926	9.7903
$${P}_{G5}$$ (MW)	42.4997	38.4568	34.7601	47.9036	24.0555	23.3362	37.3022	45.4916	45.8976
$${P}_{G8}$$ (MW)	25.3713	32.2721	11.8578	32.3474	21.0647	31.0750	14.2072	31.2644	21.7849
$${P}_{G11}$$ (MW)	23.9567	16.1774	27.3105	28.0929	14.9899	27.0478	15.0217	27.2625	28.3488
$${P}_{G13}$$ (MW)	12.9713	36.8881	13.0244	31.8296	20.5300	14.0200	22.9069	37.9501	18.0528
$${V}_{1}$$ (pu)	1.01725	1.02372	1.02223	1.00262	1.08376	1.03034	1.03565	1.01670	1.01222
$${V}_{2}$$ (pu)	1.00930	1.02097	1.01398	1.00015	1.04039	1.02543	1.02329	1.01209	0.99700
$${V}_{5}$$ (pu)	1.01912	1.00979	1.0160	1.01700	0.97922	1.01881	0.97742	1.01899	1.01962
$${V}_{8}$$ (pu)	1.00651	1.00760	1.00794	1.00759	1.00015	1.00403	1.01344	1.00446	1.00738
$${V}_{11}$$ (pu)	0.99973	0.99193	1.02722	1.03076	1.08171	1.0063	1.03549	1.01549	1.03968
$${V}_{13}$$ (pu)	1.01772	1.01021	0.99954	1.01332	1.06184	0.99320	1.04831	1.001288	1.03656
$${T}_{11}$$ (6–9)	1.0117	0.96021	1.04337	1.04569	0.95573	1.01075	1.1	0.957383	0.99107
$${T}_{12}$$ (6–10)	0.91206	0.96042	0.90460	0.90003	1.08799	0.9	0.91578	0.976089	0.93416
$${T}_{15}$$ (4–12)	0.99164	0.96411	0.95435	0.98536	1.09168	0.94867	1.01159	0.979018	1.00823
$${T}_{36}$$ (28–27)	0.96299	0.97707	0.95676	0.97327	0.95721	0.97493	0.95270	0.971677	0.95622
$${Q}_{10}$$ (MVAR)	3.27787	1.97271	4.18088	4.42679	4.69157	1.787223	3.36870	4.739554	3.99263
$${Q}_{12}$$ (MVAR)	1.15581	4.12753	1.33663	4.20977	2.12152	4.430132	2.61065	4.453511	1.90580
$${Q}_{15}$$ (MVAR)	4.69677	3.76231	4.48546	2.95974	1.27970	3.666590	4.70315	4.372774	4.12228
$${Q}_{17}$$ (MVAR)	4.51875	2.59322	1.51701	4.45045	0.27270	3.545235	2.41784	4.383086	2.42501
$${Q}_{20}$$ (MVAR)	4.74382	2.45081	4.93432	4.72581	4.07621	4.950436	5	4.293828	4.99457
$${Q}_{21}$$ (MVAR)	4.45496	4.94036	4.07387	2.92357	4.96282	1.487236	4.10688	4.354775	4.84730
$${Q}_{23}$$ (MVAR)	4.92754	4.97427	4.63567	4.48689	3.58842	5	2.28231	4.839135	4.21244
$${Q}_{24}$$ (MVAR)	4.82808	4.99962	4.84966	4.60890	3.25991	4.999492	3.934234	3.941876	4.38256
$${Q}_{29}$$ (MVAR)	2.31166	4.41481	1.58313	4.62710	3.29670	5	1.557113	4.941514	1.33206
Fuel cost ($/h)	851.678	885.835	827.401	920.571	814.934	820.855	826.089	922.282	860.1368
Power losses (MW)	6.47578	5.18227	8.07451	4.50839	9.90363	7.47450	8.36049	4.34096	10.44553
Voltage deviations (pu)	0.09841	0.14498	0.09783	0.10214	0.24245	0.10708	0.20299	0.12508	0.120906
Iterations time (s)	27.5	277.36	36	47.358	33	28	27.2	32	–

##### Case 4: minimization of fuel cost and power losses

**Figure 12:**
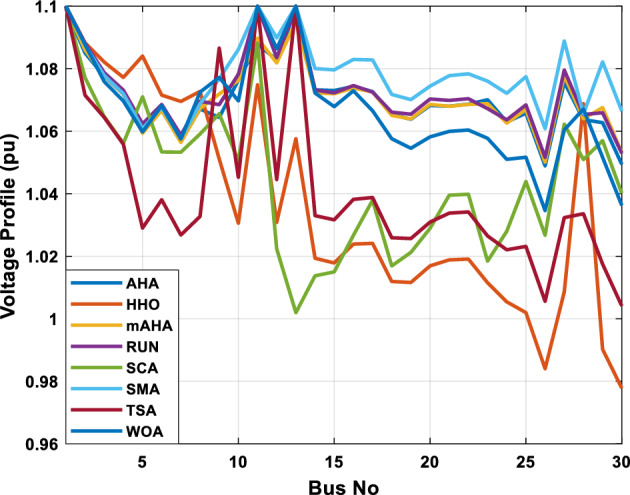
The voltage profile of the mAHA and other compared algorithms for case 4.

**Figure 13:**
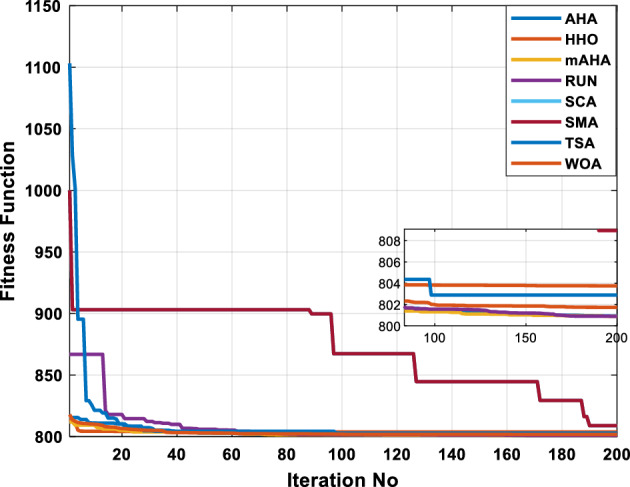
The convergence characteristics of mAHA and other compared algorithms for case 4.

A multi-objective function is considered in this case, which aims to minimize fuel cost and real power loss. A comparison of the most reliable simulation results obtained using the mAHA technique is presented in Table 9. Based on the mAHA technique, an objective function value of 801.8704 was obtained, significantly better than that obtained through other methods, including AHA, HHO, RUN, SCA, SMA, TSA, and WOA. Figure 12 illustrates that all voltage profiles of the buses were within their limits. As shown in Fig. 13, the convergence characteristics of the mAHA technique and the other compared techniques are related to the minimization of the cost function. Therefore, it can be concluded that the mAHA technique performs better than other algorithms when minimizing the cost function.

**Table 9:** Optimum control variables for the 30-bus grid to minimize fuel cost and power losses.

Control variables	AHA	HHO	mAHA	RUN	SCA	SMA	TSA	WOA	FKH^40^
$${P}_{G1}$$ (MW)	177.0931	175.5109	176.0591	176.4130	189.5175	176.4369	176.6683	173.564	100.8346
$${P}_{G2}$$ (MW)	48.76131	48.46011	48.71275	48.74285	37.16635	48.07728	48.43647	46.9036	54.8671
$${P}_{G5}$$ (MW)	21.4638	19.38189	21.47426	21.48944	17.45434	21.30445	20.28966	20.47343	38.1537
$${P}_{G8}$$ (MW)	20.51172	16.32334	21.39346	21.41286	21.01935	21.6203	23.41367	26.21112	34.9623
$${P}_{G11}$$ (MW)	12.0561	15.52938	12.32207	11.9425	10.393	12.28089	11.71707	11.55522	30
$${P}_{G13}$$ (MW)	12.17641	17.14297	12.00147	12	17.87807	12.26235	12	13.15282	28.7706
$${V}_{1}$$ (pu)	1.099296	1.1	1.1	1.1	1.1	1.099784	1.1	1.1	1.1
$${V}_{2}$$ (pu)	1.085022	1.088287	1.087533	1.088118	1.077111	1.085994	1.071508	1.087778	1.0929
$${V}_{5}$$ (pu)	1.060608	1.084032	1.060822	1.062336	1.071001	1.059365	1.029004	1.059761	1.0719
$${V}_{8}$$ (pu)	1.067104	1.072722	1.068763	1.069603	1.059109	1.067244	1.032739	1.0725	1.0835
$${V}_{11}$$ (pu)	1.087851	1.074855	1.099949	1.099836	1.088153	1.089793	1.1	1.1	1.0997
$${V}_{13}$$ (pu)	1.099112	1.057605	1.099997	1.099998	1.001946	1.095	1.1	1.1	1.1
$${T}_{11}$$ (6–9)	1.045693	1.003073	1.029156	1.0473	0.981436	1.018356	0.9	0.998058	1.1329
$${T}_{12}$$ (6–10)	0.907026	1.018954	0.903674	0.900537	0.924547	0.938597	1.1	0.970844	0.9
$${T}_{15}$$ (4–12)	1.004052	1.069846	0.988155	0.998731	0.998942	1.002393	1.088772	0.968337	1.0031
$${T}_{36}$$ (28–27)	0.974703	1.041105	0.969473	0.970013	0.971083	0.97472	0.985332	0.997868	0.9783
$${Q}_{10}$$ (MVAR)	4.447787	0.707715	4.52375	3.438364	3.783976	4.331209	3.468405	3.297766	3.4906
$${Q}_{12}$$ (MVAR)	3.859251	1.196239	3.97058	2.846555	1.32943	4.9296261	1.47400	2.58092	4.079
$${Q}_{15}$$ (MVAR)	4.900656	2.932422	4.84955	3.787102	0	4.6612761	2.140105	0.925562	5
$${Q}_{17}$$ (MVAR)	3.816908	1.719433	4.9999	0.948367	0	4.0272437	1.75447	1.913396	0.2021
$${Q}_{20}$$ (MVAR)	4.183282	2.696676	2.15121	4.972533	2.3980128	4.7595398	2.298828	1.26444	4.7291
$${Q}_{21}$$ (MVAR)	4.589494	2.431736	4.62922	4.998385	2.7757761	4.8752976	1.98450	3.69092	4.1547
$${Q}_{23}$$ (MVAR)	4.46654	2.33526	2.854919	1.961131	1.3605141	3.980208	2.26344	0.54035	5
$${Q}_{24}$$ (MVAR)	4.896263	3.115011	4.9656	5	3.8662456	4.590352	3.144415	3.59317	0.0054
$${Q}_{29}$$ (MVAR)	2.06742	0.597591	4.27857	1.788983	4.8538048	3.024216	1.622321	3.617967	1.0601
Objective function	801.9555	804.7762	801.8704	801.9097	809.9703	801.9277	803.9152	802.7551	–
Fuel cost ($/h)	799.2024	801.966	799.1388	799.17	806.9349	799.1922	801.0698	800.0492	860.9599
Power losses (MW)	8.662526	8.948682	8.56314	8.600676	10.02863	8.582226	9.125181	8.4602	4.1883
Voltage deviations (pu)	1.622949	0.720765	1.814924	1.658613	0.933144	1.638002	0.818398	1.485045	1.7751
Iterations time (s)	32	280	40.6	54	28.2	33.46	30.308	28.1	–

##### Case 5: minimization of fuel cost and total voltage deviation

**Figure 14:**
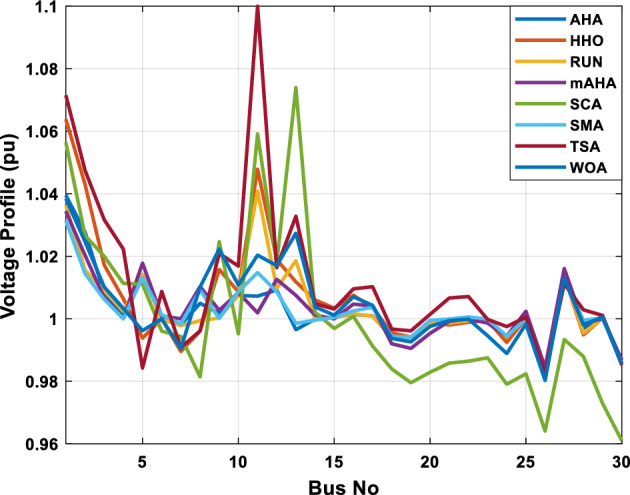
The voltage profile of the compared techniques for case 5.

**Figure 15:**
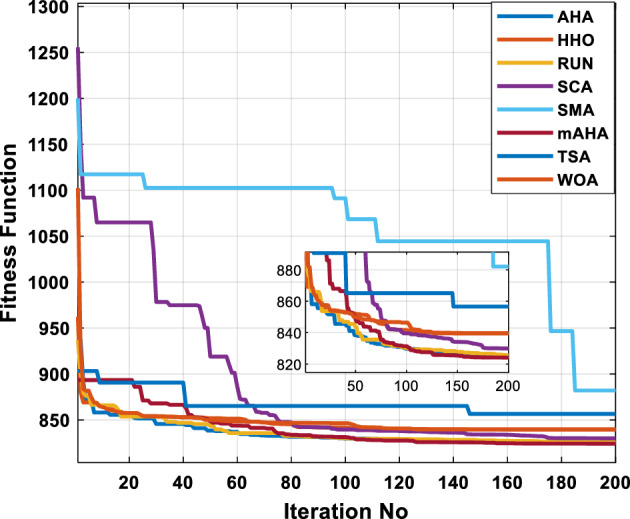
The convergence characteristics of all compared methodologies for case 5.

Fuel cost and voltage deviation are minimized in this case, which is considered a multi-objective function. Table 10 compares the most promising simulation results obtained using the mAHA technique with those obtained using other approaches. The mAHA technique yielded an objective function value of 824.0697, which is better than the values obtained using other techniques, such as AHA, HHO, RUN, SCA, SMA, TSA, and WOA, which yielded values of 824.9193, 839.7303, 829.941, 882.0512, 825.729, 856.5994, and 839.5122, respectively. The voltage profiles of all buses were found to be within their limits, as shown in Fig. 14. Based on Fig. 15, the mAHA technique and other comparable techniques are compared in terms of minimizing the cost function. As a result, it can be concluded that the mAHA technique performs better than the other algorithms when minimizing the cost function.

**Table 10:** Optimum control variables for the 30-bus system for minifying fuel cost and voltage deviation.

Control variables	AHA	HHO	mAHA	RUN	SCA	SMA	TSA	WOA	GWO^37^
$${P}_{G1}$$ (MW)	175.5229	165.2596	174.9996	180.2959	152.897	176.000	152.3759	175.6838	63.4100
$${P}_{G2}$$ (MW)	48.78027	54.90978	47.908	43.11046	46.21225	49.3706	51.20082	42.94417	77.7900
$${P}_{G5}$$ (MW)	21.95667	19.35698	21.18578	24.74529	23.88146	21.9316	21.44287	21.97456	39.8500
$${P}_{G8}$$ (MW)	21.46253	23.8677	23.89826	19.11117	35	19.29766	32.45254	23.89286	45.4400
$${P}_{G11}$$ (MW)	13.18521	13.86115	13.32854	13.73714	15.75959	13.41639	10.82703	15.50411	30.3100
$${P}_{G13}$$ (MW)	12.32151	15.39698	12.00118	12.49682	18.01988	13.34685	23.45908	12.97393	30.4100
$${V}_{1}$$ (pu)	1.039515	1.063826	1.031421	1.034383	1.056401	1.0364	1.071509	1.03843	1.0720
$${V}_{2}$$ (pu)	1.02771	1.043007	1.014417	1.02032	1.02664	1.015553	1.047492	1.024674	1.0710
$${V}_{5}$$ (pu)	1.014143	0.993806	1.013213	1.017806	1.011091	1.01422	0.984239	0.996189	1.0310
$${V}_{8}$$ (pu)	1.004954	0.996013	1.009208	1.010292	0.981409	0.99934	0.996136	1.010137	1.0041
$${V}_{11}$$ (pu)	1.007247	1.047857	1.014779	1.001898	1.059162	1.04089	1.1	1.020358	1.0400
$${V}_{13}$$ (pu)	0.996524	1.011814	0.998543	1.007497	1.073992	1.018594	1.032821	1.027376	1.0820
$${T}_{11}$$ (6–9)	1.012959	1.000785	1.030949	1.006006	0.950253	1.054622	1.060472	0.954086	1.043
$${T}_{12}$$ (6–10)	0.913714	0.94624	0.9	0.9000	1.1	0.903902	0.914689	0.93967	0.99
$${T}_{15}$$ (4–12)	0.952708	0.947149	0.967012	0.955314	1.048264	0.989138	1.02256	0.973295	0.99
$${T}_{36}$$ (28–27)	0.95967	0.959914	0.970758	0.958486	0.96805	0.96049	0.978803	0.963043	0.965
$${Q}_{10}$$ (MVAR)	4.648409	2.36515	2.173401	1.51318	4.10456	4.259120	3.0328	2.0042	18.93
$${Q}_{12}$$ (MVAR)	0.609357	3.4281	3.8315	1.82596	3.8632	0.579030	4.5746	3.06927	0
$${Q}_{15}$$ (MVAR)	4.843797	0.239426	4.93276	1.8356	0.02045	4.11300	1.63617	0.89053	0
$${Q}_{17}$$ (MVAR)	1.4157006	1.35966	4.0660	3.97469	0.01532	1.103097	1.9630	0.29389	0
$${Q}_{20}$$ (MVAR)	4.869775	3.910071	4.99048	2.26118	0.4833	4.928113	1.7889	2.41299	0
$${Q}_{21}$$ (MVAR)	4.4165256	1.666834	5	3.9316	4.6215	4.341355	3.74594	2.2095	0
$${Q}_{23}$$ (MVAR)	4.918944	3.8529345	4.9518	4.08318	1.70689	4.951307	2.47034	2.07403	0
$${Q}_{24}$$ (MVAR)	4.610539	3.4359845	4.9373	4.99936	3.86399	4.858122	4.77817	1.028523	15.52
$${Q}_{29}$$ (MVAR)	1.73194	2.169675	2.73016	1.31833	0.002345	1.930208	3.3332	2.539075	0
Objective function	824.9193	839.7303	824.0697	829.941	882.0512	825.729	856.5994	839.5122	916.1964
Fuel cost ($/h)	803.9283	805.3913	804.5488	805.9561	810.8618	804.2747	811.2805	804.7557	916.1764
Power losses (MW)	9.829075	9.252214	9.921366	10.09684	8.370063	9.963252	8.358272	9.573489	–
Voltage deviations (pu)	0.104955	0.171695	0.097605	0.119924	0.355947	0.107271	0.226594	0.173782	0.4935
Iterations time (s)	28.144	289.74	34	48	27.6	25.2	29.4	38.5	–

##### Case 6: minimization of fuel cost and power loss with emission

**Figure 16:**
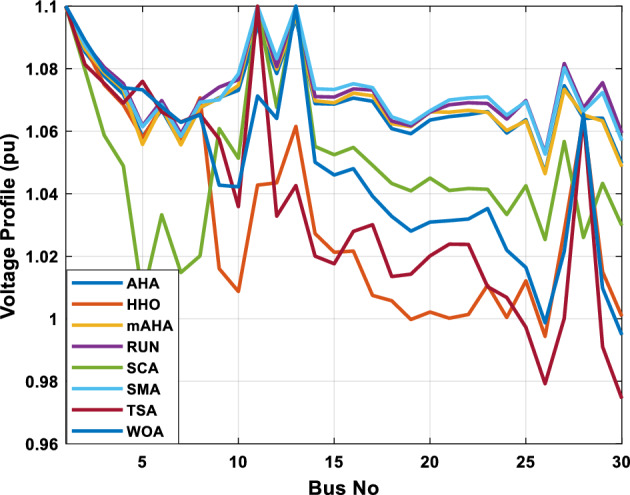
The voltage profile of the mAHA with other compared techniques for case 6.

**Figure 17:**
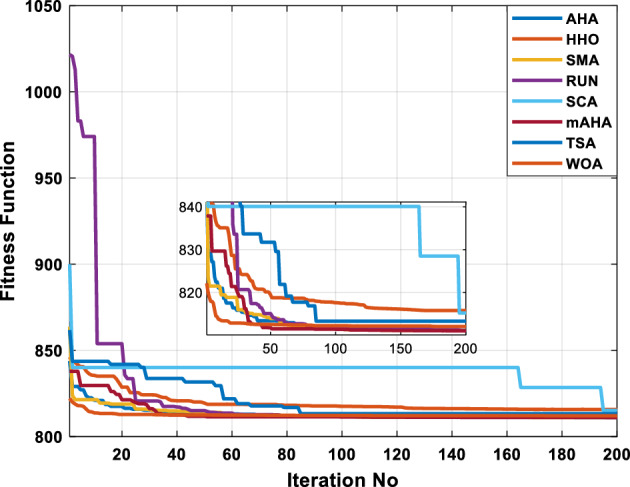
The convergence characteristics of mAHA via other compared methodologies for case 6.

This case involves minimizing fuel costs, losses, and emissions, which are considered multi-objective functions. Table 11 presents simulation results using mAHA and other techniques. The mAHA technique yielded an objective function value of 801.9032, which is better than the values obtained using other techniques such as AHA, HHO, RUN, SCA, SMA, TSA, and WOA, which yielded values of 801.9555, 806.5996, 801.9119, 806.0495, 801.9381, 804.2416, and 802.8859, respectively. The voltage profiles of all buses were found to be within their limits, as shown in Fig. 16. A comparison of mAHA with other compared techniques is shown in Fig. 17 for minimizing the cost function. Based on the comparative results, it can be concluded that the mAHA technique outperforms other algorithms in minimizing the cost function.

**Table 11:** Optimum control variables for the 30-bus network for minifying fuel cost and power loss with emission.

Control variables	AHA	HHO	mAHA	RUN	SCA	SMA	TSA	WOA	GTOT^48^
$${P}_{G1}$$ (MW)	175.5733	177.603	175.767	174.8534	170.4036	175.716	167.5649	177.7528	81.8371
$${P}_{G2}$$ (MW)	48.51211	43.75926	48.69246	49.39471	51.07544	48.56645	48.86481	48.97106	62.4782
$${P}_{G5}$$ (MW)	21.92733	25.29831	21.3409	21.63135	21.66434	21.75938	20.47656	21.13671	38.7375
$${P}_{G8}$$ (MW)	21.84847	12.38672	22.15472	21.90954	20.86486	21.5791	29.01452	20.4536	35
$${P}_{G11}$$ (MW)	11.96228	16.73916	11.98686	12.11297	14.26157	12.15262	13.65286	11.0546	30
$${P}_{G13}$$ (MW)	12.10429	16.46401	12.00784	12.00127	14.33716	12.16831	12.17309	12.9858	40
$${V}_{1}$$ (pu)	1.099951	1.1	1.1	1.1	1.1	1.099641	1.1	1.1	1.0057
$${V}_{2}$$ (pu)	1.085339	1.087013	1.08751	1.088097	1.079569	1.086264	1.081323	1.088988	1.0045
$${V}_{5}$$ (pu)	1.058266	1.057896	1.061403	1.061752	1.006937	1.055758	1.07597	1.073251	1.0003
$${V}_{8}$$ (pu)	1.067498	1.070688	1.069275	1.06982	1.020126	1.067656	1.065421	1.065247	1.0111
$${V}_{11}$$ (pu)	1.093425	1.042768	1.099947	1.099899	1.1	1.099368	1.1	1.07126	1.0007
$${V}_{13}$$ (pu)	1.096013	1.061561	1.1	1.099585	1.1	1.096725	1.04262	1.1	1.0018
$${T}_{11}$$ (6–9)	1.020508	1.062433	1.041327	1.023216	0.992176	1.03108	1.00785	1.050011	1.0137
$${T}_{12}$$ (6–10)	0.945771	1.062433	0.900444	0.938255	0.980633	0.940491	1.028646	0.954795	0.9097
$${T}_{15}$$ (4–12)	1.005387	0.99911	1.00776	1.013246	1.006136	1.009787	1.030953	1.029301	0.9814
$${T}_{36}$$ (28–27)	0.977732	1.008894	0.97747	0.977958	0.949531	0.981172	1.065974	1.036421	0.9741
$${Q}_{10}$$ (MVAR)	4.2671	2.1963	1.95899	1.96925	3.687	4.391137	4.04891	2.78108	5
$${Q}_{12}$$ (MVAR)	1.2816	3.21578	4.40951	3.022015	2.9503	4.63938	4.414316	2.75525	5
$${Q}_{15}$$ (MVAR)	4.76155	0.3136	5	4.497915	4.449	3.56954	0.27440	3.26036	5
$${Q}_{17}$$ (MVAR)	4.41317	0.54456	3.1097588	5	3.5307	4.7432	3.473584	0.96977	5
$${Q}_{20}$$ (MVAR)	3.4149	1.81509	1.773235	3.040964	4.52564	4.48928	2.479389	0.24182	5
$${Q}_{21}$$ (MVAR)	4.59567	2.966	4.450206	5	2.40157	4.57817	3.31851	2.16313	5
$${Q}_{23}$$ (MVAR)	4.6796	1.8182	4.28488	4.10294	0.62822	3.88743	0.831128	2.83587	5
$${Q}_{24}$$ (MVAR)	4.41449	3.906	5	5	1.13921	4.89288	4.159719	2.95156	5
$${Q}_{29}$$ (MVAR)	2.98348	2.235	3.862317	4.50606	2.00105	3.08209	3.729757	2.72833	4.9517
Objective function	801.9555	806.5996	801.9032	801.9119	806.0495	801.9381	804.2416	802.8859	–
Fuel cost ($/h)	799.2317	803.8079	799.1747	799.1933	803.1914	799.2111	801.5644	800.0727	895.4292
Power losses (MW)	8.527801	8.850825	8.550274	8.503275	9.206988	8.542326	8.346742	8.954613	4.6529
Voltage deviations (pu)	1.574832	0.560167	1.676651	1.672981	1.05749	1.592173	0.731068	0.948219	–
Iterations time (s)	54.3	379.4	60	93.2	40.94	56.67	51.76	61.92	–

##### Case 7: minimization of multi objective function without emission

**Figure 18:**
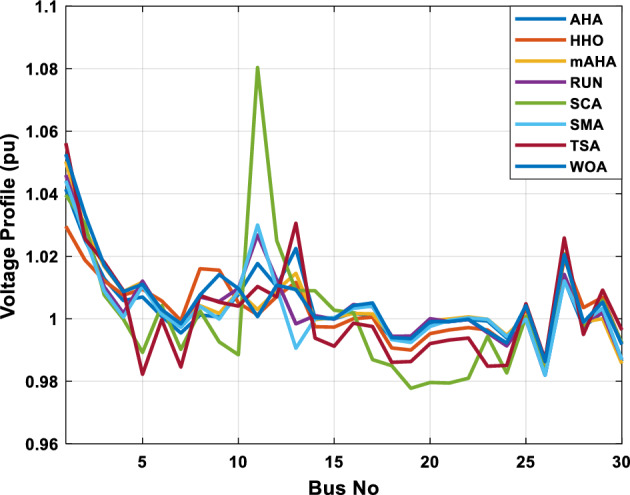
The voltage profile of the mAHA with the other compared techniques for case 7.

**Figure 19:**
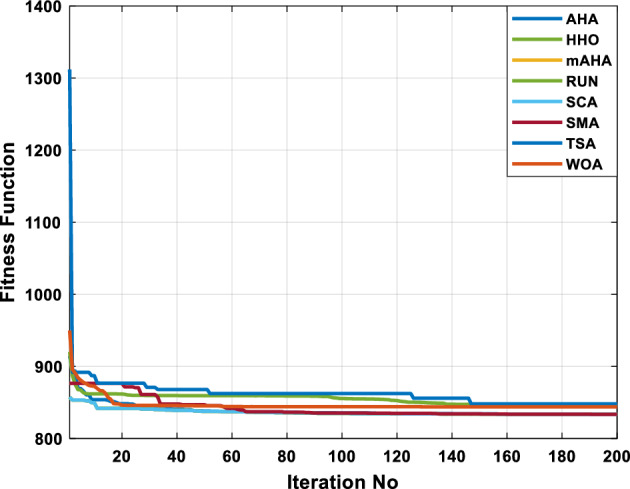
The convergence characteristics of the compared methods for case 7.

Using weighting factors to optimize multiple objective functions simultaneously is recommended, as discussed in section "Application of mAHA: optimal power flow and generation capacity". This is to ensure that the proposed scheme provides maximum benefits. The mAHA technique was compared to other methodologies in Table 12 for solving the multi-objective OPF issue (fuel cost, real power losses, and total voltage deviation) in the IEEE-30 bus network without considering emissions. The results demonstrate that mAHA is more effective than other techniques in solving multiple objectives OF issues. A total objective function value of 833.5196 achieved by mAHA is better than all other methodologies; AHA, HHO, RUN, SCA, SMA, TSA, and WOA achieved results of 833.594, 847.0193, 835.655, 865.4373, 833.594, 848.0131, and 844.0074 without violating the considered constraints. All compared techniques show voltage profiles within the designated limits, similar to previous cases in Fig. 18. Moreover, as shown in Fig. 19, mAHA's convergence characteristics are the fastest.

**Table 12:** Optimum control variables for the 30-bus grid for minifying fuel cost, power loss, and voltage deviation.

Control variables	AHA	HHO	mAHA	RUN	SCA	SMA	TSA	WOA	MFO^29^
$${P}_{G1}$$ (MW)	165.2965	164.3875	168.1762	167.1451	159.818	169.8641	152.589	175.072	199.9683
$${P}_{G2}$$ (MW)	49.8536	40.0599	49.02535	47.2381	32.91117	48.3012	50.35833	35.66866	50.84092
$${P}_{G5}$$ (MW)	22.5235	18.7031	22.57821	24.8307	20.33379	22.7605	25.82388	27.85937	31.36332
$${P}_{G8}$$ (MW)	27.1218	27.0006	25.70569	24.6394	28.65137	26.7184	35	19.00495	35
$${P}_{G11}$$ (MW)	14.6237	15.6899	15.03432	16.4295	20.45342	12.7709	11.19991	16.21916	26.79478
$${P}_{G13}$$ (MW)	12.8555	26.5704	12.0076	12.0587	29.80866	12.0945	16.59451	18.59919	20.56381
$${V}_{1}$$ (pu)	1.04143	1.02956	1.044114	1.04595	1.039584	1.05030	1.056147	1.052629	1.030482
$${V}_{2}$$ (pu)	1.02520	1.01877	1.02626	1.02815	1.030364	1.02846	1.025592	1.033274	1.016681
$${V}_{5}$$ (pu)	1.00695	1.00945	1.010429	1.01207	0.98923	1.01174	0.982243	1.011043	0.999912
$${V}_{8}$$ (pu)	1.00121	1.0160	1.003957	1.00695	1.002509	1.0042	1.007476	1.007605	0.999795
$${V}_{11}$$ (pu)	1.01767	1.00125	1.03	1.02679	1.080396	1.00297	1.010333	1.000669	1.029194
$${V}_{13}$$ (pu)	1.02249	1.01165	0.990579	0.99838	1.009069	1.01454	1.030562	1.00935	1.001948
$${T}_{11}$$ (6–9)	1.02937	0.95655	1.048409	1.02391	1.098176	1.01323	0.995189	0.966127	1.040193
$${T}_{12}$$ (6–10)	0.90356	0.99571	0.900188	0.90018	0.994974	0.90637	0.9	0.940443	1.002741
$${T}_{15}$$ (4–12)	0.99331	0.99752	0.941147	0.94128	0.902913	0.99725	1.012506	0.979369	0.953949
$${T}_{36}$$ (28–27)	0.96987	0.96028	0.973896	0.96402	0.942963	0.96642	0.934657	0.951287	0.979411
$${Q}_{10}$$ (MVAR)	3.85205	3.9022	4.20488	3.00970	4.00770	4.33498	0.71314	3.09115	10
$${Q}_{12}$$ (MVAR)	0.36412	1.7005	0.96678	1.53756	3.76464	3.13246	1.86058	1.77386	− 1.16987
$${Q}_{15}$$ (MVAR)	2.94497	4.10264	4.069	2.23204	0.6013	4.39946	0	4.2319	2.7043
$${Q}_{17}$$ (MVAR)	0.37173	4.30186	4.1482	2.15063	0.0817	0.89085	1.1670	4.3998	1.314517
$${Q}_{20}$$ (MVAR)	4.94578	4.41254	3.5184	4.98895	0	4.9060	4.0954	4.3042	8.443245
$${Q}_{21}$$ (MVAR)	4.95535	4.78612	3.8299	2.51254	1.227	4.85544	2.3899	2.1991	10
$${Q}_{23}$$ (MVAR)	4.84726	4.47705	5	2.59750	3.435	4.83997	0	2.549	3.742131
$${Q}_{24}$$ (MVAR)	4.95638	3.8457	5	3.16762	2.983	4.9685	1.3062	2.8127	10
$${Q}_{29}$$ (MVAR)	3.23331	2.54918	3.7934	2.54487	1.6579	2.14975	1.04908	1.5948	3.803413
Objective functions	833.594	847.0193	833.5196	835.655	865.4373	833.594	848.0131	844.0074	967.59
Fuel cost ($/h)	804.219	813.2852	804.1447	805.128	821.5645	803.542	809.8704	810.5938	830.1046
Power losses (MW)	8.87481	9.011748	9.12738	8.94167	8.576426	9.10983	8.165534	9.023431	6.1289
Voltage deviations (pu)	0.11625	0.157107	0.111202	0.12643	0.2672	0.11832	0.218116	0.153667	0.0899
Iterations time (s)	50	827.522	84.9	68.2	43.74	42.6	46.6	46.54	–

##### Case 8: minimization of multi-objective function with emission

**Figure 20:**
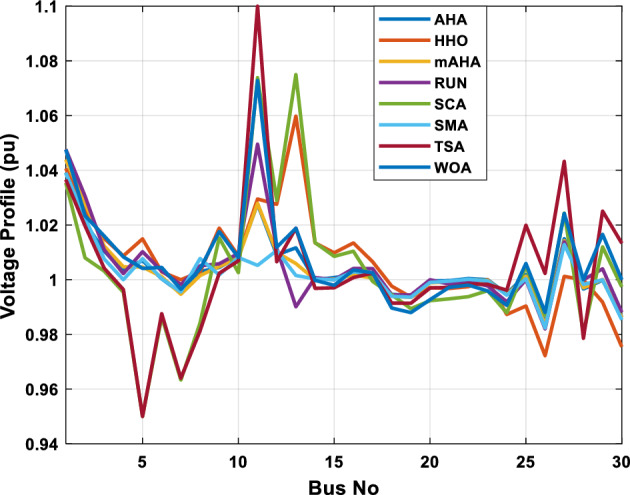
The voltage profile of the mAHA with the other compared techniques for case 8.

**Figure 21:**
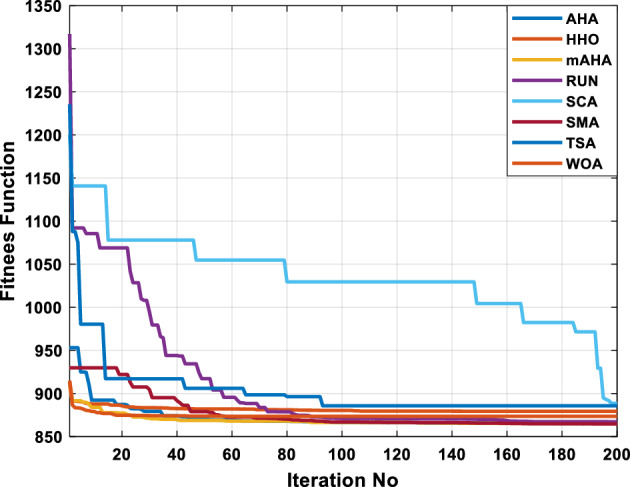
The convergence characteristics of all compared techniques for case 8.

According to Table 13, the mAHA algorithm outperformed the other compared algorithms for solving a multi-objective OPF problem in the IEEE 30-bus testing system. From this table, mAHA offers the best objective function at 864.735 compared to the other techniques. For all algorithms compared in Fig. 20, the voltage profiles indicate that all voltages are within the specified range. As shown in Fig. 21, mAHA has fast convergence, outperforming all other algorithms.

**Table 13:** Optimum control variables for the 30-bus grid for minifying multi-objective function with emission.

Control variables	AHA	HHO	mAHA	RUN	SCA	SMA	TSA	WOA	FKH^40^
$${P}_{G1}$$ (MW)	165.400	166.8056	166.4721	166.5516	166.724	164.969	156.645	168.9629	123.6836
$${P}_{G2}$$ (MW)	47.79336	46.25959	48.60795	51.88644	45.45169	48.78904	50.36477	49.08558	51.5998
$${P}_{G5}$$ (MW)	23.99595	18.1686	22.54116	22.11936	15.25348	23.20754	24.91179	19.7571	31.4264
$${P}_{G8}$$ (MW)	26.37857	27.31501	30.67165	20.14404	26.94864	26.89674	30.9148	20.74778	34.9189
$${P}_{G11}$$ (MW)	15.4945	21.15653	11.53718	15.24274	20.02407	15.25062	10.21388	17.12666	27.1416
$${P}_{G13}$$ (MW)	13.17305	12.81335	12.60599	16.63903	18.62736	13.05962	19.2427	16.99289	20.0125
$${V}_{1}$$ (pu)	1.039098	1.040652	1.039056	1.047613	1.035358	1.043882	1.036548	1.04753	1.1
$${V}_{2}$$ (pu)	1.020842	1.024997	1.022437	1.03056	1.007994	1.027929	1.019686	1.023413	1.0883
$${V}_{5}$$ (pu)	1.007009	1.014929	1.007678	1.010245	0.95	1.004604	0.95	1.004138	1.0626
$${V}_{8}$$ (pu)	1.002955	1.002843	1.007759	1.005067	0.984175	1.001507	0.981303	1.003892	1.0723
$${V}_{11}$$ (pu)	1.027677	1.029506	1.005235	1.049557	1.073876	1.027916	1.1	1.072832	1.0661
$${V}_{13}$$ (pu)	1.01166	1.059816	1.001543	0.990098	1.075011	1.00591	1.018861	1.018827	1.0220
$${T}_{11}$$ (6–9)	1.025236	0.973647	1.012411	1.048338	1.003556	1.023669	1.1	1.022662	1.0909
$${T}_{12}$$ (6–10)	0.909392	0.960301	0.914416	0.90002	0.982152	0.923891	0.9	0.94292	1.0210
$${T}_{15}$$ (4–12)	0.982708	0.992372	0.950994	0.934678	0.991057	0.974637	0.995898	0.987179	1.0619
$${T}_{36}$$ (28–27)	0.959825	0.985456	0.967399	0.968509	0.928281	0.962299	0.9	0.954627	1.0283
$${Q}_{10}$$ (MVAR)	2.75864	0.893957	4.50358	3.88395	1.9883	4.72861	3.7866	2.54853	0.3568
$${Q}_{12}$$ (MVAR)	0.848113	1.099	0.07098	0.965388	0	3.9491	0.0750	3.74059	4.6954
$${Q}_{15}$$ (MVAR)	4.97757	2.832	2.4058	2.3171	2.43085	3.45173	2.6479	1.18119	3.6401
$${Q}_{17}$$ (MVAR)	1.13727	3.328	2.348087	2.8309	0	0.23348	5	2.301628	3.1174
$${Q}_{20}$$ (MVAR)	4.99764	0.975976	5	4.9971	0.83895	4.92498	4.83476	0.974147	0.8760
$${Q}_{21}$$ (MVAR)	4.36739	0.831048	4.995866	0.78898	3.43964	4.86885	1.5173	1.48059	4.9595
$${Q}_{23}$$ (MVAR)	4.7651	3.02398	4.9535	4.29237	0	4.93974	4.6711	4.1572	3.9324
$${Q}_{24}$$ (MVAR)	4.84673	0.84786	5	3.29885	0.56658	4.91916	2.6284	1.3137	5
$${Q}_{29}$$ (MVAR)	1.626306	3.607	2.39323	3.41025	2.51957	1.95153	0.41082	4.1195	1.9857
Objective function	865.0322	879.4284	864.735	867.2717	888.6845	864.9008	885.6557	873.5397	–
Fuel cost ($/h)	804.8632	807.3554	804.8359	804.9669	811.4686	804.3571	810.2148	805.1315	828.3271
Power losses (MW)	8.835931	9.118731	9.036036	9.183196	9.629671	8.772594	8.893501	9.272956	5.3828
Voltage deviations (pu)	0.113104	0.226543	0.106119	0.125655	0.267482	0.118206	0.236763	0.184763	0.4925
Iterations time (s)	46.6	290	56	72.23	45.4	48.6	47.62	57.6	–

##### Case 9: optimal allocation for renewable energy sources for minimizing fuel cost

**Figure 22:**
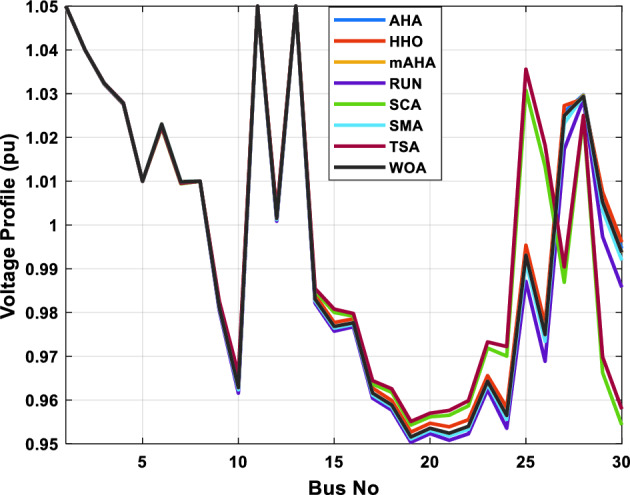
The voltage profile of the compared algorithms for case 9.

**Figure 23:**
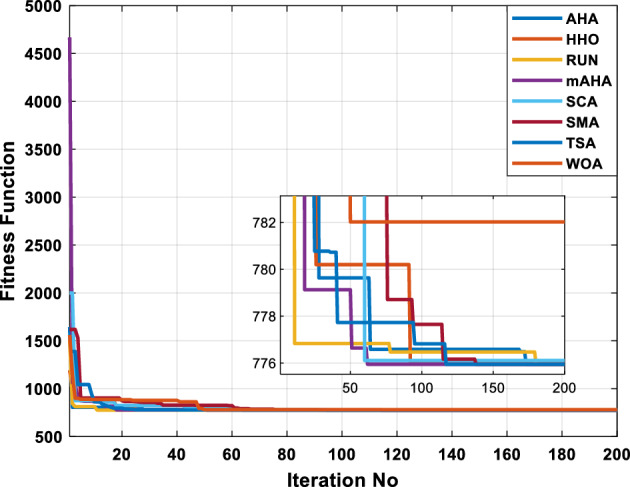
The convergence characteristics of all compared algorithms for case 9.

To validate the efficacy of mAHA's proposed algorithm for integrating renewable sources into the power grid, simulations were carried out on the 30-bus grid to minimize fuel costs. A comparison between the results produced by mAHA and other methodologies can be seen in Table 14. Simulated results show the mAHA technique to be the most efficient, producing the lowest fuel cost at node 27, achieving 775.9469 $/h, outperforming the other techniques. Specifically, the AHA, HHO, RUN, SCA, SMA, TSA, and WOA algorithms achieve results of 775.9475 $/h, 803.5182 $/h, 775.9475 $/h, 776.1083 $/h, 775.9472 $/h, 775.9469 $/h, and 782.0199 $/h, respectively. Additionally, Fig. 22 shows the voltage profile obtained by mAHA, indicating that all bus voltage magnitudes are within acceptable limits. In Fig. 23, mAHA and other compared algorithms are compared regarding their convergence characteristics. It can be seen from the figure that mAHA produces better convergence characteristics than the other algorithms compared. OPF complexity increases as renewable energy sources are integrated into electrical power systems. Based on existing results, this issue has been solved using the mAHA technique.

**Table 14:** Optimum RES allocation for the 30-bus grid to minimize the fuel costs.

Methods	DG location	DG size		${\mathrm{F}}_{\mathrm{cost}}$	${\mathrm{P}}_{\mathrm{loss}}$	VD	Iterations time (s)
		MW	MVAr				
Base Case	–	–	–	11,214.41	5.82226	1.14965	–
AHA	27	47.818	24.865	775.9475	4.40901	0.66019	41.366
HHO	25	48.414	19.661	803.5182	4.40839	0.63996	88.2
mAHA	27	47.818	24.525	775.9469	4.40671	0.66218	40.84
RUN	27	47.818	24.525	775.9475	4.39242	0.68333	68.552
SCA	27	47.818	24.525	776.1083	5.02961	0.67083	47.8
SMA	27	47.812	23.937	775.9472	4.40295	0.66564	47.7
TSA	27	47.812	23.937	775.9469	5.04228	0.65895	28
WOA	27	47.812	23.937	782.0199	4.38679	0.65793	31.6
AHA^49^	25	48.464	24.44	776.0242	5.09091	0.63354	–

##### Case 10: minimization of the fuel cost with the penetration of RES

**Figure 24:**
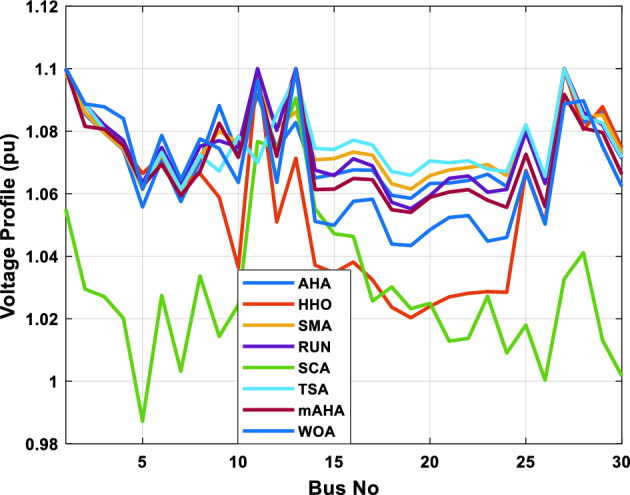
The voltage profile of the compared techniques for case 10.

**Figure 25:**
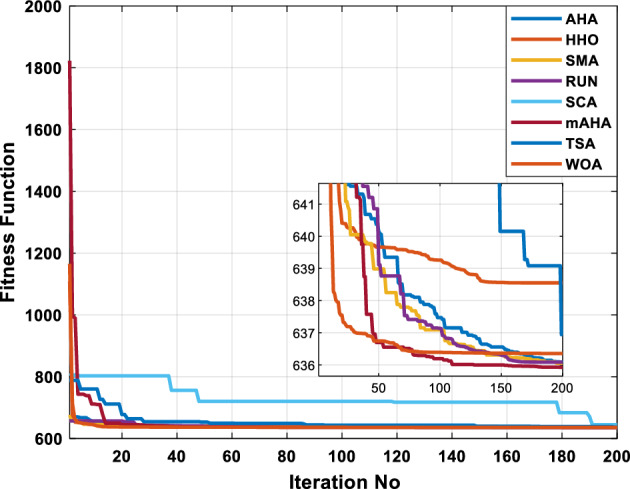
The convergence characteristics of all compared methods for case 10.

To demonstrate the effectiveness of the proposed mAHA technique, it was compared to recent algorithms for minimizing fuel cost in a single objective OPF issue. The modified IEEE 30-bus system used in case 9 was employed, including RES with optimal allocation. Table 15 presents the results, indicating that mAHA achieved the lowest fuel cost of 636.05 $/h, compared to 636.07 $/h, 638.55 $/h, 636.0871 $/h, 644.9163 $/h, 635.9247 $/h, 636.9435 $/h, and 636.3569 $/h obtained by AHA, HHO, RUN, SCA, SMA, TSA, and WOA, respectively. Furthermore, the proposed mAHA algorithm has superior performance compared to case 1. Using the proposed mAHA algorithm in case 1, fuel cost minimization was achieved at 799.135 $/h, which is higher than the cost minimization achieved by integrating renewable energy sources at 636.05 $/h, adding complexity to the OPF issue. As shown in Fig. 24, all buses have voltage profiles within the limits of their capacity. According to Fig. 25, mAHA and other algorithms are comparable regarding fuel cost convergence. Comparing mAHA with other algorithms, the results show that mAHA exhibits superior convergence characteristics.

**Table 15:** Optimum control variables for modified 30-bus grid to decrease the fuel cost.

Control variables	AHA	HHO	mAHA	RUN	SCA	SMA	TSA	WOA	AHA^49^
$${P}_{G1}$$ (MW)	149.92	146.41	149.40	149.404	160.5720	149.6841	149.2895	149.9794	157.6299
$${P}_{G2}$$ (MW)	41.508	37.959	41.629	42.36418	29.66025	42.15954	39.68164	41.46893	42.98818
$${P}_{G5}$$ (MW)	19.256	21.429	19.489	18.94601	19.949	19.17919	20.167	19.69758	19.78803
$${P}_{G8}$$ (MW)	10.233	11.480	10.380	10	11.67302	10	10.27059	10	7.860474
$${P}_{G11}$$ (MW)	10.092	12.503	10.058	10	11.07418	10.014	10.46737	10	7.891434
$${P}_{G13}$$ (MW)	12.047	13.133	12.053	12	12	12	13.1577	12	7.627846
$${V}_{1}$$ (pu)	1.0996	1.1	1.0983	1.1	1.054987	1.1	1.1	1.1	1.098665
$${V}_{2}$$ (pu)	1.0854	1.0885	1.0859	1.088544	1.029493	1.088596	1.081552	1.08872	1.084298
$${V}_{5}$$ (pu)	1.0557	1.0664	1.0613	1.063379	0.987231	1.061839	1.06254	1.061481	1.056581
$${V}_{8}$$ (pu)	1.0705	1.0664	1.0720	1.075225	1.033714	1.072335	1.0672	1.077548	1.065559
$${V}_{11}$$ (pu)	1.0917	1.1	1.0943	1.1	1.076689	1.070003	1.095583	1.096235	1.048832
$${V}_{13}$$ (pu)	1.0828	1.0713	1.0862	1.1	1.090592	1.097708	1.1	1.1	1.047008
$${T}_{11}$$ (6–9)	0.9593	1.0101	0.9989	1.014891	1.1	1.027803	0.979908	1.003916	0.977883
$${T}_{12}$$ (6–10)	0.999	1.0428	0.9337	0.903449	0.9	0.9	0.932093	0.952543	1.030724
$${T}_{15}$$ (4–12)	0.998	1.0101	0.9857	0.993016	0.9	0.982716	1.025951	1.052827	0.998454
$${T}_{36}$$ (28–27)	1.064	1.039	1.0640	1.057892	1.1	1.061452	1.071894	1.077832	1.077131
$${Q}_{10}$$ (MVAR)	2.06449	0.05312	0.36457	1.63973	0	4.165117	2.854821	3.316669	2.603767
$${Q}_{12}$$ (MVAR)	3.03463	0.51095	4.54831	0.74507	2.203478	0.682474	1.274359	2.121810	1.15499
$${Q}_{15}$$ (MVAR)	4.14295	1.25666	4.10624	1.40521	0	4.471524	3.588500	0.858680	1.84191
$${Q}_{17}$$ (MVAR)	3.56717	1.45444	3.80277	0.54270	0	4.878320	0.134908	3.030433	2.21311
$${Q}_{20}$$ (MVAR)	4.34507	0.84688	2.83075	0.05370	4.391702	4.556146	2.405562	1.603250	3.07042
$${Q}_{21}$$ (MVAR)	3.50887	1.71031	3.73776	2.06235	0	4.491089	0.507956	1.118070	3.40776
$${Q}_{23}$$ (MVAR)	4.77626	0.22136	3.59316	0.02950	0	0.192129	2.229644	0.106010	2.98057
$${Q}_{24}$$ (MVAR)	3.92792	0.05312	4.22822	4.08968	0	4.982721	2.621859	1.996898	2.07048
$${Q}_{29}$$ (MVAR)	1.94678	2.22246	1.30591	0.13392		0.360806	2.255161	1.761838	1.299162
Fuel cost ($/h)	636.07	638.55	636.05	636.0871	644.9163	635.9247	636.9435	636.3569	635.8983
Power losses (MW)	7.4721	7.3377	7.4283	7.515853	9.341338	7.449714	7.446704	7.558806	8.850231
Voltage deviations (pu)	1.7053	1.2196	1.7636	1.709431	0.614245	1.788635	1.613575	1.498983	1.11413
Iterations time (s)	57.43	528.7	60	85.16	52.84	47.72	50.8	53.5	–

##### Case 11: minimization of the fuel cost simultaneously with the penetration of RES

To demonstrate the effectiveness of the proposed mAHA algorithm, it was compared to other recent algorithms for solving the OPF problem with a single objective function of minimizing fuel cost. The algorithms were tested on a standard IEEE 30-bus system, and Table 16 shows the results. The mAHA algorithm yielded the lowest fuel cost of 285.8574 $/h, outperforming the other algorithms, which achieved fuel costs of 293.04 $/h, 320.71 $/h, 291.51 $/h, 387.2075 $/h, 285.8574 $/h, 296.68 $/h, and 330.0022 $/h for AHA, HHO, RUN, SCA, SMA, TSA, and WOA, respectively.

**Table 16:** Optimum control variables for the 30-bus network to minimize fuel cost incorporating RES.

Control variables		AHA	HHO	mAHA	RUN	SCA	SMA	TSA	WOA
$${P}_{G1}$$ (MW)		50.0088	49.9948	49.98839	50.081	57.00786	49.9881	49.96689	49.99327
$${P}_{G2}$$ (MW)		20.854	20.934	20	21.198	20	20	20.35843	21.10691
$${P}_{G5}$$ (MW)		15.819	15.843	15	15.1219	15	15	15	21.50999
$${P}_{G8}$$ (MW)		10.020	11.4317	10	10.2984	10.36853	10	10.71447	10.57424
$${P}_{G11}$$ (MW)		10.644	11.962	10	10.186	10	10	10.61799	12.12357
$${P}_{G13}$$ (MW)		12.086	16.890	12	12.129	13.95048	12	13.45181	14.86889
$${V}_{1}$$ (pu)		1.0690	1.0008	1.097983	1.0379	0.95	1.06638	1.1	1.054357
$${V}_{2}$$ (pu)		1.0583	1.0008	1.069677	1.03835	0.950797	1.029039	1.1	1.054776
$${V}_{5}$$ (pu)		1.0179	1.0008	1.032086	0.99193	0.95	0.95551	1.1	1.053414
$${V}_{8}$$ (pu)		1.0505	1.0007	0.990876	1.0223	0.95	0.950402	1.094397	1.027675
$${V}_{11}$$ (pu)		1.0749	1.0008	1.084167	1.0589	1.1	1.099997	0.970912	1.017328
$${V}_{13}$$ (pu)		1.0732	1.0007	0.962325	1.05807	1.1	1.016052	0.952825	1.040024
$${T}_{11}$$ (6–9)		1.0225	0.9476	0.936773	0.9593	0.9	0.902039	1.09546	0.982297
$${T}_{12}$$ (6–10)		0.94082	0.9478	0.936659	0.9805	1.052963	0.980577	0.9	1.028309
$${T}_{15}$$ (4–12)		1.05098	0.9476	0.973809	0.9719	0.9	0.900071	1.086499	0.965849
$${T}_{36}$$ (28–27)		0.96415	0.9479	0.969131	0.9749	0.9	0.903438	1.000388	0.97481
$${Q}_{10}$$ (MVAR)		0.29040	2.11484	0.002158	2.40296	0.001897	1.759759	3.993735	1.270587
$${Q}_{12}$$ (MVAR)		4.65015	1.50013	0.443659	4.51577	0	0.891069	2.952478	0.259110
$${Q}_{15}$$ (MVAR)		0.51761	1.20844	0.973196	3.06932	0	0.112644	0.628124	1.662539
$${Q}_{17}$$ (MVAR)		3.69221	1.11494	0.175960	4.27210	0	2.173984	1.053166	1.429225
$${Q}_{20}$$ (MVAR)		2.23819	2.31343	0.809698	2.42469	3.730087	0	0.717806	1.373039
$${Q}_{21}$$ (MVAR)		3.90940	1.91790	0	2.56433	0	1.765240	3.197998	0.905421
$${Q}_{23}$$ (MVAR)		1.35116	1.72040	0.004937	3.40489	0	4.698625	2.068222	3.155766
$${Q}_{24}$$ (MVAR)		1.90978	0.01803	0.024073	2.79847	0	0	4.775684	0
$${Q}_{29}$$ (MVAR)		2.23253	3.24779	4.252973	2.71411	0	3.110186	4.386053	0.393731
DG location and size	Bus No	28	15	15	28	24	19	24	15
	MW	170.302	171.046	186.2956	171.169	178.2762	196.7573	184.2861	166.7311
	MVAr	21.622	32.2690	0.088258	14.7535	0	0	9.509464	15.90822
Fuel cost ($/h)		293.04	320.71	285.8574	291.51	387.2075	285.8574	296.68	330.0022
Power losses (MW)		6.3360	14.703	19.88383	6.78603	21.20309	30.34557	20.99563	13.50795
Voltage deviations (pu)		1.0895	0.38956	0.433301	0.7986	0.631078	0.504542	0.96487	0.578753
Iterations time (s)		52.52	118.6	57.6	99.2	33.6	57	44.044	54.22

Moreover, the proposed mAHA algorithm's superiority is confirmed compared to previous cases (case 1 and case 10). In case 1 and case 10, the mAHA algorithm achieved fuel cost minimization with values of 799.135 $/h and 636.05 $/h, respectively. These values are higher than the fuel cost achieved by the proposed mAHA algorithm, which solved the OPF problem simultaneously with integrating renewable energy sources and achieved fuel cost minimization with a value of 285.8574 $/h.

**Figure 26:**
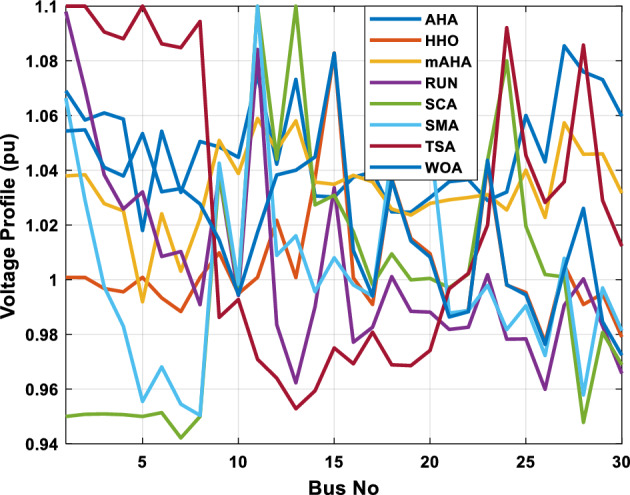
The voltage profile of the compared techniques for case 11.

**Figure 27:**
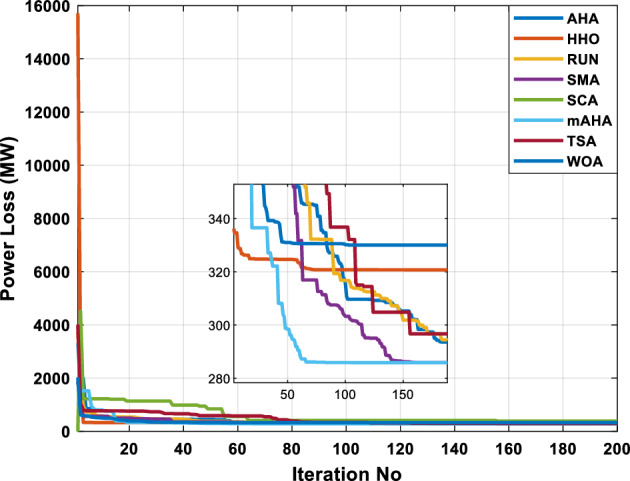
The convergence characteristics of all compared methodologies for case 11.

As can be seen in Fig. 26, all buses are within acceptable voltage limits. As shown in Fig. 27, the mAHA algorithm's convergence characteristics outperform the other compared techniques regarding fuel cost convergence.

**Figure 28:**
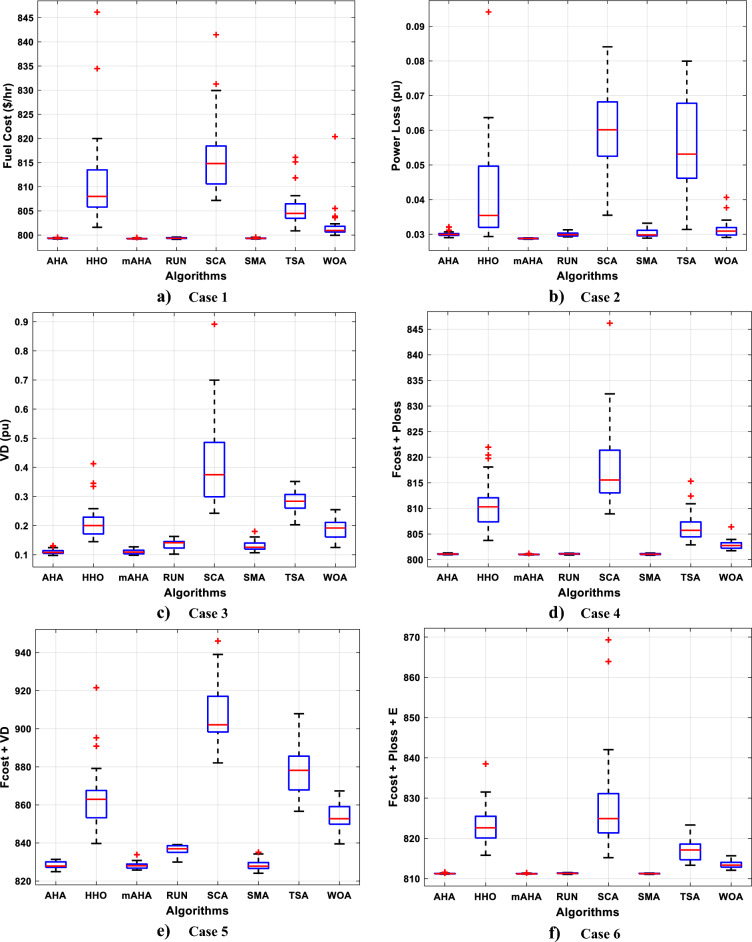

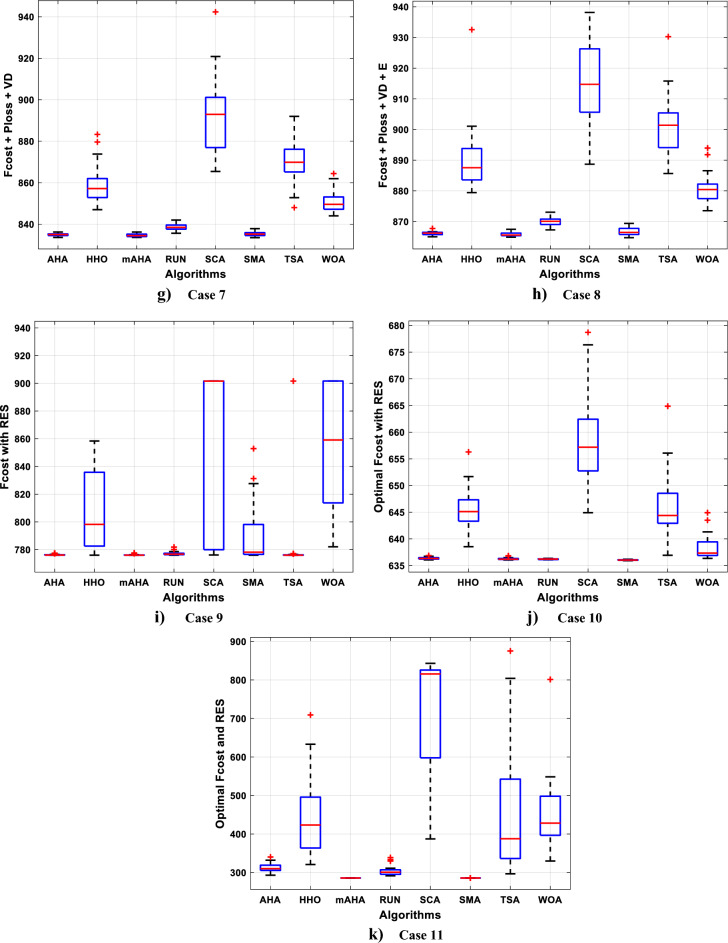
The boxplot of mAHA and other compared methodologies for the IEEE 30-bus grid.

Upon comparing the proposed mAHA's boxplots with the ones of other methods, it can be observed that these are extremely tight for all cases, with the lowest values shown in Fig. 28.

Also, a Wilcoxon signed rank sum test has been done to compare performance between any two algorithms. This test provides a fair comparison between the proposed mAHA method and the other suggested optimization methods on a specific study case using a signed rank test. Store all fitness values over 30 runs of the objective in a case study for both algorithms. Calculate $p$-value which governs the significance of results in a statistical hypothesis test. The argument against null hypothesis ${H}_{0}$ is stronger the smaller the $p$-value. The results obtained using the Wilcoxon signed rank test are offered in Table 17. The column ${H}_{0}$ defines whether the null hypothesis is valid or not. If the null hypothesis is valid (i.e. ${H}_{0}$ = “1” with a significance level, $\alpha$ = 0.05), the performance of the two methods is statistically the same for the study case. The mAHA and AHA perform evenly in cases 1, 3, 4, 5, 6, 7, and 9 while mAHA and SMA are equally in cases 1, 4, and 5. The RUN and TSA performances against AHA are equal in cases 6 and 9 respectively. In the leftover cases, mAHA is found to be superior. Finally, the test findings show that when used to solve the OPF issue in various scenarios, the mAHA outperforms the other optimization approaches, especially for a large number of control variables (large problem) as mentioned in case 11..

**Table 17:** Wilcoxon signed-rank sum test for IEEE 30 bus test system.

Cases	mAHA vs. AHA		mAHA vs. HHO		mAHA vs. RUN		mAHA vs. SCA		mAHA vs. SMA		mAHA vs. TSA		mAHA vs. WOA	
	$\mathrm{p}$-value	${\mathrm{H}}_{0}$	$\mathrm{p}$-value	${\mathrm{H}}_{0}$	$\mathrm{p}$-value	${\mathrm{H}}_{0}$	$\mathrm{p}$-value	${\mathrm{H}}_{0}$	$\mathrm{p}$-value	${\mathrm{H}}_{0}$	$\mathrm{p}$-value	${\mathrm{H}}_{0}$	$\mathrm{p}$-value	${\mathrm{H}}_{0}$
Case 1	0.1236	1	8.8966e−07	0	2.1389e−04	0	8.8966e−07	0	0.0686	1	8.8966e−07	0	8.8966e−07	0
Case 2	8.8966e−07	0	8.8966e−07	0	8.8966e−07	0	8.8966e−07	0	8.8966e−07	0	8.8966e−07	0	8.8966e−07	0
Case 3	0.4979	1	8.8966e−07	0	1.1066e−05	0	8.8966e−07	0	4.1825e−05	0	8.8966e−07	0	8.8966e−07	0
Case 4	0.2470	1	8.8966e−07	0	1.1351e−04	0	8.8966e−07	0	0.0742	1	8.8966e−07	0	8.8966e−07	0
Case 5	0.3614	1	8.8966e−07	0	1.0906e−06	0	8.8966e−07	0	0.3461	1	8.8966e−07	0	8.8966e−07	0
Case 6	0.3090	1	8.8966e−07	0	0.0686	1	8.8966e−07	0	5.6708e−04	0	8.8966e−07	0	8.8966e−07	0
Case 7	0.1039	1	8.8966e−07	0	8.8966e−07	0	8.8966e−07	0	8.8966e−07	0	8.8966e−07	0	8.8966e−07	0
Case 8	0.0089	0	8.8966e−07	0	8.8966e−07	0	8.8966e−07	0	4.2312e−04	0	8.8966e−07	0	8.8966e−07	0
Case 9	0.1868	1	9.8524e−07	0	3.2293e−05	0	8.8966e−07	0	1.4758e−06	0	0.2342	1	8.8966e−07	0
Case 11	8.8966e−07	0	8.8966e−07	0	8.8966e−07	0	8.8966e−07	0	4.7702e−06	0	8.8966e−07	0	8.8966e−07	0

#### IEEE 118-bus grid

To assess the scalability and effectiveness of the mAHA method for resolving large-scale OPF issues, the IEEE 118-bus standard network is considered. The whole data set for this system is cited in^33^. Sixty-four load buses, 54 generating units, and 186 branches make up the network. Switchable shunt capacitors are included on twelve buses: 34, 44, 45, 46, 48, 74, 79, 82, 83, 105, 107, and 110. At lines 8–5, 26–25, 30–17, 38–37, 63–59, 64–61, 65–66, 68–69, and 81–80, nine tap-altering transformers have been installed as shown in Figur 29. All buses have voltage magnitude restrictions between [0.95 pu and 1.1 pu]. Each regulating transformer tap's lowest and maximum values fall within (0.9 1.1) range.

**Figure 29:**
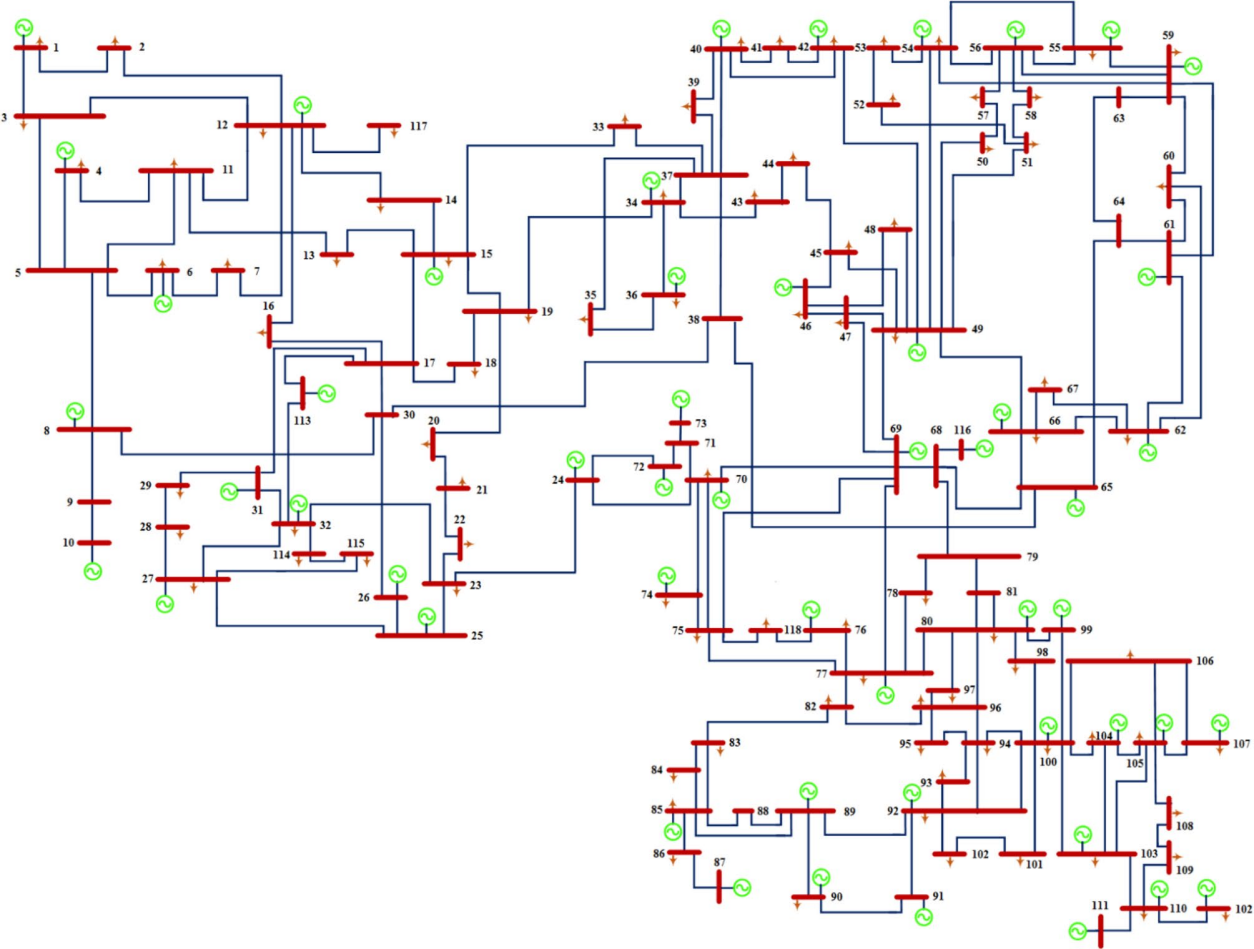
Standard IEEE 118 bus.

##### Case 1: fuel cost minimization

**Figure 30:**
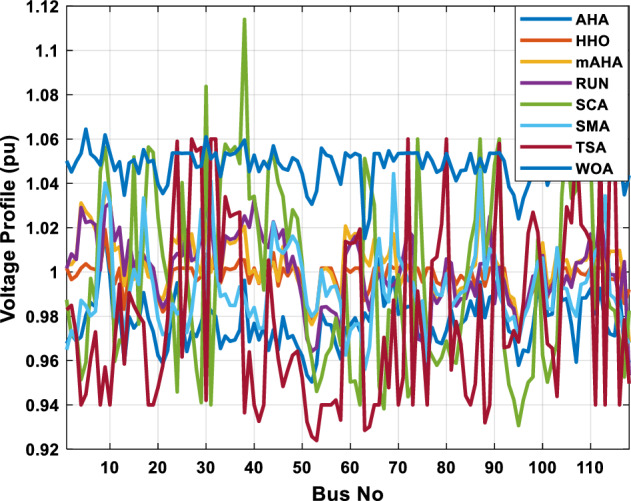
The voltage profile of the compared methodologies for case 1.

**Figure 31:**
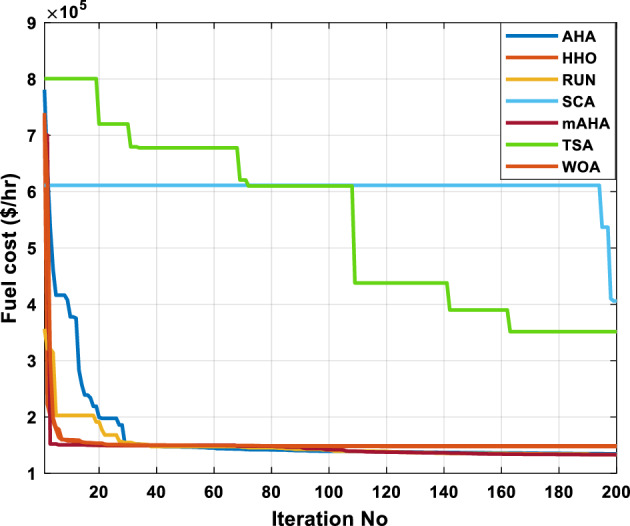
The convergence characteristics of the compared techniques for case 1.

In this part, the OPF issue of the IEEE 118-bus network is solved using the mAHA method without DG. The aim function is cost reduction. Figures 30 and 31 illustrate the voltage profile and cost-saving mAHA algorithm's convergence graph. The graphic demonstrates the mAHA algorithm's good convergence characteristic while handling a significant optimization challenge. Table 18 lists the ideal cost reduction values and control variable modifications. The mAHA algorithm found a better solution. The results show how effective the mAHA technique is in quickly converging on the best answer. These findings demonstrate the mAHA algorithm's effectiveness for resolving significant OPF issues and confirm its scalability.

**Table 18:** Optimum control variables for the 118-bus grid to reduce the fuel cost.

Control variables	AHA	HHO	mAHA	RUN	SCA	SMA	TSA	WOA	SP-DE^38^
$${P}_{G1}$$ (MW)	73.97791	4.499385	22.34	28.51	32.61	62.48	26.51	75.48	30.0317
$${P}_{G4}$$ (MW)	73.97791	4.499385	22.344972	28.512002	32.613835	62.479978	26.507791	75.4781	30.0143
$${P}_{G6}$$ (MW)	45.97772	73.17094	26.185102	26.860476	91.853307	26.809622	1.8723375	70.738237	30.0122
$${P}_{G8}$$ (MW)	29.93431	35.65567	39.501458	16.474302	29.166119	30.453661	95.23658	43.234597	30.0052
$${P}_{G10}$$ (MW)	33.18618	31.77735	34.305901	23.590872	10.268544	43.827001	52.250983	13.845696	317.0191
$${P}_{G12}$$ (MW)	251.1501	187.4009	351.80698	352.67081	286.41893	326.38048	120.60999	465.36772	66.8229
$${P}_{G15}$$ (MW)	58.22862	29.47036	65.718688	63.100463	137.34071	83.13546	68.04316	7.167916	30.0162
$${P}_{G18}$$ (MW)	13.41684	21.87009	0.616095	59.648963	14.099157	33.990822	68.346459	78.719897	30.0063
$${P}_{G19}$$ (MW)	32.04036	39.65482	31.222613	56.384484	8.2057148	50.448569	91.710876	70.658032	30.0495
$${P}_{G24}$$ (MW)	19.10174	56.57999	42.215128	55.01974	1.6884434	19.54139	36.000053	28.267784	30.0111
$${P}_{G25}$$ (MW)	13.98576	46.38767	25.078764	63.230667	17.746971	30.412801	37.796965	61.962804	152.1726
$${P}_{G26}$$ (MW)	189.4145	174.1855	138.94574	186.26184	21.815462	142.72163	99.04144	233.12736	220.8106
$${P}_{G27}$$ (MW)	211.7612	243.0143	222.3762	242.06373	118.33172	186.70857	37.118078	152.35096	30.0364
$${P}_{G31}$$ (MW)	31.05151	6.349815	37.725412	40.554279	4.7761776	10.488302	23.902022	23.015401	32.1004
$${P}_{G32}$$ (MW)	10.93366	25.30334	6.0960596	35.119706	90.605037	8.1223853	89.120842	43.180497	30.0143
$${P}_{G34}$$ (MW)	14.73455	82.59279	34.132257	32.856941	2.0278425	41.702618	11.831651	32.969058	30.0024
$${P}_{G36}$$ (MW)	43.42933	79.18123	42.748537	21.21653	69.961238	56.899056	28.454377	49.000841	30.0132
$${P}_{G40}$$ (MW)	42.09941	61.41026	30.536958	25.977006	94.098715	40.134857	40.323222	63.140272	30.0216
$${P}_{G42}$$ (MW)	70.17412	12.86707	37.884417	38.35962	90.957297	39.956996	6.0282521	6.3599397	30.038
$${P}_{G46}$$ (MW)	71.26958	32.4453	36.026695	53.479184	51.516614	29.959632	30.384363	21.133322	35.7003
$${P}_{G49}$$ (MW)	17.09888	13.58845	19.311066	36.102997	12.384684	20.20281	39.982041	34.514193	162.3848
$${P}_{G54}$$ (MW)	135.2571	172.7611	137.37011	162.0917	68.470381	167.90036	114.96766	171.56946	44.6599
$${P}_{G55}$$ (MW)	50.64071	53.12128	56.939724	23.583623	22.256192	59.888237	148	54.126573	30.0485
$${P}_{G56}$$ (MW)	52.70551	4.66097	37.779088	39.84767	67.238865	60.219718	1.1644984	56.361017	30.0079
$${P}_{G59}$$ (MW)	49.23414	18.42636	44.549145	17.564006	27.886048	55.356403	86.401461	3.5725475	125.3306
$${P}_{G61}$$ (MW)	147.5841	133.5096	120.61706	149.9252	150.36156	123.40271	50.0175	165.77374	124.1197
$${P}_{G62}$$ (MW)	169.4893	199.4909	108.57273	106.47964	218.8781	137.04383	165.83336	65.599081	30.0168
$${P}_{G65}$$ (MW)	6.41605	73.08044	40.944572	31.579011	34.657683	1.9314348	68.574207	26.620347	289.6489
$${P}_{G66}$$ (MW)	255.7686	171.7997	292.77812	196.30856	117.12508	240.05332	62.712269	152.8487	289.1504
$${P}_{G69}$$ (MW)	213.1634	398.099	296.83693	185.15846	35.520406	243.04034	174.06136	148.16923	0
$${P}_{G70}$$ (MW)	31.56371	37.11601	24.484254	27.322567	15.456538	22.941071	93.180001	59.337525	30.0116
$${P}_{G72}$$ (MW)	27.08306	25.62866	0	73.768372	67.391792	4.1033667	100	49.359205	30.0013
$${P}_{G73}$$ (MW)	27.37227	18.52833	37.737553	44.226564	12.025639	46.130819	1.6457489	5.7959714	30.0033
$${P}_{G74}$$ (MW)	43.74254	41.95115	55.615479	28.002771	65.39176	38.237159	48.966833	84.863694	30.0088
$${P}_{G76}$$ (MW)	54.42703	29.86474	35.924267	10.819407	85.272013	52.486266	85.510698	4.1194451	30.0074
$${P}_{G77}$$ (MW)	52.81395	45.55287	26.572018	73.661641	18.892959	26.213381	95.38631	58.854267	30.0141
$${P}_{G80}$$ (MW)	357.4542	82.12018	360.85279	180.68852	247.831	324.2164	426.67001	49.283144	350.9989
$${P}_{G85}$$ (MW)	21.17923	7.799365	2.58E−07	7.77E−01	3.57E+01	3.00E+01	3.78E+01	3.45E+01	30.0087
$${P}_{G87}$$ (MW)	2.631196	13.35761	2.2740058	3.9666089	20.423257	6.7902426	0.4226002	9.3148947	31.2015
$${P}_{G89}$$ (MW)	311.4639	209.0417	403.60339	399.63584	358.45128	364.83383	32.719446	404.48601	379.9452
$${P}_{G90}$$ (MW)	32.7639	53.09261	0.5130444	17.555897	90.922614	15.081347	100	24.944836	30.0443
$${P}_{G91}$$ (MW)	30.05628	23.15726	39.702145	20.049802	23.480439	28.269296	60.073161	35.553433	30.021
$${P}_{G92}$$ (MW)	57.33347	80.33754	32.488743	73.277493	47.547086	30.462968	59.311775	46.100697	30.0162
$${P}_{G99}$$ (MW)	31.61704	40.58355	3.9487838	70.02128	21.549019	2.3238609	29.622289	17.458742	30.0027
$${P}_{G100}$$ (MW)	178.3839	306.9364	155.93808	149.7165	200.49516	197.60342	230.92259	156.79878	177.1013
$${P}_{G103}$$ (MW)	51.27641	50.5826	43.078587	39.795366	123.81709	32.568123	14.427038	93.309792	42.0053
$${P}_{G104}$$ (MW)	9.151841	18.9915	37.806812	16.613335	91.82221	47.154856	99.813121	5.1890923	30.0088
$${P}_{G105}$$ (MW)	34.58507	43.9233	25.50231	19.099752	0.05311	36.200227	86.769711	33.537418	30.0022
$${P}_{G107}$$ (MW)	7.48512	5.020658	41.267475	44.434903	37.644208	27.766235	3.4558507	30.806867	30.013
$${P}_{G110}$$ (MW)	54.41779	35.85211	32.907949	67.016359	89.230502	26.802435	7.7373735	9.6659355	30.0043
$${P}_{G111}$$ (MW)	33.39558	89.46353	32.974757	32.350711	16.981895	33.565675	48.89309	39.344939	40.8014
$${P}_{G112}$$ (MW)	8.857481	70.67769	45.518618	40.794676	94.263477	33.818006	34.885385	38.906668	30.0166
$${P}_{G113}$$ (MW)	24.46341	57.18604	0	32.034234	8.6023708	29.474881	76.7596	36.94084	30.0223
$${P}_{G116}$$ (MW)	35.4573	7.701292	44.746955	21.343602	89.693115	20.858996	53.187439	76.391852	30.0052
$${V}_{1}$$ (pu)	0.967659	1.001713	0.9649205	1.0016137	0.9874263	1.0033598	0.9829249	1.0500917	0.9871
$${V}_{4}$$ (pu)	0.970687	1.001713	0.9871647	1.0290477	0.9513892	1.0311074	0.94	1.0534883	1.0153
$${V}_{6}$$ (pu)	0.98612	1.001713	0.9802136	1.022922	0.9970753	1.0246972	0.9608864	1.0532677	1.008
$${V}_{8}$$ (pu)	1.002829	1.001713	1.0209111	1.0009079	1.0452373	1.0149989	0.94	1.0482726	1.0388
$${V}_{10}$$ (pu)	0.998449	1.001713	1.0315085	1.0310317	1.0343418	1.0320938	0.94	1.053621	1.0494
$${V}_{12}$$ (pu)	0.983058	1.001713	0.9873455	1.0201567	0.9692529	1.0113408	0.9944888	1.0497367	1.002
$${V}_{15}$$ (pu)	0.974677	1.001713	1.001113	1.0014277	1.0519176	0.9967199	0.9847773	1.0506516	0.9988
$${V}_{18}$$ (pu)	0.979009	1.001713	1.0053845	1.0046698	1.0563817	0.9978715	0.94	1.0496127	0.9989
$${V}_{19}$$ (pu)	0.973143	1.001713	0.9943712	1.0017399	1.0539285	0.996978	0.94	1.0503201	0.9986
$${V}_{24}$$ (pu)	0.995082	1.001713	0.981033	1.0083117	0.9458489	1.0130014	1.0589681	1.0537513	1.015
$${V}_{25}$$ (pu)	0.977966	1.001713	0.9827997	1.0072629	1.0403332	1.0249363	0.9616315	1.0535148	1.0298
$${V}_{26}$$ (pu)	0.964085	1.001713	0.9726292	1.0189158	0.9981863	1.0002045	0.9977761	1.0536214	1.0744
$${V}_{27}$$ (pu)	0.973053	1.001713	0.9824432	1.0050693	0.9779946	1.0171781	1.06	1.0535553	1.0045
$${V}_{31}$$ (pu)	0.982447	1.001713	1.0411387	1.0225398	0.94	0.9999279	1.06	1.0510996	0.9992
$${V}_{32}$$ (pu)	0.979873	1.001713	1.0035496	1.0025787	0.9970108	1.0117008	1.06	1.0536976	1.0045
$${V}_{34}$$ (pu)	0.971057	1.001713	0.9899964	1.0162582	1.0576962	1.0153464	1.0339476	1.0537348	1.0141
$${V}_{36}$$ (pu)	0.969313	1.001713	0.9848331	1.0153129	1.0565053	1.0133968	1.0261699	1.0536101	1.0105
$${V}_{40}$$ (pu)	0.976783	1.001713	0.9839382	1.0327823	1.0341608	1.0008319	0.94	1.0527091	1.0001
$${V}_{42}$$ (pu)	0.975279	1.001713	0.9748858	1.0136824	0.9918544	1.0054377	0.94	1.0499071	1.0081
$${V}_{46}$$ (pu)	0.979741	1.001713	1.0084533	1.0189781	1.0341575	1.0114349	0.9483151	1.0466944	1.0316
$${V}_{49}$$ (pu)	0.966236	1.001713	1.0126129	0.9989289	1.0241897	0.996714	0.9642479	1.0500917	1.0429
$${V}_{54}$$ (pu)	0.981281	1.001713	1.0009026	0.9840295	0.9522491	1.0007592	0.94	1.055891	1.0217
$${V}_{55}$$ (pu)	0.976437	1.001713	0.9890595	0.9843174	0.9623507	1.0017023	0.94	1.0528088	1.0215
$${V}_{56}$$ (pu)	0.976024	1.001713	0.9930601	0.9824469	0.9659387	0.9994556	0.94	1.0520192	1.0215
$${V}_{59}$$ (pu)	0.974589	1.001713	0.9624873	1.0079295	0.9987788	1.0207273	1.013766	1.0537264	1.0424
$${V}_{61}$$ (pu)	0.979266	1.001713	0.9763314	1.0160096	0.9507834	1.0173327	1.0133727	1.0534964	1.0496
$${V}_{62}$$ (pu)	0.97347	1.001713	0.9766384	1.0086118	0.94	1.0123299	1.0193777	1.0525318	1.0462
$${V}_{65}$$ (pu)	0.993191	1.001713	0.9989158	0.9938653	1.030179	1.0148225	0.94	1.0534243	1.0623
$${V}_{66}$$ (pu)	1.005867	1.001713	1.0150899	0.9990538	0.948453	1.0092482	0.94	1.053419	1.0593
$${V}_{69}$$ (pu)	0.997562	1.001713	1.0443869	0.9941237	0.9811877	1.0171895	0.9872601	1.0500917	1.0389
$${V}_{70}$$ (pu)	0.980913	1.001713	1.0072379	0.9971868	0.9987397	0.9923827	0.94	1.053458	1.0195
$${V}_{72}$$ (pu)	0.984396	1.001713	0.9962062	1.0187389	0.9436571	1.0030257	1.06	1.0537036	1.0191
$${V}_{73}$$ (pu)	0.984033	1.001713	0.9952997	1.0160158	0.9489485	1.0006245	0.94	1.0535951	1.0234
$${V}_{74}$$ (pu)	0.97218	1.001713	0.9840221	0.9698298	1.06	0.9765873	0.9913531	1.0537809	1.0058
$${V}_{76}$$ (pu)	0.961308	1.001713	0.9597947	0.9501527	0.9661673	0.9695291	0.94	1.0537658	0.9868
$${V}_{77}$$ (pu)	0.974911	1.001713	0.9959514	0.9899145	0.9620065	0.9915929	1.0049576	1.0502009	1.013
$${V}_{80}$$ (pu)	0.973564	1.001713	0.9991992	1.0058527	0.9687811	0.9999807	1.06	1.053419	1.0218
$${V}_{85}$$ (pu)	0.986859	1.001713	0.9994004	0.9954768	1.0131234	1.0010699	0.94	1.053422	1.0242
$${V}_{87}$$ (pu)	0.995449	1.001713	1.0461667	1.0036803	1.06	1.0306193	0.9941376	1.0511895	1.0432
$${V}_{89}$$ (pu)	0.988563	1.001713	1.0103991	1.024946	1.0197158	1.0231108	0.94	1.0535838	1.0274
$${V}_{90}$$ (pu)	0.989681	1.001713	1.0079355	0.9857261	1.0484171	1.0058755	1.0171342	1.0535094	1.0062
$${V}_{91}$$ (pu)	1.00143	1.001713	0.9898406	1.002056	1.06	1.0020844	1.0579789	1.0537953	1.0074
$${V}_{92}$$ (pu)	0.982589	1.001713	0.9837033	0.9957563	0.9657501	0.9990564	0.9658267	1.0500917	1.0153
$${V}_{99}$$ (pu)	0.992646	1.001713	1.0045406	0.9986228	1.014638	0.9992424	1.0179871	1.0535517	1.0182
$${V}_{100}$$ (pu)	1.001404	1.001713	1.0062797	1.0074172	0.9623508	1.0131239	0.9928849	1.0519887	1.0187
$${V}_{103}$$ (pu)	1.006439	1.001713	1.0109474	1.0013357	0.9677742	1.0092176	0.9438118	1.0494301	1.0146
$${V}_{104}$$ (pu)	0.987071	1.001713	0.9930689	0.9924871	1.0347805	1.0049059	0.997348	1.0536568	1.0067
$${V}_{105}$$ (pu)	0.988016	1.001713	0.9923136	0.9982727	1.06	1.0054728	1.0293715	1.0537466	1.0063
$${V}_{107}$$ (pu)	0.959298	1.001713	0.980022	1.0041706	1.0370517	1.0040741	1.06	1.0534344	0.9993
$${V}_{110}$$ (pu)	0.992368	1.001713	1.0012863	1.0171262	1.0508403	1.0088357	1.0134337	1.0505183	1.0147
$${V}_{111}$$ (pu)	0.981082	1.001713	1.0093074	1.0076076	0.983494	1.0052806	0.94	1.0506049	1.0247
$${V}_{112}$$ (pu)	0.992586	1.001713	1.0118551	1.0271379	1.017392	1.0175045	1.06	1.053739	1.0046
$${V}_{113}$$ (pu)	0.977017	1.001713	1.0343396	1.0090427	0.9927719	1.0010442	0.94	1.0521195	1.0057
$${V}_{116}$$ (pu)	0.99086	1.001713	0.9821816	0.9880097	0.9693682	1.0097187	0.94	1.0532565	1.0592
$${T}_{8}$$ (8–5)	0.988728	0.987085	1.0402999	0.9968856	1.0847371	0.9895942	1.0031343	0.9397173	1.0148
$${T}_{32}$$ (26–25)	0.9935602	1.017392	1.0116047	1.0109498	1.0121767	1.0008969	1.1	0.9993158	1.0978
$${T}_{36}$$ (30–17)	0.9654374	0.987126	0.9212424	1.0308447	1.0846095	1.005469	0.9206088	0.9991882	1.0348
$${T}_{51}$$ (38–37)	1.011300	0.980655	0.9773194	0.9989706	1.0960439	0.9862349	0.9	0.9902346	1.0107
$${T}_{93}$$ (63–59)	1.017721	0.980662	0.977828	1.0045998	1.079463	0.9942505	0.9171906	0.9508028	0.9946
$${T}_{95}$$ (64–61)	0.974791	0.980749	0.9704812	0.945104	1.0825113	0.9673068	0.9086571	0.9556937	1.0095
$${T}_{102}$$ (65–66)	0.945034	0.988361	0.9901509	0.9827035	1.0987101	0.9847707	1.0273736	0.9907212	0.9771
$${T}_{107}$$ (68–69)	0.946050	0.99266	0.9310724	0.9915445	1.0315702	0.9425921	1.0085836	0.9991271	0.9715
$${T}_{127}$$ (81–80)	0.98313	0.981881	0.9656016	1.0031895	0.9388147	0.9731689	0.9	0.9715853	1.0214
$${Q}_{34}$$ (MVAR)	10.83555	21.1929	6.8187763	15.778227	17.918252	13.791896	26.290058	14.655474	0.8808
$${Q}_{44}$$ (MVAR)	12.18013	19.07619	21.659563	11.082027	13.41777	7.2540701	8.8815734	10.516174	5.768
$${Q}_{45}$$ (MVAR)	7.836917	1.303665	15.489975	22.637872	24.056449	6.7121784	19.8684	15.802734	21.5888
$${Q}_{46}$$ (MVAR)	7.829812	24.64004	15.945289	13.111509	5.7751418	13.340721	4.9661793	13.734777	10.9322
$${Q}_{48}$$ (MVAR)	7.304086	1.9898	11.666063	9.8557041	4.1918716	13.132709	5.3761902	4.0110494	4.6786
$${Q}_{74}$$ (MVAR)	8.509035	17.56941	0.0285094	16.534195	7.1184334	15.380355	19.6847	8.4288615	24.2029
$${Q}_{79}$$ (MVAR)	9.962153	4.52566	4.8781181	12.43247	25.993238	15.329586	25.066682	12.658385	23.8787
$${Q}_{82}$$ (MVAR)	19.55525	19.43275	14.373142	9.6701052	3.7858894	19.335016	1.4871303	5.6523165	23.5807
$${Q}_{83}$$ (MVAR)	7.027822	13.91448	7.7951153	11.470982	20.036399	8.1539749	8.4579903	16.436302	20.4897
$${Q}_{105}$$ (MVAR)	12.34095	21.1684	14.461638	21.634179	11.179999	16.01292	11.586491	3.4548903	13.4731
$${Q}_{107}$$ (MVAR)	17.66833	1.404061	12.010001	5.1187294	3.4374936	11.949043	16.050517	13.731969	1.936
$${Q}_{110}$$ (MVAR)	21.95423	15.71943	23.761681	5.4946062	2.2205707	8.9900653	12.051309	3.9038993	18.1676
Fuel cost ($/h)	134,460.2	148,691	132,849.31	137,230.17	405,883.6	133,149.81	351,542.7	147,566.87	135,055.7
Power losses (MW)	66.6784	81.3435	82.76452	86.409369	138.11708	64.87534	112.24476	93.439966	60.9596
Voltage deviation (pu)	1.73006	0.47199	1.0684904	0.8930666	2.3370508	0.635191	2.4379931	2.8862895	1.0715
Iterations time (s)	232.8176	7260.92	578.900	484.923	191.44367	218.561	286.73577	282.9444	–

##### Case 2: real power losses reduction

**Figure 32:**
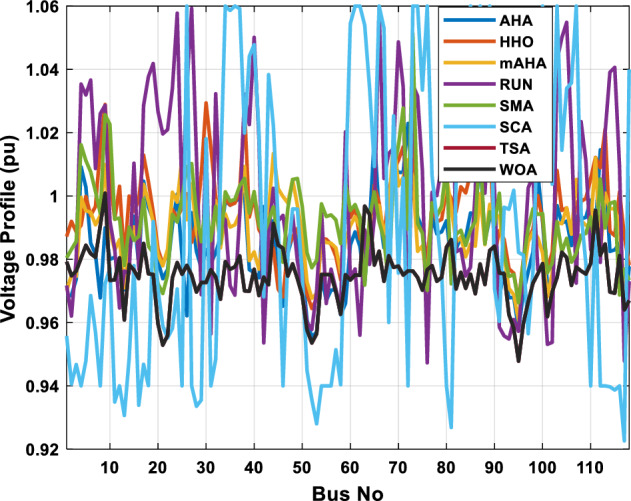
The voltage profile of all compared techniques for case 2.

**Figure 33:**
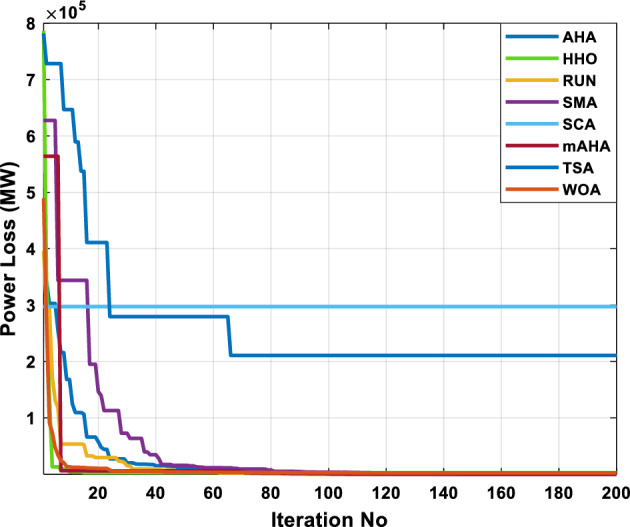
The convergence characteristics of the compared methodologies for case 2.

In this situation, active power loss reduction was the objective function. The results of using the mAHA method to arrive at the optimal solution are shown in Table 19. The mAHA algorithm effectively identifies the best control variable values that minimize system losses. As a result, real power losses dramatically dropped to 38.665089 MW when the mAHA algorithm was run without considering DG. Figure 32 illustrates the resilience and accuracy of the mAHA method by showing that the solution found using the mAHA algorithm isn’t violated at any bus, whereas other approaches are violated at multiple system load buses. Figure 33 shows the sharp convergence of real power losses based on the mAHA algorithm compared to other comparative methods. The mAHA method reaches the optimal result after only 20 iterations, demonstrating its rapid convergence. In order to evaluate the algorithm's efficiency, the estimated real power loss value is compared with that discovered using previously published population-based optimization techniques.

**Table 19:** Optimum control variables for the 118-bus network to minimize real power losses.

Control variables	AHA	HHO	mAHA	RUN	SCA	SMA	TSA	WOA
$${P}_{G1}$$ (MW)	41.09744	59.74255	63.17	37.98	90.32	47.44	100.00	83.17
$${P}_{G4}$$ (MW)	41.09744	59.74255	63.172718	37.980061	90.316133	47.443656	100	83.171278
$${P}_{G6}$$ (MW)	47.84307	21.52518	57.491813	13.362708	11.201364	60.889953	52.273491	85.980365
$${P}_{G8}$$ (MW)	49.89932	84.47667	74.261696	54.303465	85.335961	37.686452	16.181042	73.36141
$${P}_{G10}$$ (MW)	58.52498	46.50783	77.337508	54.332113	8.3625128	36.355284	22.50426	58.695314
$${P}_{G12}$$ (MW)	214.6012	495.6795	168.08393	101.20754	55.745619	194.09822	277.44419	44.084792
$${P}_{G15}$$ (MW)	102.2998	58.59224	72.48291	38.020623	54.733871	91.567149	107.21191	65.308488
$${P}_{G18}$$ (MW)	57.76874	31.13825	43.44979	65.582164	78.420903	54.791253	15.267232	41.46593
$${P}_{G19}$$ (MW)	43.6708	90.12134	92.409751	64.957887	99.822296	53.756684	100	39.682447
$${P}_{G24}$$ (MW)	57.38808	90.11533	40.674427	81.839344	20.380898	44.317142	89.658839	72.767921
$${P}_{G25}$$ (MW)	46.31976	52.31152	15.439714	49.144851	12.68231	40.917412	46.239235	44.336996
$${P}_{G26}$$ (MW)	198.188	288.7067	7.4288955	79.60956	169.28265	112.75378	123.24735	240.91161
$${P}_{G27}$$ (MW)	86.11525	21.70912	209.51306	209.14929	314.97109	183.47274	130.63003	376.98694
$${P}_{G31}$$ (MW)	41.72772	30.36907	33.376303	34.007538	87.69837	37.100229	29.182795	75.149964
$${P}_{G32}$$ (MW)	48.89732	8.789654	35.344738	36.986135	17.211996	73.313221	60.303689	24.462744
$${P}_{G34}$$ (MW)	33.09475	76.9951	62.377823	52.803271	34.812933	46.113374	47.413738	33.088248
$${P}_{G36}$$ (MW)	36.11661	44.42565	19.202987	50.74172	9.2764751	44.640337	51.28412	64.283161
$${P}_{G40}$$ (MW)	55.03835	42.06323	42.740548	61.493089	91.802865	41.565538	7.4166589	53.685976
$${P}_{G42}$$ (MW)	52.62606	81.70955	62.208467	53.516244	56.518837	47.89522	39.215588	68.704225
$${P}_{G46}$$ (MW)	38.66401	51.06799	78.511763	68.057245	97.518954	53.100788	0	28.874252
$${P}_{G49}$$ (MW)	73.74259	58.35155	75.332059	42.717917	77.596126	51.439023	97.582475	62.528796
$${P}_{G54}$$ (MW)	133.0003	52.24583	119.96661	151.26992	44.689265	146.43137	88.471338	40.132252
$${P}_{G55}$$ (MW)	87.95175	58.34299	62.422521	62.183984	22.766697	77.37581	14.19033	146.01869
$${P}_{G56}$$ (MW)	39.38335	86.21964	57.82063	43.387243	11.035996	42.774473	66.804547	54.740183
$${P}_{G59}$$ (MW)	91.23546	87.40985	52.575626	75.347659	14.548502	47.3879	38.581114	89.576317
$${P}_{G61}$$ (MW)	92.28118	51.96846	144.46758	166.01133	103.40127	185.84414	100.41427	247.3101
$${P}_{G62}$$ (MW)	175.527	36.53468	159.12381	178.44353	196.70527	118.97763	118.55808	110.84883
$${P}_{G65}$$ (MW)	63.7203	70.87187	14.316027	69.694309	42.557032	62.560956	11.178487	57.260116
$${P}_{G66}$$ (MW)	203.2908	359.03	313.64995	187.02492	35.666357	243.81227	60.234786	116.66153
$${P}_{G69}$$ (MW)	261.3465	102.5846	180.31093	191.21578	140.97123	223.70142	479.52978	3.5775662
$${P}_{G70}$$ (MW)	42.66203	46.88184	69.633997	54.265976	44.527084	60.780872	11.600645	10.51073
$${P}_{G72}$$ (MW)	24.74032	77.20445	14.080602	43.967456	24.891563	43.191675	69.342825	97.554609
$${P}_{G73}$$ (MW)	33.37329	11.24843	50.55066	56.072676	90.258448	40.03929	36.821557	95.740641
$${P}_{G74}$$ (MW)	80.49992	76.66897	58.692427	25.633044	87.634461	51.898618	49.603811	30.004697
$${P}_{G76}$$ (MW)	66.94796	67.92266	71.990951	46.434509	32.897239	60.666138	31.903279	77.056247
$${P}_{G77}$$ (MW)	53.60609	62.86581	75.058607	49.685957	69.39788	46.693944	45.408533	51.888519
$${P}_{G80}$$ (MW)	185.3136	176.0389	228.69239	192.79653	254.787	167.77276	290.08756	404.80289
$${P}_{G85}$$ (MW)	5.80E+01	52.23407	6.12E+01	3.94E+01	1.90E+01	4.46E+01	2.16E+01	4.89E+01
$${P}_{G87}$$ (MW)	30.07959	64.11637	47.04087	48.603442	40.910231	25.138855	83.304803	30.025381
$${P}_{G89}$$ (MW)	241.4598	215.1706	143.119	147.73457	134.13281	206.55055	248.72017	0
$${P}_{G90}$$ (MW)	58.31135	88.38945	67.386973	55.930857	46.28634	67.563113	5.3934767	30.595194
$${P}_{G91}$$ (MW)	45.21376	17.76906	35.130367	56.704564	18.424831	30.145731	81.537316	36.009804
$${P}_{G92}$$ (MW)	66.66457	34.8756	52.570306	60.661241	1.4705397	35.940967	31.741438	99.285385
$${P}_{G99}$$ (MW)	21.64804	77.29646	74.477714	60.50717	96.112171	49.700483	18.329325	80.061811
$${P}_{G100}$$ (MW)	148.9574	128.8383	175.36633	134.99464	191.03498	131.74867	283.01184	287.54204
$${P}_{G103}$$ (MW)	61.21779	17.72478	97.157685	47.69146	130.63633	53.348911	118.69674	6.8151309
$${P}_{G104}$$ (MW)	56.30818	44.83468	48.829589	44.063365	14.676268	43.202218	51.406846	64.127148
$${P}_{G105}$$ (MW)	39.53555	77.41098	9.76017	64.894666	86.580332	69.676691	30.716654	2.3646758
$${P}_{G107}$$ (MW)	65.69146	37.41983	53.263222	63.921324	10.520788	35.01897	20.630975	41.528069
$${P}_{G110}$$ (MW)	57.46827	34.36874	49.252236	38.604655	65.195198	48.064898	23.607089	90.061647
$${P}_{G111}$$ (MW)	69.69424	122.5381	13.888427	20.622442	35.604716	61.682609	6.9767092	129.98599
$${P}_{G112}$$ (MW)	53.29181	89.12237	74.054154	47.911072	83.749708	38.284061	12.634025	23.481278
$${P}_{G113}$$ (MW)	60.26998	52.57856	52.253899	40.582374	25.625671	50.426267	51.689691	98.127637
$${P}_{G116}$$ (MW)	56.93041	17.66201	89.663977	55.914564	42.494197	47.773139	98.17709	0.4198402
$${V}_{1}$$ (pu)	0.968088	1.040097	0.9872024	0.9701459	0.971716	0.9804618	0.9557644	0.9792085
$${V}_{4}$$ (pu)	1.0092	1.04068	0.9907121	0.9995252	1.0353357	1.0161725	0.94	0.9803475
$${V}_{6}$$ (pu)	0.985098	1.040424	0.9938383	0.9948357	1.0366118	1.0076317	0.9685304	0.9817886
$${V}_{8}$$ (pu)	0.967808	1.040722	1.0152952	0.9939211	1.0260066	0.9944261	0.94	0.9906788
$${V}_{10}$$ (pu)	0.979963	1.04125	1.0063936	0.9888986	0.9921435	1.022579	0.9771641	0.9733282
$${V}_{12}$$ (pu)	0.977461	1.040505	1.0030565	0.9861841	0.96461	0.9930268	0.94	0.9804456
$${V}_{15}$$ (pu)	0.993561	1.040171	0.9951855	0.9902151	1.0103565	0.9860055	0.9780106	0.976571
$${V}_{18}$$ (pu)	0.996585	1.040485	1.0046075	0.9983572	1.0376623	0.9841367	0.9400883	0.9754205
$${V}_{19}$$ (pu)	0.992458	1.043316	0.9949892	0.9917706	1.0418366	0.9820288	0.9918962	0.9752078
$${V}_{24}$$ (pu)	1.000587	1.040277	0.992984	0.9980691	1.0577448	0.9917825	0.9706126	0.9789591
$${V}_{25}$$ (pu)	0.980986	1.040968	0.9964482	1.0122064	0.9985354	0.9989998	0.94	0.9752822
$${V}_{26}$$ (pu)	0.962157	1.040508	1.0006607	0.9925215	1.0344248	1.0092218	1.06	0.9780409
$${V}_{27}$$ (pu)	0.99471	1.040592	0.9853642	1.0038834	1.0592673	1.0102285	0.94	0.9750847
$${V}_{31}$$ (pu)	0.978874	1.040095	1.0153098	0.9806748	0.9563978	0.9858719	0.94	0.9766888
$${V}_{32}$$ (pu)	0.982009	1.039831	0.9997346	0.9985073	1.0322982	0.9966994	0.9483897	0.974392
$${V}_{34}$$ (pu)	0.998504	1.040342	0.9992596	0.9943234	0.9924162	1.001309	1.06	0.9792355
$${V}_{36}$$ (pu)	0.998231	1.041005	0.9977514	0.9908821	0.9686749	0.9989457	1.06	0.9776215
$${V}_{40}$$ (pu)	0.975791	1.043366	0.9996034	0.9823235	1.0501138	1.0013734	1.0478049	0.9768293
$${V}_{42}$$ (pu)	0.996622	1.041116	0.9867889	0.9880911	0.9535843	0.9876344	0.9681381	0.9743456
$${V}_{46}$$ (pu)	0.965202	1.040586	0.9681231	0.9998156	0.9938655	0.9790445	0.94	0.9834584
$${V}_{49}$$ (pu)	0.987411	1.040651	0.9897257	0.9969909	0.9794672	1.0055318	0.9958435	0.9738927
$${V}_{54}$$ (pu)	0.974672	1.040055	0.9865499	0.9866225	0.9957565	0.997504	0.94	0.97518
$${V}_{55}$$ (pu)	0.966971	1.040464	0.986347	0.985304	0.9660327	0.9934965	0.94	0.9751778
$${V}_{56}$$ (pu)	0.970256	1.040399	0.9847159	0.9850591	0.9768786	0.9940236	0.94	0.9750922
$${V}_{59}$$ (pu)	0.966053	1.04053	1.001031	0.9858844	1.0202532	1.0043658	1.0027036	0.975102
$${V}_{61}$$ (pu)	0.988644	1.040439	0.996254	0.9934096	0.9769204	0.9972773	1.06	0.9750779
$${V}_{62}$$ (pu)	0.984075	1.04014	0.9864663	0.9901234	0.9560179	0.9933191	1.06	0.9749118
$${V}_{65}$$ (pu)	1.018847	1.040846	1.0034682	0.9940511	0.9930475	1.0012935	1.0093315	0.9781872
$${V}_{66}$$ (pu)	0.994724	1.040703	0.9978156	0.9970233	1.0593184	0.9918188	1.06	0.9825414
$${V}_{69}$$ (pu)	1.01017	1.040661	1.0079273	1.0192065	1.0150269	1.0088762	1.0253148	0.9774504
$${V}_{70}$$ (pu)	1.007457	1.0408	1.0112365	1.0031896	1.0486669	1.0157169	0.9756345	0.9778426
$${V}_{72}$$ (pu)	1.022917	1.040245	1.0022984	1.011444	0.951202	0.9894925	0.94	0.9763378
$${V}_{73}$$ (pu)	1.001859	1.040693	1.0219807	1.0059221	1.03516	1.0512486	1.06	0.9763157
$${V}_{74}$$ (pu)	0.99341	1.040368	0.9904243	0.9825074	1.0315027	0.9912699	1.06	0.974259
$${V}_{76}$$ (pu)	0.977451	1.040408	0.9791306	0.9721953	0.947284	0.9701926	1.06	0.9766917
$${V}_{77}$$ (pu)	0.99346	1.040769	0.997122	0.9983206	1.0011486	0.9978674	1.0064146	0.9779984
$${V}_{80}$$ (pu)	0.992155	1.040292	1.0019669	1.006187	1.0383178	1.0102281	0.94	0.9838288
$${V}_{85}$$ (pu)	9.95E−01	1.040414	1.0070851	0.9848153	0.977469	0.9891926	1.06	0.9752725
$${V}_{87}$$ (pu)	1.012476	1.040959	1.0197444	1.0006733	1.0590954	1.0161355	1.06	0.9784978
$${V}_{89}$$ (pu)	0.993084	1.042364	1.0204971	0.9973551	1.0296704	0.9949916	1.06	0.9827812
$${V}_{90}$$ (pu)	0.998656	1.040724	0.9811065	0.9936947	1.001934	0.9908913	0.9707896	0.9841443
$${V}_{91}$$ (pu)	0.968545	1.040758	0.9890702	0.9937463	0.9585281	1.0008729	0.9604145	0.9756531
$${V}_{92}$$ (pu)	0.975804	1.040642	0.9863025	0.9808231	0.9557864	0.9815501	0.9964343	0.9754205
$${V}_{99}$$ (pu)	1.007259	1.040946	1.0001286	0.9868556	0.976685	0.9942095	1.0245109	0.9754205
$${V}_{100}$$ (pu)	0.991788	1.040484	0.992514	0.9818817	0.9779302	0.994412	1.06	0.9786301
$${V}_{103}$$ (pu)	0.996966	1.041128	1.0080675	0.9916241	1.0428573	0.9918399	1.06	0.974224
$${V}_{104}$$ (pu)	0.991494	1.039952	0.9954371	0.9912491	1.0500049	0.9823495	1.0144579	0.9822001
$${V}_{105}$$ (pu)	0.992044	1.043293	0.9962123	0.9962186	1.0548573	0.9866123	1.0339973	0.9805475
$${V}_{107}$$ (pu)	0.993576	1.039937	0.9981234	1.0052099	0.9724655	0.9819421	1.06	0.9775202
$${V}_{110}$$ (pu)	0.998705	1.040898	1.0055254	0.990373	0.9830757	0.9933525	0.94	0.9787194
$${V}_{111}$$ (pu)	0.989706	1.040911	1.0121748	1.0120406	0.9802301	0.9831952	0.9956676	0.995487
$${V}_{112}$$ (pu)	1.014581	1.040876	1.008924	0.9817246	0.9790422	1.0053422	0.94	0.9812771
$${V}_{113}$$ (pu)	1.006955	1.040683	1.0211005	1.0008882	1.0199789	0.9899072	0.94	0.9848137
$${V}_{116}$$ (pu)	0.984992	1.040588	1.0013401	0.9907347	1.0092763	0.9687299	0.94	0.9811649
$${T}_{8}$$ (8–5)	0.961941	0.994941	0.976085	1.0030619	0.9744789	0.9841297	0.9693279	0.9812456
$${T}_{32}$$ (26–25)	1.003984	0.998583	1.0080821	0.9687052	0.9559941	1.0243936	1.1	0.9970697
$${T}_{36}$$ (30–17)	0.961158	1.004614	1.0109428	0.9751361	0.9490533	0.9661764	1.0914774	0.9700844
$${T}_{51}$$ (38–37)	0.986757	0.983608	1.0120484	1.0043059	1.0411014	0.9892148	0.9536769	0.9739014
$${T}_{93}$$ (63–59)	0.974525	0.98233	0.9981499	0.9860788	0.9967467	0.9330803	1.1	1.0253342
$${T}_{95}$$ (64–61)	0.994496	0.983065	0.9620037	0.9933837	0.9703208	1.0104946	0.9680823	1.0235902
$${T}_{102}$$ (65–66)	1.029313	0.982812	1.0021577	0.9748996	0.9085976	1.0104529	0.9395403	0.9685103
$${T}_{107}$$ (68–69)	1.035063	0.982848	0.968715	0.9693324	0.9716401	0.900029	1.0424151	0.9731361
$${T}_{127}$$ (81–80)	0.947719	0.999442	0.9786187	0.9816657	1.0944949	0.953104	0.9178263	0.994653
$${Q}_{34}$$ (MVAR)	19.28933	27.03047	13.955289	20.920446	14.340599	10.848257	0	8.0849357
$${Q}_{44}$$ (MVAR)	18.48367	3.536302	11.56354	17.530187	15.374624	11.474634	24.862788	15.82778
$${Q}_{45}$$ (MVAR)	9.487523	26.70218	5.5065409	20.584897	19.548614	10.380552	29.877974	22.53874
$${Q}_{46}$$ (MVAR)	12.43272	27.03047	14.837992	16.029212	20.699225	6.8288169	21.47262	0
$${Q}_{48}$$ (MVAR)	11.8527	4.308818	5.1092206	19.370188	28.982478	13.374493	30	7.2203092
$${Q}_{74}$$ (MVAR)	15.74131	23.21207	18.117852	14.146679	1.7113708	11.027649	5.6625602	12.543888
$${Q}_{79}$$ (MVAR)	18.54146	4.581923	10.750229	15.921808	17.298592	14.183762	6.9977644	14.663364
$${Q}_{82}$$ (MVAR)	20.00827	24.69894	19.683528	16.734658	4.5885868	17.714973	1.5699657	17.886835
$${Q}_{83}$$ (MVAR)	15.67621	4.502895	25.937304	15.573896	13.807475	12.173914	26.597532	19.14965
$${Q}_{105}$$ (MVAR)	12.69853	13.86229	15.527258	13.392902	27.389885	15.225864	10.712261	3.1445637
$${Q}_{107}$$ (MVAR)	12.80924	22.21036	11.46459	10.544771	11.72039	12.814664	17.844323	17.618232
$${Q}_{110}$$ (MVAR)	8.547344	22.1479	13.53298	19.133512	18.74049	11.763885	15.017109	13.082735
Fuel cost ($/h)	148,360	3174.691	150,850.00	147,790.00	154,810.00	147,440.00	166,800.00	162,180.00
Power losses (MW)	53.42718	88.78305	38.665089	45.120461	124.44097	47.333447	111.54349	100.08438
Voltage deviation (pu)	1.233117	2.195552	0.8621513	0.970505	1.4848662	0.8785978	2.4662778	1.8042842
Iterations time (s)	183.9421	4975.840	594.122	420.4121	186.191	290.5742	179.5363	261.926

##### Case 3: voltage deviation minimization

**Figure 34:**
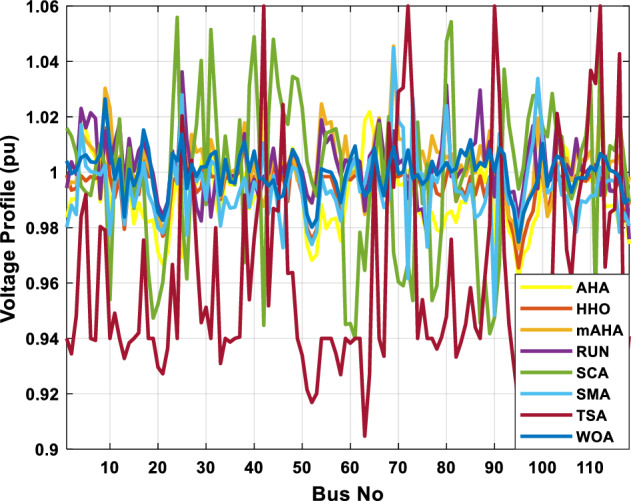
The voltage profile of the mAHA and other compared algorithms for case 3.

**Figure 35:**
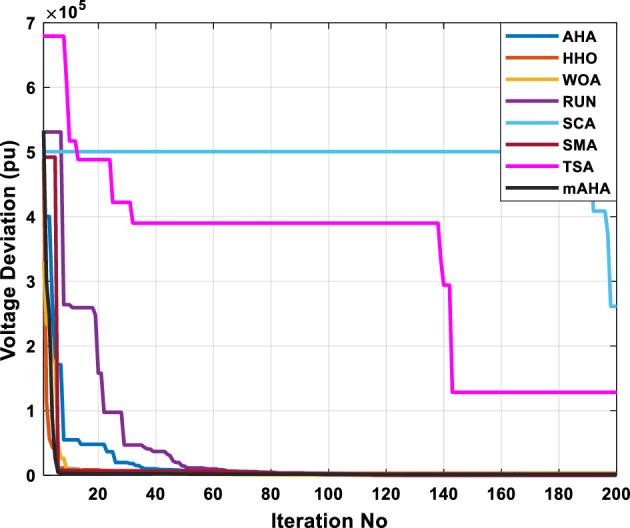
The convergence characteristics of mAHA and other compared algorithms for case 3.

Voltage deviation is chosen as the target function to be improved using the mAHA algorithm to improve the voltage profile. Figure 34 illustrates that, unlike other algorithms, the mAHA algorithm could maintain the allowed voltage constraints. Figure 35 shows the trend of decreasing system voltage deviation. Table 20 presents the findings. The results show that when employing the mAHA method, the voltage deviation index is 0.4264959 pu. Table 18 compares solutions achieved using the mAHA method and other population-based optimization techniques, with the former yielding superior results.

**Table 20:** Optimum control variables for the 118-bus network to optimize voltage deviation.

Control variables	AHA	HHO	mAHA	RUN	SCA	SMA	TSA	WOA
$${P}_{G1}$$ (MW)	52.82	86.53	58.52	84.95	95.93	40.46	21.95	18.13
$${P}_{G4}$$ (MW)	52.822532	86.534929	58.515167	84.9455	95.933873	40.463808	21.948832	18.125984
$${P}_{G6}$$ (MW)	46.7889	8.4559332	8.1777681	39.14065	0.5177518	33.998707	65.419773	31.439515
$${P}_{G8}$$ (MW)	72.443451	29.739024	69.4858	60.493318	3.8944833	61.89915	61.622648	81.536623
$${P}_{G10}$$ (MW)	44.742481	39.914872	69.647015	51.947028	88.442935	54.08992	29.456074	18.384239
$${P}_{G12}$$ (MW)	254.94598	176.40549	121.13453	78.437014	29.266196	309.56541	10.050499	131.17817
$${P}_{G15}$$ (MW)	100.87837	7.9782063	45.706402	49.262106	12.116081	71.753132	48.770883	88.116755
$${P}_{G18}$$ (MW)	36.108738	83.157416	18.581217	41.530358	58.100739	42.412301	28.273554	39.598782
$${P}_{G19}$$ (MW)	66.847449	50.520024	98.726322	73.742826	47.697574	22.493054	7.8748671	65.529929
$${P}_{G24}$$ (MW)	65.156197	28.782801	69.168917	47.047217	34.120001	60.344564	32.125257	40.938533
$${P}_{G25}$$ (MW)	28.683409	18.118969	1.8470755	83.219766	51.369075	40.390541	33.372819	13.015991
$${P}_{G26}$$ (MW)	109.21034	107.13653	122.15009	113.03392	71.074438	185.82211	101.36871	102.30161
$${P}_{G27}$$ (MW)	251.14927	372.52551	35.50038	245.47584	45.40322	213.76515	239.72192	335.95466
$${P}_{G31}$$ (MW)	76.399316	4.1003473	97.619138	62.996597	75.224936	34.368899	31.640831	31.479638
$${P}_{G32}$$ (MW)	64.61721	50.510284	80.022305	35.998888	50.210404	45.10058	105.5269	28.733001
$${P}_{G34}$$ (MW)	54.367909	54.481789	73.698048	74.517845	12.648857	18.355511	21.250825	49.913331
$${P}_{G36}$$ (MW)	64.739915	33.335546	87.218256	46.58226	64.786678	91.595118	17.974935	59.268433
$${P}_{G40}$$ (MW)	65.495945	5.1947102	11.373845	52.247796	67.981499	52.74163	70.318428	6.0074085
$${P}_{G42}$$ (MW)	46.524691	52.820251	51.484888	45.519705	51.559365	46.915288	11.719574	41.37987
$${P}_{G46}$$ (MW)	28.54616	10.10687	85.625471	53.238145	20.399013	49.712917	14.304283	65.535454
$${P}_{G49}$$ (MW)	85.026877	4.5589597	118.91603	33.526012	10.288305	61.268908	70.943099	70.342941
$${P}_{G54}$$ (MW)	168.79389	85.08324	229.38704	137.71746	296.80871	189.57607	14.611018	129.14709
$${P}_{G55}$$ (MW)	68.372873	67.797872	85.68927	17.482681	144.70944	66.916912	119.95778	22.049981
$${P}_{G56}$$ (MW)	21.71997	7.5726755	19.245097	29.391758	11.519586	55.035735	6.6625432	21.330834
$${P}_{G59}$$ (MW)	34.864823	80.995542	98.119919	61.282769	82.811177	47.185611	40.407276	47.543443
$${P}_{G61}$$ (MW)	151.98909	63.877465	195.13156	139.75611	18.289501	112.37041	255	228.71916
$${P}_{G62}$$ (MW)	60.881268	20.146075	153.88487	107.91081	238.30156	104.14495	58.067228	144.74258
$${P}_{G65}$$ (MW)	9.4218251	20.131163	39.948874	59.066935	35.328085	13.496392	30.263897	41.463265
$${P}_{G66}$$ (MW)	109.04643	322.91442	294.81284	112.05428	251.16337	147.04903	411.54761	359.13276
$${P}_{G69}$$ (MW)	70.740547	20.308617	146.2685	260.02602	70.257549	255.82805	21.03211	137.06534
$${P}_{G70}$$ (MW)	43.901337	14.30852	56.722197	22.367049	54.48107	61.867748	13.013025	29.649725
$${P}_{G72}$$ (MW)	70.289334	24.155251	99.929436	62.17154	35.671253	42.765717	100	31.066932
$${P}_{G73}$$ (MW)	19.761606	85.387951	50.354984	33.959724	95.425152	24.475638	100	57.652867
$${P}_{G74}$$ (MW)	80.407997	64.387694	98.496524	57.007128	2.2893454	62.03897	18.124487	46.926745
$${P}_{G76}$$ (MW)	97.136392	88.843241	96.510912	96.255039	92.000541	96.814903	100	55.777601
$${P}_{G77}$$ (MW)	54.464412	22.065468	41.049907	61.237556	39.37593	54.233358	93.404263	53.249031
$${P}_{G80}$$ (MW)	338.45338	358.29594	49.764713	283.21856	567.49675	183.60211	422.52689	118.7305
$${P}_{G85}$$ (MW)	2.62E+01	9.09E+01	5.22E+01	3.62E+01	3.20E+01	3.57E+01	3.22E+01	2.50E+01
$${P}_{G87}$$ (MW)	44.659989	57.054647	15.374826	56.358788	28.223967	16.733744	39.974101	52.714595
$${P}_{G89}$$ (MW)	273.42509	196.89184	63.20857	193.36092	295.43476	294.40581	447.25844	306.83683
$${P}_{G90}$$ (MW)	25.860891	4.6699173	67.998268	65.232943	17.183073	39.27474	4.0527752	22.828486
$${P}_{G91}$$ (MW)	50.393756	41.176445	93.53563	49.838634	90.351806	44.173997	67.990342	24.226635
$${P}_{G92}$$ (MW)	46.064114	88.734637	72.349485	42.203932	2.3380964	28.483208	14.626604	23.480601
$${P}_{G99}$$ (MW)	76.449496	86.403102	8.7029995	65.671936	61.216134	35.562851	23.802229	18.429791
$${P}_{G100}$$ (MW)	196.80331	284.74711	303.31712	159.24444	111.37319	162.19673	153.11885	194.63035
$${P}_{G103}$$ (MW)	109.63725	132.79272	108.01333	67.123699	41.852447	69.831514	111.43613	112.04441
$${P}_{G104}$$ (MW)	64.407691	48.91815	67.727818	40.808973	20.504444	70.646135	23.428013	74.158657
$${P}_{G105}$$ (MW)	46.701177	7.4280396	73.881827	63.157171	81.982191	35.893146	43.60918	42.079564
$${P}_{G107}$$ (MW)	59.53491	48.971405	21.425748	54.454785	26.370647	45.431396	16.917964	18.175037
$${P}_{G110}$$ (MW)	35.060333	88.039801	83.92072	42.870323	25.222161	50.166939	22.799808	57.450984
$${P}_{G111}$$ (MW)	27.822147	4.7383986	79.914024	45.385803	11.901008	55.136908	90.84821	53.407116
$${P}_{G112}$$ (MW)	24.049655	30.522041	42.18986	40.976413	38.143633	47.001754	37.008763	24.919959
$${P}_{G113}$$ (MW)	12.880797	37.042488	2.1130705	37.209325	27.793511	87.359959	57.751145	22.890486
$${P}_{G116}$$ (MW)	48.9738	49.368026	58.801534	90.015056	4.0243278	36.829394	2.0638751	19.614222
$${V}_{1}$$ (pu)	0.9837169	0.9989332	1.0040714	0.9946496	1.0159518	0.9801384	0.94	0.9943254
$${V}_{4}$$ (pu)	1.0171959	0.9987023	1.0052552	1.0147383	0.9949769	1.0170704	0.983829	1.0230408
$${V}_{6}$$ (pu)	1.0083777	0.9984379	1.0037718	1.0093424	0.9915861	1.0002216	0.94	1.0214704
$${V}_{8}$$ (pu)	0.9893358	0.9984178	1.0063478	0.9999738	1.0101761	0.9990695	0.9803742	0.9904311
$${V}_{10}$$ (pu)	0.9858529	0.9984143	1.0095494	1.0232675	0.9539494	0.9774348	0.94	1.0032765
$${V}_{12}$$ (pu)	1.0025731	0.9984161	1.0047479	1.0056947	1.0192324	1.0011642	0.94	1.0177339
$${V}_{15}$$ (pu)	0.9814197	0.9986413	0.9951419	0.9981086	0.9869603	0.9875277	0.94	1.0003939
$${V}_{18}$$ (pu)	0.9821415	0.9984084	1.0085976	1.0020251	0.9638907	0.9920888	0.94	1.005803
$${V}_{19}$$ (pu)	0.9828388	0.9984333	0.9951357	0.9974385	0.9473387	0.9880534	0.94	0.999233
$${V}_{24}$$ (pu)	1.0002703	0.9984191	1.0041248	1.0041834	1.0559348	0.9958298	0.94	0.9965601
$${V}_{25}$$ (pu)	0.9693263	0.9984245	1.0137953	1.0001874	0.984345	1.0279515	1.0203475	1.0362323
$${V}_{26}$$ (pu)	0.9868014	0.9988742	0.9941351	0.9996746	0.9614965	0.9773494	0.9966932	1.0116661
$${V}_{27}$$ (pu)	0.9963548	0.9984343	1.0028144	1.0136335	1.0033783	1.0011873	1.004309	0.999399
$${V}_{31}$$ (pu)	1.0089892	0.9984143	1.0063028	1.0117597	1.0515308	1.0040872	0.94	0.9838394
$${V}_{32}$$ (pu)	0.9900412	0.9984248	1.0074586	1.004889	1.0221506	1.0025537	0.9799564	0.9970802
$${V}_{34}$$ (pu)	0.9992201	0.9984214	1.004583	1.0005623	1.0130548	0.9909856	0.94	1.004611
$${V}_{36}$$ (pu)	0.9955713	0.9984108	0.9961919	1.000895	0.9954976	0.9873726	0.94	0.9987836
$${V}_{40}$$ (pu)	0.9909765	0.9984272	1.0076372	1.0040013	1.0489144	0.9964579	0.9760751	0.9973915
$${V}_{42}$$ (pu)	1.0226928	0.9984107	0.9973069	0.9876911	0.9447065	1.0105486	1.06	0.9985096
$${V}_{46}$$ (pu)	1.0033952	0.9984333	0.9915431	1.0023346	1.0219538	0.972747	1.0244405	1.0017381
$${V}_{49}$$ (pu)	1.0046369	0.9984325	1.0049397	0.9970443	1.0335898	1.0007238	0.94	1.00432
$${V}_{54}$$ (pu)	0.9882552	0.9984143	1.0012355	1.0246685	1.0033544	0.9981182	0.94	1.0188926
$${V}_{55}$$ (pu)	0.9796473	0.9984087	1.0004274	1.0174276	1.0141796	0.9921352	0.94	1.0106656
$${V}_{56}$$ (pu)	0.982843	0.9984145	0.9999944	1.0182261	1.0148105	0.9933081	0.94	1.0132038
$${V}_{59}$$ (pu)	0.9884191	0.9984095	0.998566	1.0130436	0.9450411	0.9962512	0.94	1.0044433
$${V}_{61}$$ (pu)	1.003285	0.9989294	1.0094787	1.0004497	0.94	1.0051671	0.94	1.005104
$${V}_{62}$$ (pu)	0.99553	0.9988909	0.9972736	0.9971621	0.978322	0.9929546	0.94	1.0037656
$${V}_{65}$$ (pu)	1.013367	0.9984248	1.0087647	1.0123909	1.0006405	1.0069515	1.0045633	1.0085153
$${V}_{66}$$ (pu)	1.0088278	0.9985688	0.997576	1.0195755	1.0175064	0.9950526	0.94	1.0186962
$${V}_{69}$$ (pu)	1.0098445	0.9984135	1.0091532	1.0455458	0.9710221	1.0448585	0.9945533	1.0148784
$${V}_{70}$$ (pu)	0.9954592	0.9984138	0.9993955	1.0120441	0.9604082	1.0184288	1.0290654	1.0030583
$${V}_{72}$$ (pu)	1.0007014	0.9984236	1.0080069	0.9744352	0.9672843	0.9616424	1.06	0.9905373
$${V}_{73}$$ (pu)	0.9943003	0.9984269	0.9978239	1.0203373	0.9536638	1.026781	1.0229002	1.0081383
$${V}_{74}$$ (pu)	0.9861425	0.9987103	0.9991108	1.006167	1.0180586	1.0004575	0.94	0.9862239
$${V}_{76}$$ (pu)	0.9763866	0.9984097	0.9975045	1.0008878	1.0029675	0.9729707	0.94	0.9769082
$${V}_{77}$$ (pu)	0.9854762	0.9984191	1.0039189	1.0131573	0.9536917	0.998818	0.94	1.0014511
$${V}_{80}$$ (pu)	0.9847373	0.9984173	1.003484	1.0051557	1.0472714	1.0240386	0.9479476	1.0314414
$${V}_{85}$$ (pu)	0.9963885	0.9984133	1.0117173	1.0042518	1.011343	0.9956523	0.9583123	1.0089568
$${V}_{87}$$ (pu)	1.0022364	0.9986272	1.0035426	1.0095323	0.9456332	0.984938	0.94	1.0296648
$${V}_{89}$$ (pu)	1.0100382	0.9984278	1.0080348	1.0150081	0.9417234	0.9983099	0.9812302	1.0111174
$${V}_{90}$$ (pu)	0.9742785	0.9988189	1.0001894	0.9920549	0.947558	0.9483727	1.06	0.9911235
$${V}_{91}$$ (pu)	1.0044681	0.9988556	1.0088354	1.0128867	0.9665145	1.0139387	1.0286086	1.0091032
$${V}_{92}$$ (pu)	0.990492	0.9984068	1.0064261	0.9933333	1.0371885	0.9960263	0.9711364	0.9994063
$${V}_{99}$$ (pu)	0.984746	0.998423	0.9970975	1.019621	1.0279531	1.0338292	0.9622277	0.9940666
$${V}_{100}$$ (pu)	1.0024384	0.9984352	1.0103693	0.9998407	1.0161768	1.0123685	0.94	1.0165398
$${V}_{103}$$ (pu)	1.0108807	0.9984246	1.0058325	1.0013786	1.0128002	1.0025423	1.0212102	1.0206343
$${V}_{104}$$ (pu)	1.010391	0.9984311	1.0046151	1.0045754	1.0129173	0.9916926	1.0088001	1.0032501
$${V}_{105}$$ (pu)	1.0082642	0.9989441	0.9992869	1.0050659	0.9944849	0.9914421	0.9719043	1.0027299
$${V}_{107}$$ (pu)	0.9961495	0.9988312	0.9977614	1.0073907	0.9960014	0.9784303	0.9866448	1.0120958
$${V}_{110}$$ (pu)	0.9969265	0.9989334	1.0020228	1.0075833	1.0315338	0.9914322	1.03678	1.0018077
$${V}_{111}$$ (pu)	1.0030909	0.9984206	1.0005508	0.9962088	0.954251	0.9915563	1.0320566	1.0025809
$${V}_{112}$$ (pu)	0.9974033	0.9984158	1.006686	1.0278147	1.0472102	1.001725	1.06	0.9917132
$${V}_{113}$$ (pu)	0.9874671	0.9984394	1.0050653	0.9916138	0.9926523	0.996391	0.965744	1.0059397
$${V}_{116}$$ (pu)	0.9797036	0.9984283	0.9979973	1.0059574	1.0182969	0.9783232	1.0427597	0.9993608
$${T}_{8}$$ (8–5)	0.9646251	1.0073863	0.9909622	0.9894006	1.0329934	1.0421873	0.9133254	0.9931396
$${T}_{32}$$ (26–25)	1.0507266	1.0083841	0.9944273	1.0357593	1.0281805	0.9842488	0.9742704	0.9737324
$${T}_{36}$$ (30–17)	1.0441545	0.9897431	0.9527715	0.96343	0.9759361	0.9614697	0.9	0.9798013
$${T}_{51}$$ (38–37)	0.9675287	1.006082	0.9898559	1.0076214	0.9228551	0.9887479	1.0741438	0.9963579
$${T}_{93}$$ (63–59)	1.0265237	0.989855	0.9879769	0.9547485	0.9744587	0.9859937	0.9255106	0.9486617
$${T}_{95}$$ (64–61)	1.0279707	1.0066901	0.9675022	0.9932502	1.0774062	1.0033831	0.9465455	1.0191361
$${T}_{102}$$ (65–66)	0.9868859	0.9878702	0.988658	0.9838461	0.9	0.9933303	1.089632	0.9780493
$${T}_{107}$$ (68–69)	0.976303	1.0091634	0.9944131	0.9976022	1.0153946	0.9644926	0.9	0.9688566
$${T}_{127}$$ (81–80)	0.9801705	0.9985024	0.9791968	0.9761716	1.05387	0.9827831	0.9426853	0.9507009
$${Q}_{34}$$ (MVAR)	13.414654	11.508929	3.8936043	10.133201	20.549395	14.796015	8.4267986	9.7466545
$${Q}_{44}$$ (MVAR)	13.226379	10.875672	6.6314131	12.189323	29.907192	17.418497	13.620993	15.765508
$${Q}_{45}$$ (MVAR)	8.3579497	15.414236	13.565095	13.702685	20.334043	15.08956	24.974683	4.4859842
$${Q}_{46}$$ (MVAR)	13.860582	7.9251187	20.930132	9.0197959	20.2594	15.830149	27.136152	7.9177454
$${Q}_{48}$$ (MVAR)	10.464785	25.229334	14.90824	20.695533	9.241588	17.687591	15.737777	9.3538359
$${Q}_{74}$$ (MVAR)	14.679007	27.999099	22.236306	13.895714	10.059794	14.299266	18.367341	5.9104357
$${Q}_{79}$$ (MVAR)	8.8558048	7.7045075	23.556863	21.378317	14.184079	15.710789	30	20.972436
$${Q}_{82}$$ (MVAR)	13.041468	18.992988	24.446815	15.400184	16.977218	16.488823	14.261494	10.13504
$${Q}_{83}$$ (MVAR)	25.539978	25.273233	20.906037	19.063106	10.862795	8.1012615	16.847727	10.437319
$${Q}_{105}$$ (MVAR)	17.153076	14.202801	26.429986	5.7080354	5.7350681	2.8423988	14.49448	17.289959
$${Q}_{107}$$ (MVAR)	14.373873	8.6060195	12.35709	16.736318	15.991045	21.61781	21.622876	12.139966
$${Q}_{110}$$ (MVAR)	16.793366	25.18489	26.728857	16.698825	21.185935	8.4194362	15.993423	17.490426
Fuel cost ($/h)	154,450.00	155,710.00	166,130.00	150,250.00	153,300.00	143,650.00	167,490.00	148,630.00
Power losses (MW)	73.731774	104.54867	54.434108	61.367125	113.36205	70.485916	150.1514	66.368218
Voltage deviation (pu)	0.8664091	0.6341614	0.4264959	0.4813462	1.1283125	0.609969	3.2328398	0.4621417
Iterations time (s)	186.96055	4605.978	658.75	388.9675	184.912	264.998	177.3198	244.5468

##### Case 4: lessening of several objective functions devoid of emissions

**Figure 36:**
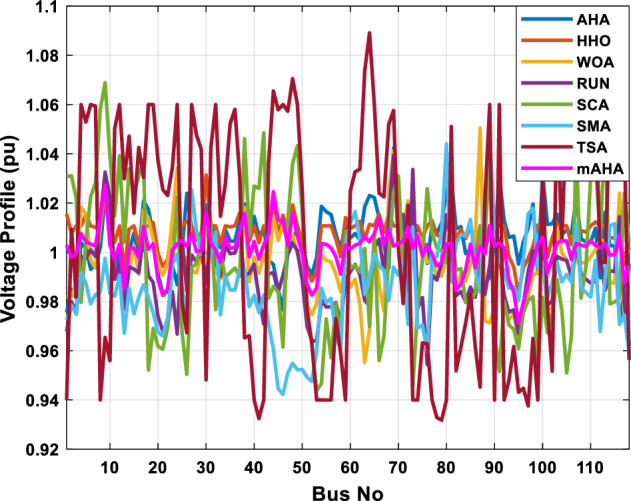
The voltage profile of the compared techniques for case 5.

**Figure 37:**
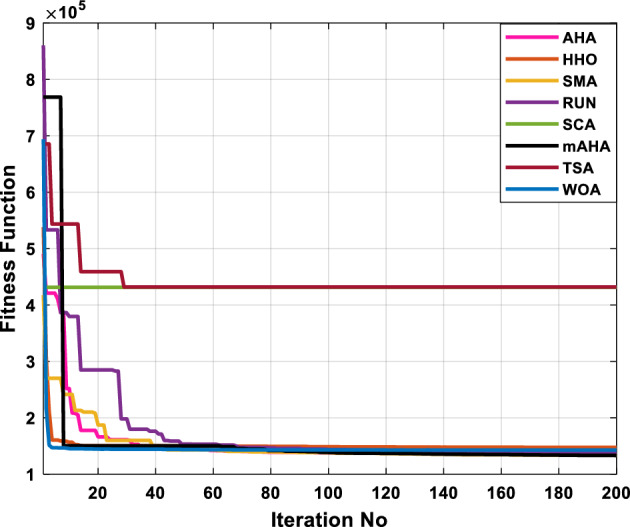
The convergence characteristics of all methodologies for case 5.

**Figure 38:**
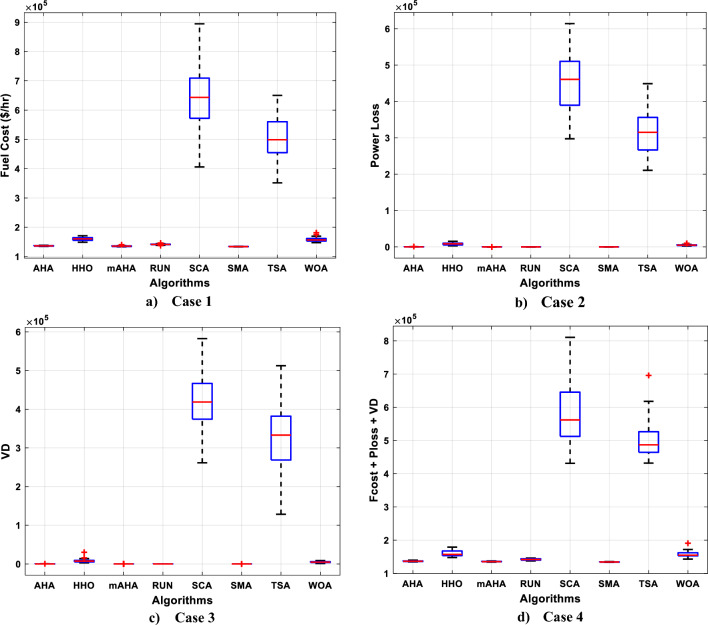
The boxplot of mAHA and other compared algorithms for IEEE 118 bus network.

In order to obtain the full benefits of the planned test system, a multi-objective function minimizes fuel operational cost, transmission power loss, and voltage-level deviation is implemented. According to Table 21, the multi-objective OPF issue was tackled by using mAHA in conjunction with other comparative algorithms without considering emissions. Several OF problems can be solved more economically by adopting mAHA than other comparable algorithms. As a result, the total objective function with 133,257.99 $/h based on mAHA technique outperforms all other algorithms with 134,581.11 $/h, 147,663.18 $/h, 137,402.63 $/h, 431,355.38 $/h, 133,921.61 $/h, 431,849.5 $/h and 143,003.58 $/h achieved by AHA, HHO, RUN, SCA, SMA, TSA, and WOA, respectively. All voltage profiles are within the specified limits except for the TSA algorithm, as illustrated in Fig. 36. Furthermore, mAHA still demonstrates quick and smooth convergence characteristics, as seen in Fig. 37. Based on the proposed mAHA algorithm, the boxplots in Fig. 38 display the lowest values for fuel cost, real power losses, and total voltage deviation. As illustrated previously, the boxplots of the proposed mAHA show a high degree of susceptibility to reducing the cost function with the lowest values.

**Table 21:** Optimum control variables for the 118-bus grid to optimize the multi-objective function.

Control variables	AHA	HHO	mAHA	RUN	SCA	SMA	TSA	WOA	MJAYA^67^
$${P}_{G1}$$ (MW)	18.07	72.32	40.66	40.46	19.31	40.47	33.42	19.48	50.23
$${P}_{G4}$$ (MW)	18.071783	72.31595	40.660591	40.457111	19.306794	40.471011	33.424649	19.483865	4.81
$${P}_{G6}$$ (MW)	44.017731	1.3831367	21.161305	29.748183	28.931331	40.027392	17.045858	70.156137	64.46
$${P}_{G8}$$ (MW)	23.028437	81.752214	56.376741	19.026106	28.86892	10.923141	36.726754	39.982318	3.11
$${P}_{G10}$$ (MW)	50.231	1.3969462	22.048955	30.930415	78.949703	8.6516309	43.33107	20.321959	167.05
$${P}_{G12}$$ (MW)	283.31041	415.21215	310.44926	327.00309	73.558177	348.01603	92.220054	127.19647	53.18
$${P}_{G15}$$ (MW)	75.832547	113.12214	75.735244	93.401035	110.43887	65.671559	68.455398	110.32746	34.90
$${P}_{G18}$$ (MW)	34.478531	75.164004	41.036352	72.06681	66.207553	41.286114	22.448149	69.978491	23.65
$${P}_{G19}$$ (MW)	47.828237	10.979219	47.061548	37.051758	43.1937	38.342663	40.74003	6.8214424	71.86
$${P}_{G24}$$ (MW)	42.169521	66.963842	23.310636	57.427369	39.387832	35.712421	12.443041	57.700429	33.08
$${P}_{G25}$$ (MW)	35.954214	69.730894	0.0654419	30.369156	27.861816	24.80807	85.12492	62.282004	200.06
$${P}_{G26}$$ (MW)	142.32751	113.92602	129.4088	160.38569	220.6471	179.92375	258.24186	126.31629	207.52
$${P}_{G27}$$ (MW)	225.49923	69.067183	226.09857	83.586667	143.45843	156.85726	256.05459	201.30856	25.87
$${P}_{G31}$$ (MW)	30.428814	38.968158	36.673222	57.004756	50.587049	31.358738	66.319929	30.224141	10.65
$${P}_{G32}$$ (MW)	9.7118704	1.4478344	7.1768536	7.2451877	40.627324	7.7847454	48.558999	7.6722874	76.65
$${P}_{G34}$$ (MW)	10.154722	73.567255	5.3586903	29.325702	31.317443	44.768643	100	38.491182	61.91
$${P}_{G36}$$ (MW)	41.634698	31.871083	51.2431	35.79511	40.424286	50.022292	45.200328	2.1616249	33.29
$${P}_{G40}$$ (MW)	41.492364	3.2768603	37.302258	38.516879	79.210986	4.9493626	100	1.8804106	58.63
$${P}_{G42}$$ (MW)	28.086789	30.238652	0.1279117	29.299195	90.865591	27.072279	100	36.768382	65.83
$${P}_{G46}$$ (MW)	44.170228	20.950867	34.607258	65.1051	49.196018	29.311559	45.466769	16.783503	20.39
$${P}_{G49}$$ (MW)	25.479106	55.049438	21.463662	35.100865	49.519175	19.159563	30.349455	19.998876	219.98
$${P}_{G54}$$ (MW)	171.03288	152.74633	172.85744	138.82457	120.32187	152.00955	138.22158	112.96108	76.98
$${P}_{G55}$$ (MW)	46.632883	63.884832	57.206871	33.155876	27.620499	57.726208	116.18878	85.52397	50.00
$${P}_{G56}$$ (MW)	43.696699	74.4453	3.78E-20	37.843095	31.805611	28.190215	5.17E+00	5.75E+01	51.73
$${P}_{G59}$$ (MW)	39.762611	42.846159	99.950554	70.855249	66.012402	34.325836	100	28.925178	132..86
$${P}_{G61}$$ (MW)	129.52251	116.12683	76.658674	166.07081	193.92364	92.479551	242.27976	157.45117	120.23
$${P}_{G62}$$ (MW)	103.05631	36.190765	138.68366	114.25976	95.542923	135.76617	41.185466	166.30814	32.06
$${P}_{G65}$$ (MW)	39.369363	25.349497	23.846489	55.672231	28.136807	21.024433	78.657468	21.241019	240.04
$${P}_{G66}$$ (MW)	298.75103	262.11184	341.99049	199.24584	72.566588	275.76712	1.7263742	191.18238	170.77
$${P}_{G69}$$ (MW)	284.11136	36.256137	283.98504	220.86191	276.87909	286.24061	318.82676	296.49002	342.23
$${P}_{G70}$$ (MW)	24.420847	58.530444	54.851824	53.777007	0.7200069	9.8963742	27.838283	41.434461	47.94
$${P}_{G72}$$ (MW)	35.806337	13.273314	23.263764	40.71018	23.429675	29.263315	36.307227	32.33344	55.09
$${P}_{G73}$$ (MW)	22.949981	32.829655	34.343879	58.593999	38.352759	19.58988	68.374703	63.458191	54.80
$${P}_{G74}$$ (MW)	61.679556	34.968754	28.480787	26.208667	44.383678	68.625131	56.835762	4.283734	45.34
$${P}_{G76}$$ (MW)	72.271418	86.228001	49.190982	34.899477	34.458189	64.626361	54.049394	56.806653	53.51
$${P}_{G77}$$ (MW)	22.968618	21.404284	2.17E−05	26.042217	66.256809	35.426756	4.72E+01	2.82E+01	48.16
$${P}_{G80}$$ (MW)	309.36318	385.2597	329.82833	298.5911	366.15764	384.16613	32.524339	385.46092	332.42
$${P}_{G85}$$ (MW)	2.69E+01	6.52E+00	2.50E−02	5.51E+01	6.81E+01	5.11E+01	4.76E+01	4.52E+01	41.58
$${P}_{G87}$$ (MW)	10.411067	1.5478632	3.0227718	16.559781	77.918609	5.1554427	44.379518	4.7550816	1.90
$${P}_{G89}$$ (MW)	310.416	327.15864	406.90523	323.12488	39.590487	373.11708	68.420195	329.78795	210.71
$${P}_{G90}$$ (MW)	28.647952	30.358655	20.943072	31.490746	65.53683	14.564027	63.223173	5.6993441	23.19
$${P}_{G91}$$ (MW)	36.594081	67.953096	0.5205968	34.553001	1.3003347	9.2421047	55.935017	61.352129	45.22
$${P}_{G92}$$ (MW)	54.511709	1.3701922	21.364362	34.566542	92.135365	49.228727	20.0611	37.708013	41.92
$${P}_{G99}$$ (MW)	52.752491	14.950853	32.600003	43.432928	46.268455	20.68691	88.030205	67.258764	9.86
$${P}_{G100}$$ (MW)	133.53893	177.73846	192.59437	145.93183	240.75949	179.14495	232.19815	176.67993	166.02
$${P}_{G103}$$ (MW)	22.090747	89.441876	37.813647	82.066965	7.9019222	55.986594	0.7737086	59.304231	72.61
$${P}_{G104}$$ (MW)	14.273216	77.872717	39.411678	24.811601	17.394399	29.288941	2.2671886	65.607451	62.06
$${P}_{G105}$$ (MW)	32.027813	62.589341	28.136218	10.817359	19.18079	39.169855	85.048863	10.402192	56.43
$${P}_{G107}$$ (MW)	42.421291	56.626913	54.491021	32.472667	71.086143	28.126646	81.344505	45.104053	42.68
$${P}_{G110}$$ (MW)	39.133718	30.492124	25.65837	20.107864	2.9827881	13.937075	64.539857	27.406063	45.99
$${P}_{G111}$$ (MW)	46.772592	19.666794	34.048531	62.964561	67.095646	35.003227	70.964034	94.498892	21.59
$${P}_{G112}$$ (MW)	34.307033	1.5027623	43.304354	57.455894	89.57808	52.389674	82.141071	29.356489	54.83
$${P}_{G113}$$ (MW)	18.859456	41.155896	30.000843	10.499888	11.97101	25.836736	1.6570369	18.929262	56.99
$${P}_{G116}$$ (MW)	46.641469	31.761899	33.175967	28.198132	65.744664	16.191412	3.9474418	9.8490344	6.02
$${V}_{1}$$ (pu)	0.9755947	1.015563	0.972368	0.9678034	1.0307964	0.9818775	0.94	1.0028985	0.98
$${V}_{4}$$ (pu)	1.0037545	1.0107167	0.9915827	0.9984153	0.9796535	1.0184511	1.06	1.0075327	1.00
$${V}_{6}$$ (pu)	0.9930883	1.0107404	0.9790376	1.0009824	1.0226205	0.9968005	1.06	1.0033096	1.01
$${V}_{8}$$ (pu)	1.001848	1.0107353	0.9838811	1.0139053	1.0582519	1.0103585	0.94	1.0141423	0.98
$${V}_{10}$$ (pu)	1.0130388	1.0107393	0.9833819	1.0223518	1.0394668	1.0206254	0.9559686	1.0029636	1.00
$${V}_{12}$$ (pu)	0.9990578	1.0107102	0.990659	0.9957636	1.0391086	0.9901969	1.06	1.0032827	1.00
$${V}_{15}$$ (pu)	1.0077276	1.0107208	0.9749551	0.9931806	1.0234861	1.0025749	1.0158677	1.0015763	0.99
$${V}_{18}$$ (pu)	1.0175008	1.0107335	0.9817632	0.9904964	0.95216	1.0139521	1.06	1.0012431	0.99
$${V}_{19}$$ (pu)	1.0072259	1.0120977	0.9754394	0.9877359	0.9690685	1.0051226	1.06	1.0033828	0.99
$${V}_{24}$$ (pu)	0.9868438	1.0107109	0.9991512	0.9667762	1.0223629	1.0339587	1.06	1.0033885	0.99
$${V}_{25}$$ (pu)	1.0004621	1.0106932	0.9859443	1.0053381	0.9674887	0.9995287	0.9797086	1.0048057	1.02
$${V}_{26}$$ (pu)	1.0239666	1.0125367	0.9742331	0.9842713	0.9504169	0.9964773	0.9672845	1.0023007	0.99
$${V}_{27}$$ (pu)	1.0099687	1.0107504	1.0254128	0.9988385	0.9938304	0.9926038	1.0599755	1.0075133	1.00
$${V}_{31}$$ (pu)	1.0117357	1.0107365	0.9987288	1.0062992	1.0118899	1.0042962	1.0425167	1.003698	1.01
$${V}_{32}$$ (pu)	1.0055948	1.0107625	1.0082739	0.9960715	0.9767095	0.9926645	1.06	1.0008036	0.99
$${V}_{34}$$ (pu)	1.0093233	1.0107218	0.9900721	1.002729	0.989945	0.9904552	1.0275926	1.0063636	1.01
$${V}_{36}$$ (pu)	1.0043946	1.0107173	0.9847753	0.9997235	0.9935939	0.9845946	1.0580623	1.0019018	1.01
$${V}_{40}$$ (pu)	1.0145578	1.0107049	0.9824566	0.9950745	1.0268603	0.9980806	0.94	1.004353	0.99
$${V}_{42}$$ (pu)	1.0097933	1.0107262	0.9826538	0.9708156	1.0484933	0.9990177	0.9400494	1.0030512	1.00
$${V}_{46}$$ (pu)	0.9765032	1.015024	0.9423184	0.990603	0.9614848	1.0110713	1.06	1.0130523	0.99
$${V}_{49}$$ (pu)	1.0087427	1.0107596	0.9522425	1.0006226	1.043314	0.9994073	1.06	1.006489	1.03
$${V}_{54}$$ (pu)	1.0186545	1.010725	0.9854332	0.9805281	0.946709	0.9990618	0.94	1.0034572	1.04
$${V}_{55}$$ (pu)	1.0164577	1.0107313	0.9794105	0.9777562	0.9729364	0.9967516	0.94	1.003249	1.03
$${V}_{56}$$ (pu)	1.0152935	1.0107518	0.9812836	0.9779015	0.9571829	0.9960331	0.94	1.0017566	1.03
$${V}_{59}$$ (pu)	1.0120328	1.0140715	1.0033543	0.9883887	1.008423	0.9934477	0.94	1.0028546	1.00
$${V}_{61}$$ (pu)	1.0076086	1.0107254	1.0164649	0.9950227	0.9947235	0.989046	1.0326911	1.0028546	0.99
$${V}_{62}$$ (pu)	1.0050346	1.0125743	1.0047093	0.9909107	0.9919765	0.9787503	1.0337894	1.0035799	0.98
$${V}_{65}$$ (pu)	1.0222197	1.0107256	0.9992318	1.0017812	1.0078381	1.0072969	1.06	1.007566	1.00
$${V}_{66}$$ (pu)	1.0138962	1.0154207	0.9946429	1.0052744	0.9559682	0.9866618	1.0239445	1.013851	1.03
$${V}_{69}$$ (pu)	1.042357	1.010736	0.9939733	1.0393596	1.0242939	1.0412257	1.0575811	1.0025165	1.01
$${V}_{70}$$ (pu)	1.0130808	1.0107575	0.9905907	1.0065353	1.0309907	1.0110704	1.0016933	1.0037228	1.03
$${V}_{72}$$ (pu)	1.0025594	1.0107261	0.9707553	0.9784075	1.0013086	1.0209373	1.0190739	1.0102543	0.99
$${V}_{73}$$ (pu)	1.0130594	1.0107785	1.0102506	1.0336309	0.9451181	1.0030994	0.94	1.0015605	1.06
$${V}_{74}$$ (pu)	1.0085043	1.0106968	0.9692249	0.9772556	0.9775804	1.0073711	0.94	1.0067351	1.00
$${V}_{76}$$ (pu)	0.9954803	1.0107309	0.9594846	0.9543715	1.0258366	0.9958658	0.9627969	1.0031265	0.98
$${V}_{77}$$ (pu)	1.0111951	1.0129077	0.9983227	0.9908284	1.0078177	0.9962884	0.94	0.9992381	0.99
$${V}_{80}$$ (pu)	1.0250285	1.0107211	1.0440151	1.0152545	0.9956994	0.9984555	0.94	1.003369	1.01
$${V}_{85}$$ (pu)	1.0012245	1.0107397	1.0132613	0.9858348	0.9797477	0.9947601	0.9829425	1.0032828	0.99
$${V}_{87}$$ (pu)	1.006652	1.01072	0.9837245	0.9966944	1.0102235	1.0505185	0.9452304	1.0024074	0.98
$${V}_{89}$$ (pu)	0.9901991	1.0107344	1.0468375	0.9947819	1.0382378	0.9713792	1.06	1.0108695	1.00
$${V}_{90}$$ (pu)	1.0032796	1.0119949	1.008365	1.0165073	0.9668342	0.9772183	0.94	1.0024562	1.01
$${V}_{91}$$ (pu)	1.0307402	1.0107125	0.9969373	0.999607	0.9515844	1.0052582	1.06	1.0038764	0.99
$${V}_{92}$$ (pu)	1.0129018	1.0107204	0.992354	0.9824628	0.9802555	0.9781234	0.94	1.0018415	0.98
$${V}_{99}$$ (pu)	0.9913718	1.0109658	0.9936652	0.9991168	0.952732	0.9917847	0.94	1.003235	1.01
$${V}_{100}$$ (pu)	1.0318508	1.0107373	0.9959108	0.9982472	0.9816469	0.9994647	1.0310818	1.0060341	0.99
$${V}_{103}$$ (pu)	1.0155488	1.0112627	0.9959641	0.9983592	0.9889007	1.0100378	1.06	1.0038077	1.02
$${V}_{104}$$ (pu)	1.0071165	1.0107182	0.9879583	0.9972382	0.9765162	0.9997485	1.0044333	1.0046284	1.02
$${V}_{105}$$ (pu)	1.0090936	1.0107395	0.9909608	0.9966941	0.9510039	0.9969756	1.06	1.0046202	1.02
$${V}_{107}$$ (pu)	1.0219929	1.0107689	1.011368	1.0028014	1.0448404	0.9979651	1.06	1.0035749	1.00
$${V}_{110}$$ (pu)	1.0041077	1.0107085	0.981842	0.9874705	1.030782	0.9981363	1.0318386	1.0047424	0.99
$${V}_{111}$$ (pu)	1.0098048	1.0123173	0.9967582	0.9881657	1.0354325	1.0010923	1.06	1.002754	0.99
$${V}_{112}$$ (pu)	0.9896521	1.0107238	0.9678187	0.9986953	1.006486	1.0048775	0.9958937	1.0016868	1.03
$${V}_{113}$$ (pu)	1.0162678	1.0107178	0.989258	0.9846165	1.0501106	1.0048607	0.9958676	1.0034255	1.01
$${V}_{116}$$ (pu)	0.9723242	1.0107233	0.970682	1.0144823	0.9813626	1.0239536	1.06	1.007034	0.99
$${T}_{8}$$ (8–5)	0.9969683	0.974166	1.0028485	1.014071	0.9904684	0.9831192	0.9	1.0138265	1.00
$${T}_{32}$$ (26–25)	1.0608599	0.9861295	1.0164473	0.991363	1.0432633	1.0214152	1.0421361	0.9927923	1.02
$${T}_{36}$$ (30–17)	0.9858643	1.0307987	1.01755	0.9396623	0.9193303	0.9655758	0.9	0.9886933	1.02
$${T}_{51}$$ (38–37)	0.9879601	0.9761415	0.9798519	0.965365	1.076739	1.0208299	0.9	1.0007664	0.95
$${T}_{93}$$ (63–59)	1.0010503	0.9832594	0.9703067	0.9986791	1.0633998	0.9435669	1.1	1.0103129	1.01
$${T}_{95}$$ (64–61)	1.0172656	1.0078413	0.973569	0.9556252	0.9078842	0.9608501	1.1	0.989212	0.99
$${T}_{102}$$ (65–66)	0.9934653	0.9751778	1.0129403	0.9702031	1.0211318	1.0031683	1.0528714	0.9801001	0.99
$${T}_{107}$$ (68–69)	0.9809535	1.0271104	0.9863273	0.9862177	1.0730421	1.0054443	0.9	1.0069097	0.98
$${T}_{127}$$ (81–80)	1.061382	0.99588	0.9453683	0.9584389	1.0264662	0.9801847	1.1	0.9743607	0.96
$${Q}_{34}$$ (MVAR)	14.853535	17.753942	11.183449	9.8051439	16.769534	10.749402	5.5676094	22.276187	13.97
$${Q}_{44}$$ (MVAR)	8.6918036	19.665199	12.052902	6.9347835	2.7199204	17.254878	18.111223	18.063734	11.12
$${Q}_{45}$$ (MVAR)	10.84757	1.2307905	11.55766	5.6683816	10.090073	15.948375	12.740237	21.518359	23.03
$${Q}_{46}$$ (MVAR)	14.524919	26.204434	9.6965216	7.4479659	0.0223675	14.457601	2.4888498	9.5501911	16.69
$${Q}_{48}$$ (MVAR)	11.628841	18.691988	13.917422	13.394523	24.976169	14.374375	26.25648	20.927619	19.83
$${Q}_{74}$$ (MVAR)	14.437324	18.73354	13.96664	8.4089813	11.720403	17.894635	12.985434	16.288014	10.40
$${Q}_{79}$$ (MVAR)	18.384129	10.75443	13.046392	21.573761	23.911704	8.6485609	7.2860588	16.674911	9.23
$${Q}_{82}$$ (MVAR)	21.614886	16.891906	22.644904	8.5475088	29.984245	14.180096	22.253465	1.6265926	11.45
$${Q}_{83}$$ (MVAR)	22.180259	18.090222	12.870701	16.550216	24.22214	19.879856	6.6839413	0.9938805	15.64
$${Q}_{105}$$ (MVAR)	15.942382	11.075106	11.801482	7.3126552	18.910879	6.3138873	30	6.09791	16.78
$${Q}_{107}$$ (MVAR)	16.104502	18.131344	15.399917	13.560577	8.2444647	20.712886	26.397513	9.0391943	27.74
$${Q}_{110}$$ (MVAR)	12.902698	24.836424	9.4198206	7.4903267	2.0881328	13.281062	10.239639	12.192777	22.42
Objective function	134,581.11	147,663.18	133,257.99	137,402.63	431,355.38	133,921.61	431,849.5	143,003.58	140,575.3099
Fuel cost ($/h)	134,270.00	140,040.00	132,960.00	137,170.00	160,430.00	133,330.00	158,170.00	137,670.00	137,617.0912
Power losses (MW)	69.216576	68.925131	80.478981	66.058629	96.068573	82.104475	127.43309	79.604315	58.8779
Voltage deviation (pu)	0.5839816	0.5226177	1.2824171	0.9787408	1.2948132	0.9143519	2.7579362	0.5040643	0.7335
Iterations time (s)	197.1166	4716.907	618.2695	399.4292	204.7562	207.9040	219.67135	269.45299	–

Further, a Wilcoxon signed rank sum test has been executed to compare performance between proposed algorithms. Thirty independent runs are implemented in the test. The selected level of significance is 5%. The $p$-values determined by Wilcoxon’s rank-sum test are shown in Table 22. The ${H}_{0}$ values obtained from the test is “0” meaning the null hypothesis is rejected among the optimization algorithms for most cases except case 2 and case 3, where the mAHA and RUN perform equally. In the leftover cases, mAHA is found to be excellent. It can be concluded from the test results that the mAHA is a choice to the other optimization methods when applied to solve the OPF problems under several cases.

**Table 22:** Wilcoxon signed-rank sum test for IEEE 118 bus test system.

Cases	mAHA vs. AHA		mAHA vs. HHO		mAHA vs. RUN		mAHA vs. SCA		mAHA vs. SMA		mAHA vs. TSA		mAHA vs. WOA	
	$\mathrm{p}$-value	${\mathrm{H}}_{0}$	$\mathrm{p}$-value	${\mathrm{H}}_{0}$	$\mathrm{p}$-value	${\mathrm{H}}_{0}$	$\mathrm{p}$-value	${\mathrm{H}}_{0}$	$\mathrm{p}$-value	${\mathrm{H}}_{0}$	$\mathrm{p}$-value	${\mathrm{H}}_{0}$	$\mathrm{p}$-value	${\mathrm{H}}_{0}$
Case 1	9.8524e−07	0	8.8966e−07	0	.8966e−07	0	8.8966e−07	0	3.2293e−05	0	8.8966e−07	0	8.8966e−07	0
Case 2	4.5554e−05	0	8.8966e−07	0	0.1760	1	8.8966e−07	0	0.0347	0	8.8966e−07	0	8.8966e−07	0
Case 3	4.7702e−06	0	8.8966e−07	0	0.4005	1	8.8966e−07	0	0.0186	0	8.8966e−07	0	8.8966e−07	0
Case 4	1.2068e−06	0	8.8966e−07	0	8.8966e−07	0	8.8966e−07	0	5.7674e−06	0	8.8966e−07	0	8.8966e−07	0

Table 23 illustrates comparative results for minimizing the fuel cost (Case 1), power losses (Case 2), voltage deviation (Case 3), and multi-objective function (Case 4) with several other algorithms which are developed SDO, LSDO, PSOIWA, PSOCFA, RGA, BBO, MSA, ABC, CSA, GWO, BSOA, and MJAYA^67–69^. As shown, the proposed mAHA obtain the minimum objective function for all cases among other techniques.

**Table 23:** Comparison results for IEEE 118 bus test system.

	SDO^67^	LSDO^67^	mAHA				
Case 1: minimization of fuel cost							
Fuel cost ($/h)	139,923.69	137,105.99	132,849.31				

	PSOIWA^68^	PSOCFA^68^	RGA^68^	BBO^68^	mAHA		
Case 2: minimization of active power losses							
Power losses (MW)	76.72	77.91	71.89	51.43	38.665089		

	PSOIWA^68^	PSOCFA^68^	RGA^68^	BBO^68^	mAHA		
Case 3: minimization of total voltage deviation							
Voltage deviation (pu)	1.104	1.0536	0.8839	0.4613	0.4264959		

	MSA^69^	ABC^69^	CSA^69^	GWO^69^	BSOA^69^	MJAYA^69^	mAHA
Case 4: minimization of multi-objective function							
Objective function	142,773.2738	149,342.2459	145,332.5903	146,012.7822	152,178.5959	140,575.3099	133,257.99
Fuel cost ($/h)	139,976.2843	144,826.2921	143,302.7261	144,359.2809	145,653.0011	137,617.0912	132,960.00
Power losses (MW)	62.4623	59.2971	61.5698	69.2275	64.3177	58.8780	80.478981
Voltage deviation (pu)	0.6927	0.9535	0.8346	0.7134	1.4163	0.7335	1.2824171

## Conclusion

This research develops mAHA, a novel optimizer for dealing with OPF issues, including fuel cost, power loss, voltage profile improvement, and emissions. Additionally, eight approaches for multi-objective and single-objective OPF were presented. The proposed methods were evaluated and confirmed on standard and modified IEEE 30 bus and IEEE 118 bus networks, among others. As a result, the results indicated that the optimum allocation of renewable energy sources (RES) concurrent with the OPF produces better results than if it happens separately. Distributed generation (DG) location and size were added as control variables. As a result, the OPF issue dimension was also expanded. In addressing the OPF optimization issue, mAHA demonstrated excellent performance and efficacy.

Additionally, the most promising results from IEEE Power Networks demonstrate the effectiveness of the suggested approach. Compared to other recent algorithms, the mAHA mitigated the objective functions better in all cases. Based on the comparison results in the case of IEEE 30 bus system, mAHA demonstrated an improvement reduction of single objective functions of 92.874% (Fuel cost), 80.254% (Power losses), and 91.49% (voltage deviation) when compared to AHA, HHO, RUN, SCA, SMA, TSA, WOA, and other published techniques. Furthermore, the comprehensive study of mAHA with the mentioned methodologies has shown that mAHA has met the minimum objective function of 864.735. Additionally, in comparison with the other algorithms, mAHA has the highest fuel cost reduction of 97.451% in the case of minimizing the fuel cost while simultaneously deploying renewable energy sources. As shown in the case of the IEEE 118 bus system, mAHA was superior to other optimizers in finding the global optimum solution of the objective function cases.

Therfore, it is clear that the mAHA outperformed these recent algorithms irrespective of their objective functions, which shows that the mAHA is capable of solving other real-life applications. The OPF problem can be solved by incorporating RES uncertainties in future work for handling as a real problem. Also, the suggested mAHA can be modified or mixed with other metaheuristic algorithms in upcoming work to address other complex optimization problems in dissimilar fields, for example, optimally allocated generation when RES are vague, optimal hybrid RES planning, estimating fuel cell parameters, and modeling photovoltaic systems.

## Data Availability

The datasets used and analyzed during the current study are available from the corresponding author upon reasonable request.
